# Heterologous ChAdOx1/BNT162b2 vaccination induces stronger immune response than homologous ChAdOx1 vaccination: The pragmatic, multi-center, three-arm, partially randomized HEVACC trial

**DOI:** 10.1016/j.ebiom.2022.104073

**Published:** 2022-05-23

**Authors:** Zoltán Bánki, Jose Mateus, Annika Rössler, Helena Schäfer, David Bante, Lydia Riepler, Alba Grifoni, Alessandro Sette, Viviana Simon, Barbara Falkensammer, Hanno Ulmer, Bianca Neurauter, Wegene Borena, Florian Krammer, Dorothee von Laer, Daniela Weiskopf, Janine Kimpel, Hevacc Study Group

**Affiliations:** a Institute of Virology, Department of Hygiene, Microbiology and Public Health, Medical University of Innsbruck, Peter-Mayr-Str. 4b, 6020 Innsbruck, Austria; b Center for Infectious Disease and Vaccine Research, La Jolla Institute for Immunology (LJI), La Jolla, CA 92037, USA; c Department of Medicine, Division of Infectious Diseases and Global Public Health, University of California, San Diego (UCSD), La Jolla, CA 92037, USA; d Department of Microbiology, Icahn School of Medicine at Mount Sinai, New York, NY 10029, USA; e Department of Pathology, Icahn School of Medicine at Mount Sinai, New York, NY 10029, USA; f Division of Infectious Diseases, Department of Medicine, Icahn School of Medicine at Mount Sinai, New York, NY 10029, USA; g The Global Health and Emerging Pathogen Institute, Icahn School of Medicine at Mount Sinai, New York, NY 10029, USA; h Department of Medical Statistics, Informatics and Health Economics, Medical University of Innsbruck, Austria

**Keywords:** Heterologous COVID-19 vaccination, SARS-CoV-2, ChAdOx1, BNT162b2, Neutralizing antibodies, T cells

## Abstract

**Background:**

Several COVID-19 vaccines have been approved. The mRNA vaccine from Pfizer/BioNTech (Comirnaty, BNT162b2; BNT) and the vector vaccine from AstraZeneca (Vaxzevria, ChAdOx1; AZ) have been widely used. mRNA vaccines induce high antibody and T cell responses, also to SARS-CoV-2 variants, but are costlier and less stable than the slightly less effective vector vaccines. For vector vaccines, heterologous vaccination schedules have generally proven more effective than homologous schedules.

**Methods:**

In the HEVACC three-arm, single-blinded, adaptive design study (ClinicalTrials.gov Identifier: NCT04907331), participants between 18 and 65 years with no prior history of SARS-CoV-2 infection and a first dose of AZ or BNT were included. The AZ/AZ and the AZ/BNT arms were randomized (in a 1:1 ratio stratified by sex and trial site) and single-blinded, the third arm (BNT/BNT) was observational. We compared the reactogenicity between the study arms and hypothesized that immunogenicity was higher for the heterologous AZ/BNT compared to the homologous AZ/AZ regimen using neutralizing antibody titers as primary endpoint.

**Findings:**

This interim analysis was conducted after 234 participants had been randomized and 254 immunized (N=109 AZ/AZ, N=115 AZ/BNZ, N=30 BNT/BNT). Heterologous AZ/BNT vaccination was well tolerated without study-related severe adverse events. Neutralizing antibody titers on day 30 were statistically significant higher in the AZ/BNT and the BNT/BNT groups than in the AZ/AZ group, for B.1.617.2 (Delta) AZ/AZ median reciprocal titer 75.9 (99.9% CI 58.0 - 132.5), AZ/BNT 571.5 (99.9% CI 396.6 - 733.1), and BNT/BNT 404.5 (99.9% CI 68.3 - 1024). Similarly, the frequency and multifunctionality of spike-specific T cell responses was comparable between the AZ/BNT and the BNT/BNT groups, but lower in the AZ/AZ vaccinees.

**Interpretation:**

This study clearly shows the immunogenicity and safety of heterologous AZ/BNT vaccination and encourages further studies on heterologous vaccination schedules.

**Funding:**

This work was supported by the Medical University of Innsbruck, and partially funded by NIAID contracts No. 75N9301900065, 75N93021C00016, and 75N93019C00051.

**Research in context**

**Evidence before this study**

We searched pubmed and pre-print servers like medRxiv using the keywords “COVID-19 heterologous vaccination”. In retrospective or prospective, observational studies the heterologous COVID-19 vaccination regimen using the vector vaccine from AstraZeneca (Vaxzevria, ChAdOx1; AZ) as prime and the mRNA vaccine from Pfizer/BioNTech (Comirnaty, BNT162b2; BNT) as boost has recently been compared to either one or both of the corresponding homologous regimens. These studies had been conducted in response to rare thrombotic events upon AZ vaccination in individuals that previously received an AZ boost. Mostly, only BNT boost immunizations were included due to safety concerns or participants had the choice between the AZ and BNT boost. In addition, results for two randomized studies have been published so far, the CombiVacS and the Com-COV study. While the CombiVacS study compares the heterologous AZ/BNT vaccination with individuals that received only a prime with AZ and were not boosted, the Com-COV study compares both orders of heterologous vaccination (AZ/BNT and BNT/AZ) with both homologous vaccination regimens (AZ/AZ and BNT/BNT). All studies show the heterologous AZ/BNT vaccination to be superior to homologous AZ/AZ immunization or prime only and to be similar to homologous BNT/BNT immunization regarding immunogenicity.

**Added value of this study**

In our study, we not only analyzed antibody but also vaccine induced T cell responses in more depth compared to previously published studies. We characterized in detail the magnitude as well as functionality of vaccine induced T cell responses which has not been done in previous studies. We found that stronger and more multifunctional T cell responses in heterologous vaccine (AZ/BNT) arm compared to the homologous AZ/AZ arm. Beside the characterization of spike specific responses, we included characterization of responses against variants of concern and variants of interest and found the heterologous AZ/BNT regimen to induce statistically significant higher antibody and T cell responses. In contrast to some of the previously published studies our study includes both heterologous (AZ/BNT) and homologous control groups (AZ/AZ and BNT/BNT). Individuals with an AZ prime were randomized to the AZ/AZ or the AZ/BNT arm stratified by gender and study site. This separates our study clearly from the observational studies. The only randomized study which has been published and includes both homologous control groups, the Com-COV study has two major differences compared to our study as the interval between prime and boost was shorter (4 weeks) and older individuals were included (50-70 years). Therefore, our study with a longer prime/boost interval, a younger study population and a more detailed analysis of immune responses complements and extends the Com-COV study.

**Implications of all the available evidence**

COVID-19 vector vaccines are less expensive and easier to store and transport compared to mRNA vaccines. However, they have the disadvantage of a lower efficacy. A heterologous AZ/BNT vaccination as described here could combines advantage from both classes of vaccines. This could especially be important as first studies report immunity after BNT immunization to decrease rater quick. Further follow-up of our cohort will be needed to compare durability of responses for the different immunization regimens.

Alt-text: Unlabelled box

## Introduction

After more than 2 years and despite massive non-pharmaceutical interventions, the coronavirus disease 2019 (COVID-19) pandemic is still ongoing. Broad vaccination programs are the only option for permanent virus control and indeed several effective vaccines are already in use. The novel mRNA vaccines, Comirnaty (BioNTech/Pfizer; BNT) and Spikevax (Moderna), have proven to be highly effective with over 90% protection against COVID-19 with pre-omicron variants. However, they are relatively expensive and need to be stored and distributed frozen. Additionally, vaccine efficacy waned over time and protection against the B.1.1.529 (Omicron) variant by antibodies is considerably lower.^1^ The vector vaccines, such as the chimpanzee adenovirus-based Vaxzevria (Astrazeneca; AZ) vaccine, have shown somewhat lower effectiveness, especially against immune escape variants, but are less costly and more temperature stable.^2^, ^3^, ^4^ Furthermore, the use of AZ has been discontinued primarily in young women in several countries, because of the risk of rare thromboembolic events after vaccination.^5^ As a consequence many especially young AZ-primed individuals received their second dose vaccination with an mRNA vaccine instead of AZ.

Starting late 2020, novel variants, termed variants of concern (VoC), of severe acute respiratory syndrome coronavirus 2 (SARS-CoV-2) have emerged that are either more transmissible (B.1.1.7, Alpha) and partially less susceptible to the antiviral immune response (B.1.351 Beta, P.1 Gamma etc.). In summer 2021, the B.1.617.2 (Delta) variant, which is even more transmissible than B.1.1.7, became dominant in many countries. Although vaccine-induced antibodies neutralize B.1.617.2 less efficiently than B.1.1.7, the vaccines still seem to provide good protection, with somewhat lower effectiveness of the vector vaccines. Recently, the B.1.1.529 2 variant emerged, which shows the strongest immune escape so far seen in SARS-CoV-2 variants.^6^, ^7^, ^8^, ^9^ Meanwhile, B.1.1.529 and its subvariants replaced B.1.617.2 in most countries.

For vector vaccines in general, homologous vaccination is often found to be less effective than heterologous prime/boost regimen, either combining two different vaccine vectors or a vector with a non-vector vaccine. This is not only true for viruses such as human immunodeficiency virus 1 (HIV-1) where many different viral vector/non-vector combinations have been explored in pre-clinical and clinical trials but also for COVID-19 animal models.^10^^,^^11^ A potential reason for the limited immunogenicity of homologous viral vector vaccination regimens is the interference of anti-vector neutralizing antibody and T cell response induced upon prime immunization which can diminish the effect of the booster dose. We therefore proposed that a heterologous prime/boost with an AZ vector prime followed by the BNT mRNA boost would induce higher titers of SARS-CoV-2 neutralizing antibodies, especially against VoC, and T cells compared to a homologous AZ/AZ regimen.

Here, we compared the reactogenicity as well as the T-cell and antibody responses to prototypic and variant SARS-CoV-2 induced by heterologous AZ/BNT vaccination relative to the corresponding homologous regimens.

## Methods

### Ethics statement and study design

**Figure 1:**
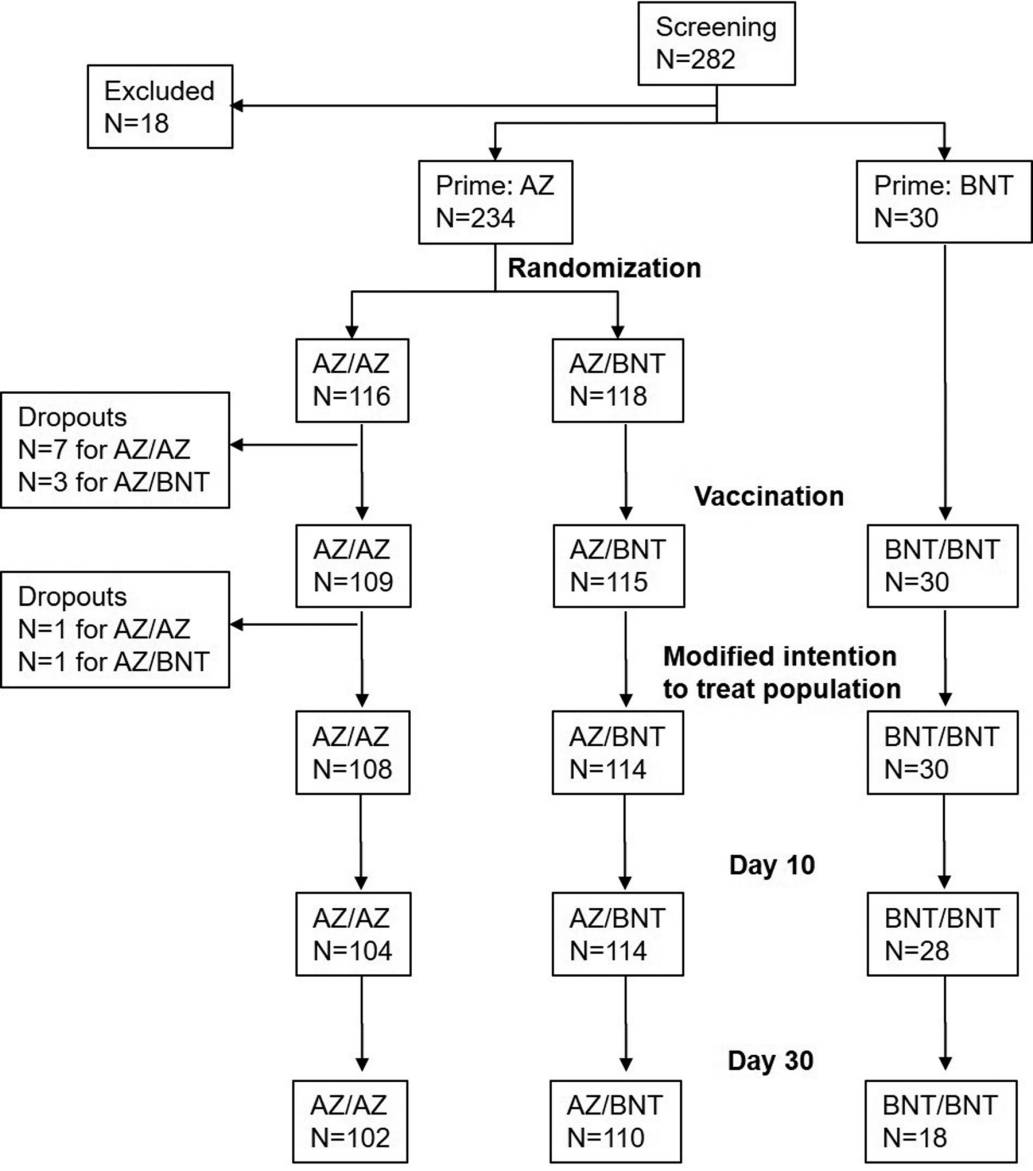
**Patient recruitment.** After screening N=18 participants were excluded due to previous infection, not given consent etc. Participants (N=234) who received an AZ prime were randomized to the AZ/AZ or the AZ/BNT arm. Due to organizational reasons (ordering of vaccine doses etc.) randomization was performed 2-4 days prior to vaccination. N=7 for AZ/AZ and N=3 for AZ/BNT dropped out after randomization but prior to vaccination as they meanwhile received their second dose of vaccine outside the study. These participants did not know at the time point of drop-up which vaccine they would have received in the study. Each of the remaining participants was vaccinated according to their randomization. N=1 participant for the AZ/AZ and AZ/BNT arm each dropped out immediately after boost vaccination reducing the subgroup included in follow-up analysis for safety and immunogenicity to N=108 for AZ/AZ and N=114 for AZ/BNT (modified intention to treat population). Some participants have not yet reached day 30 post boost (N=5 for AZ/AZ, N=3 for AZ/BNT, N= 10 for BNT/BNT). Some participants were unavailable on day 10 (N=4 for AZ/AZ, N=0 for AZ/BNT, N= 2 for BNT/BNT) or day 30 (N=1 for AZ/AZ, N=1 for AZ/BNT, N=2 for BNT/BNT). These participants were not excluded from the study but re-invited for the next study visit. However, this reduced the number of participants analyzed per time point compared to the total number of participants within a group. Numbers of participants per group which are analyzed for day 10 and day 30 are shown in the figure.

The HEVACC (Heterologous SARS-CoV-2 Vaccination with ChAdOx-1 and BNT162b2) study is a multi-center (Innsbruck/Kufstein/Schwaz), single-blinded, three-arm, partially randomized clinical trial and has been approved by the ethics committee of the Medical University of Innsbruck (EC: 1191/2021) and the Federal Office for Safety in Health Care (BASG); EudraCT Number: 2021-002171-19; ClinicalTrials.gov Identifier: NCT04907331. Patient recruitment started on 11^th^ of May shortly after approval from national authorities and ethics committee had been granted. However, due to technical problems the study was registered not until 25^th^ of May at ClinicalTrials.gov. All participants provided inform consent prior to inclusion into the study. All experiments performed at the La Jolla Institute (LJI) were approved by the institutional review boards (IRB) of the La Jolla Institute (IRB#: VD-247). The HEVACC trial has a parallel group design. Two of the three arms, the AZ/AZ and the AZ/BNT arm, were randomized, while the third arm (BNT/BNT) was observational. All subjects who have already received AZ as prime shot were randomized to the second dose AZ or BNT group. Subjects who have received BNT as prime were vaccinated again with BNT. As subjects were included into the trial as per protocol after they already received the first immunization only the AZ/AZ and the AZ/BNT arms could be randomized. Randomization of the AZ/AZ and the AZ/BNT arms was performed in a 1:1 ratio stratified by study site (Innsbruck/Kufstein/Schwaz) and sex (male/female). Randomization code has been generated by the study statistician using permutated blocks using Stata/MP 16.1. Randomization lists were provided by the statistician to the study monitor who performed randomization of participants after verification of inclusion and exclusion criteria as well as stratification status and after screening assessments have been performed. Due to organizational reasons (ordering of the correct number of vaccine doses etc.), randomization had to be performed a few days prior to vaccination. Some dropouts occurred between randomization and vaccination (N=7 for AZ/AZ and N=3 for AZ/BNT) as these participants meanwhile decided to receive their second dose of vaccine outside the study. These participants did not know at the time point of drop-up, which vaccine they would have received in the study. Each of the remaining participants was vaccinated according to their randomization (intention to treat population; N=109 for AZ/AZ, N=115 for AZ/BNT, N=30 for BNT/BNT). N=1 participant for the AZ/AZ and AZ/BNT arm each dropped out immediately after boost vaccination reducing the subgroup included in follow-up analysis for safety and immunogenicity to N=108 for AZ/AZ and N=114 for AZ/BNT (modified intention to treat population). Some participants have not yet reached day 30 post boost at the time point of interim analysis (N=5 for AZ/AZ, N=3 for AZ/BNT, N= 10 for BNT/BNT). Some participants were unavailable on day 10 (N=4 for AZ/AZ, N=0 for AZ/BNT, N= 2 for BNT/BNT) or day 30 (N=1 for AZ/AZ, N=1 for AZ/BNT, N=2 for BNT/BNT). These participants were not excluded from the study but re-invited for the next study visit. However, this reduced the number of participants analyzed per time point compared to the total number of participants within a group. Numbers of participants per group, which are analyzed for immunogenicity on day 10 and day 30 are shown in Figure 1. For safety analysis, there were no missing data as missing electronic diary entries were completed at the next in person study visit. Study participants of the AZ/AZ and the AZ/BNT arms were blinded regarding the type of second vaccination for 90 days following second vaccination. In a study amendment, the AZ/AZ arm was closed upon interim analysis. Additionally, a BNT booster immunization was added to the protocol.

### Inclusion and exclusion criteria

Participants between 18 and 65 years who had previously received one vaccination with AZ or BNT were eligible for inclusion. Exclusion criteria were a previous positive PCR-test result for SARS-CoV-2 or positive anti-SARS-CoV-2 nucleoprotein (N) antibody test, history of leukemia, lymphoma, or underlying bone marrow disorder (e.g., myelodysplasia, myeloma, myeloproliferative disorder) or bone marrow transplant, malignancy that required treatment with chemotherapy, immunotherapy, radiation therapy, or other antineoplastic target therapies within 24 months prior to study enrollment. Female participants of childbearing potential were only enrolled in the study if the participant had a negative urine pregnancy test at screening, agreed to practice adequate contraception from providing consent until 3 months after administration of study vaccine and were not currently breastfeeding.

### Interventions

**Figure 2:**
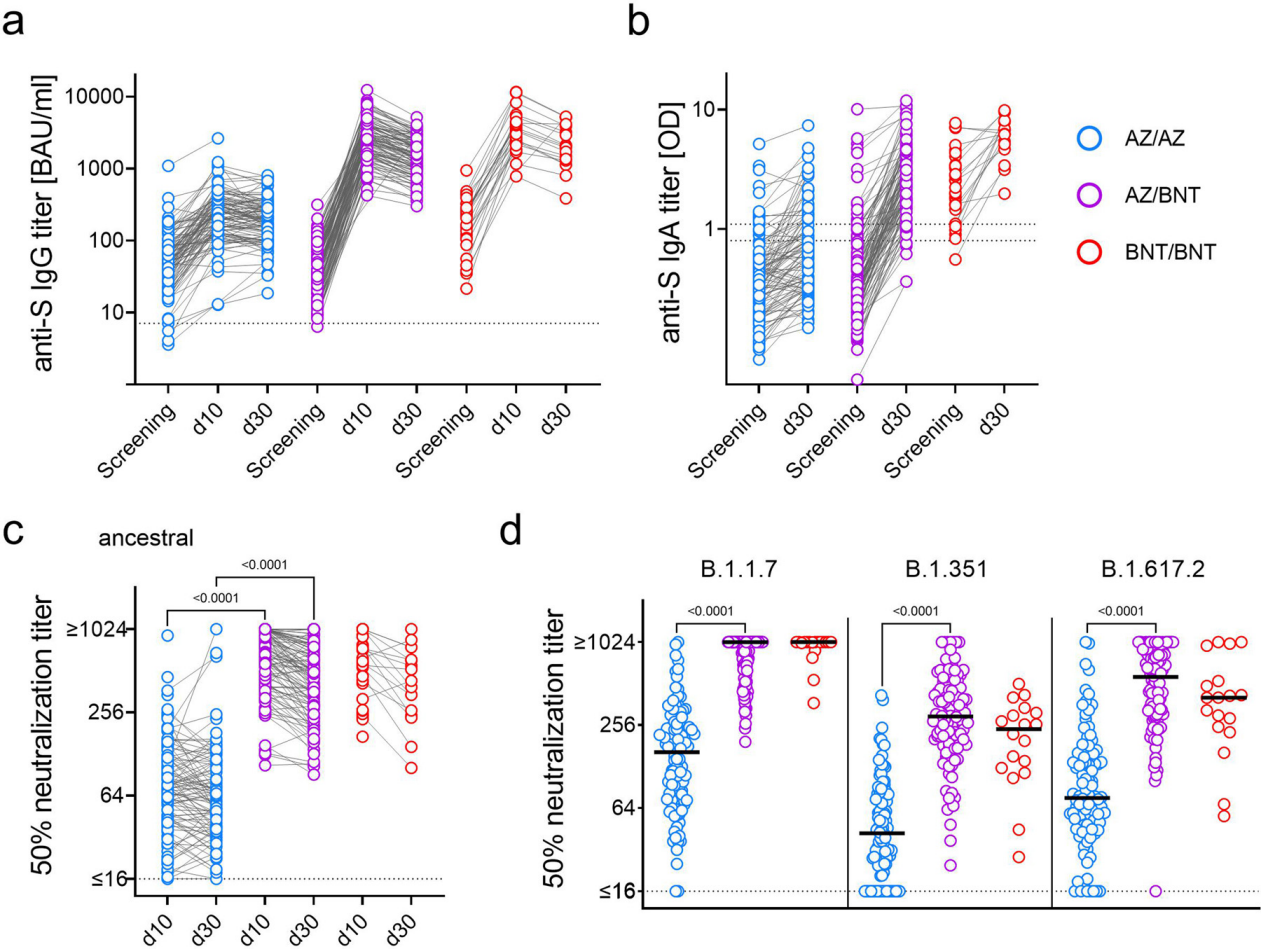
**Antibody responses are higher after heterologous vaccination compared to homologous AZ vaccination.** A. Titers of anti-S IgG at screening (3-7 days prior to boost), day 10 and day 30 post boost vaccination. Dotted line indicates detection limit (7.1 BAU/ml). B. Titers of anti-S IgA at screening and day 30 post boost vaccination. Values are expressed as optical density (OD). Dotted lines indicate cut-off values of the assay (< 0.8 negative; 0.8 – 1.1 borderline positive; > 1.1 positive). C. 50% neutralization titers as determined in a VSV pseudovirus neutralization assay using ancestral (wild type) spike. D. 50% neutralization titers against B.1.1.7, B1.351 and B.1.617.2 variants were quantified in a focus forming assay using replication competent SARS-CoV-2 isolates. Median and individual values are shown. For C and D, titers ≤ 1:16 were considered negative (indicated by the dotted line). Values <1:16 were set to 1:16 and values >1:1024 to 1:1024. Statistical differences were determined using Kruskal-Wallis test followed by uncorrected Dunn's comparison between AZ/AZ and AZ/BNT groups. A p value <0.001 was used as significance level according to stopping rule of the interim analysis as applied for the comparison of the primary endpoint neutralizing antibodies between AZ/AZ and AZ/BNT arms. All other comparisons were exploratory. 95 % confidence intervals (95 % CI) for binding antibodies and 99.9 % CI for neutralizing antibodies are shown in Supplementary Tables S3 and S4. AZ/AZ screening n=109, d10 n=104, d30 n=102; AZ/BNT screening n=115, d10 n=114, d30 n=110; BNT/BNT screening n=30, d10 n=28, d30 n=18.

A non-randomized group received a homologous BNT/BNT vaccination 3-6 weeks after the first BNT dose. Participants who had a first dose with AZ were randomized and either received the second dose with AZ (AZ/AZ) or BNT (AZ/BNT) 11-13 weeks after the prime immunization (Figure 1). Participants for all groups were recruited in parallel. For the AZ primed group participants were primarily recruited in groups prioritized according to the national vaccination program such as health care personnel, teachers, etc. All vaccines were administrated according to manufacturers´ recommendations regarding dosing and route of vaccination. Syringes were masked with a label containing the participant ID only to ensure blinding of participants regarding the type of vaccine applied (AZ/AZ and AZ/BNT arms). Blood was collected for ethylenediaminetetraacetic acid (EDTA)-plasma 3-7 days before vaccination (screening visit) and 2, 10±1, and 30±3 days after boost vaccination. On day 2 and day 10±1, an additional blood sample was collected for blood counts, blood chemistry and coagulation. From a subset of participants, blood for T cell analysis was collected on day 10±1 and day 30±3. Participants reported side-effects daily in an electronic diary for 7 days following the boost vaccination (Figure 2). Additionally, side-effects were inquired at each study visit for blood collection. Participants who missed a study visit were re-invited for the next scheduled study visit

### Primary and secondary endpoints

We hypothesized that heterologous AZ/BNT regimen was as least as immunogenic as homologous AZ/AZ regimen. To analyze this hypothesis primary endpoints of this study were defined as levels of neutralizing antibodies against wild type and immune escape variants. Additionally, T cell responses and reactogenicity including severe adverse events were analyzed as secondary endpoints in this interim analysis. The study will be continued to analyze further secondary endpoints of breakthrough infections on day 180 and clinical course of these breakthrough infections.

### Interim analysis

Two interim analyses for the primary endpoint (level of nAbs on day 30) had been scheduled when N=200 and when N=400 participants have been randomized. No bias adjustment to account for the interim analysis was planned. A first interim analysis applying the Haybittle Peto rule for early stopping of clinical trials was performed by the core study team and the study statistician after N=200 participants had been randomized and followed up to day 30 post vaccination for safety and immunogenicity. To minimize bias, data all data were reviewed by at least two persons independently and results were presented to the Data Safety Monitoring Board first. As the p-value between the two randomized arms was smaller than 0.001 regarding the primary endpoint (level of nAbs), the clearly inferior homologous AZ/AZ arm was closed following interim analysis as also recommended by the Data Safety Monitoring Board. All other comparisons were strictly exploratory. The trial will continue in an observational manner with two arms, AZ/BNT and BNT/BNT.

### Antibody responses

Technical details are found in the supplementary appendix. Binding antibodies were determined with commercial kits: anti-N with ElecsysAnti-SARS-CoV-2 (Roche Diagnostics, Indianapolis, USA), anti-S IgA (Euroimmun, Lübeck, Germany), anti-S IgG (Architect anti-SARS-CoV-2 IgG II Quant, Abbott). SARS-CoV-2 neutralizing antibodies (nAbs) were determined as described previously using a vesicular stomatitis pseudovirus assay (VSV, spike from ancestral wild type strain) or a focus forming assay with replication competent SARS-CoV-2.^12^ For the focus forming assay, titers against B.1.1.7 (isolate C69.1, EPI_ISL_3277382), B.1.351 (isolate C24.1, EPI_ISL_1123262) and B.1.617.2 (isolate: USA/NY-MSHSPSP-PV29995/2021, EPI_ISL_2290769) were determined. Continuous 50% neutralization titers were calculated using a nonlinear regression as described earlier.^13^ Titers >1:16 were considered as positive.

### T cell assays

T cell responses were measured by Interferon-γ release assay (IGRA; QuantiFERON (QFN) SARS-CoV-2 RUO IGRA (Qiagen)), activation induced markers (AIM) assay (percentage of AIM^+^ (OX40^+^CD137^+^) CD4^+^ and (CD69^+^CD137^+^) CD8^+^ T cells after stimulation of peripheral blood mononuclear cells (PBMCs) with peptide megapools^14^^,^^15^) or intracellular cytokine staining (ICS) assay (cytokine specific T cell responses upon stimulation with SARS-CoV-2 MPs^14^). See also Supplementary Methods for more details method description. Antibodies used and gating strategies are found in Supplementary Table S1 and Supplementary Figure S2.

### Peptide megapools (MP)

A previously developed approach allows simultaneous testing of a large number of peptides in small amounts of blood.^14^^,^^16^, ^17^, ^18^ To capture spike-specific responses, we utilized a MP of 253 overlapping peptides corresponding to the entire lengths of the spike protein of either the ancestral or variant sequences. As this peptide pool consists of peptides with a length of 15 amino acids, both CD4^+^ and CD8^+^ T cells have the capacity to recognize this MP.^15^^,^^19^ We have previously shown that these MPs are suitable to stimulate T cell responses from SARS-CoV-2-exposed or vaccinated individuals.^16^^,^^20^ The cytomegalovirus (CMV) MP, consisting of 313 HLA Class-I and Class-II epitopes was used as a representative ubiquitous pathogen in each experiments.^18^

### Statistics

Originally, 1000 participants were planned to be recruited in each of the three arms. The sample size was mainly based on the secondary endpoint breakthrough infections with an uncertainty of incidence and consequently rate of breakthrough infections during study follow-up. The sample size was calculated to detect an increase in the percentage of participants with neutralizing antibodies against the B.1.351 variant from 58% to 65.5% as statistically significant, with a power of >90% and an alpha error of 5%. If the increase is 10% the statistical power would be >99%. However, sample size calculation had the additional uncertainty that at the time point of writing of the protocol the clinically meaningful titer of neutralizing antibodies, i.e. the titer needed for protection, was not known. Immunogenicity and safety analysis were performed using a modified intention to treat population that includes all vaccinated participants, which completed at least one follow-up visit (N=108 for AZ/AZ, N=114 for AZ/BNT, N=30 for BNT/BNT, Figure 1). Safety analysis (secondary endpoint) was performed in a descriptive way showing relative frequencies at days 1-7 in bar charts and a table. Additionally, cumulative reactogenicity for each symptom (number of individuals with a given symptom at any time point within days 1-7) was analyzed using CIs of a proportion calculated by modified Wald method. Statistical analysis for primary endpoints was performed with non-parametric testing Kruskal-Wallis test with uncorrected Dunn's comparison. No correction for multiplicity was needed as only the two randomized groups (AZ/AZ and AZ/BNT) were compared and no within group comparisons were performed. Data were imported via CSV/excel files into IBM SPSS Statistics Version 26. Statistical analysis was performed using GraphPad Prism 9.0.1 or 9.2. Data plotted in linear scale were expressed as median ± standard deviation (SD). Data plotted in logarithmic scales were expressed as geometric mean ± geometric standard deviation (SD).

### Role of Funders

The Funders had no role in study design, data collection, data analyses, interpretation, or writing of report.

## Results

### Study participants

Participants were recruited in Tyrol, Austria, between May 11^th^ and July 10^th^, 2021. A total of 282 healthy volunteers, 18 to 65 years of age who already received the prime immunization, were screened and 264 received their second COVID-19 vaccination within the multicenter HEVACC trial with two randomized arms (AZ/AZ and AZ/BNT) and a third observational arm (BNT/BNT) (see Figure 1, Table 1, Supplementary Figure S1). The study participants had been vaccinated with AZ 11-13 weeks or BNT 3-6 weeks before the booster vaccination. Intervals have been selected according to recommendations by national authorities (Nationales Impfgremium Österreich, NIG) at the time point of study initiation for the respective homologous vaccination regiments, 4 weeks for BNT/BNT and 12 weeks for AZ/AZ. Individuals with previous SARS-CoV-2 infection as determined by a positive anti-SARS-CoV-2 N protein enzyme-linked immunosorbent assay (ELISA) during the screening visit one week before the intended boost, were excluded. Two homologous vaccination regimens with AZ (AZ/AZ, N=109) and BNT (BNT/BNT, N=30) were compared to the heterologous (AZ/BNT, N=115) combination. The two single-blinded groups primed with AZ were randomized to receive either an AZ or a BNT boost (Figure 1). Comparisons to the non-randomized BNT/BNT arm were exploratory. Reactogenicity within the first week after boost (Table 2, Supplementary Figure S3, and Supplementary Table S2) as well as laboratory blood parameters for toxicity on days 2 and 10 (Supplementary Figure S4) were determined. Immunogenicity was analyzed on days 10 and 30.

**Table 1:** Characteristics of the vaccinated participants at baseline before boost (ITT population).

Characteristic	AZ/AZ group N=109	AZ/BNT group N=115	BNT/BNT group N=30
Mean age ± SD [years] (95% CI)	37.78 ± 14.06 (35.11 - 40.45)	38.60 ± 12.74 (36.25 - 40.95)	33.47 ± 9.56 (29.90 - 37.04)
Number of females N (%)	59 (54.13)	62 (53.91)	16 (53.33)
Mean age females ± SD [years] (95% CI)	40.61 ± 14.18 (36.91 - 44.31)	40.73 ± 11.88 (37.71 - 43.74)	34.31 ± 11.16 (28.36 - 40.26)
Number of males N (%)	50 (45.87)	53 (46.09)	14 (46.67)
Mean age females ± SD [years] (95% CI)	34.44 ± 13.29 (30.66 - 38.22)	36.11 ± 13.36 (32.43 - 39.80)	32.50 ± 7.62 (28.10 - 36.90)
BMI* (mean ± SD)	23.55 ± 4.37	24.37 ± 4.58	24.05 ± 3.44
Underlying condition, N (%) Allergy Asthma Autoimmune Cardiovascular disease disease of the thyroid Gastroenterologic disorders metabolic disease Neoplasia renal disorder	17 (15,60) 0 (0,00) 6 (5,50) 5 (4,59) 13 (11,93) 0 (0,00) 4 (3,67) 0 (0,00) 1 (0,92)	20 (17,39) 2 (1,74) 12 (10,43) 14 (12,17) 19 (16,52) 2 (1,74) 4 (3,48) 1 (0,87) 0 (0,00)	5 (16,67) 0 (0,00) 2 (6,67) 1 (3,33) 4 (13,33) 0 (0,00) 0 (0,00) 0 (0,00) 0 (0,00)
Interval between 1^st^ and 2^nd^ dose (mean ± SD)§	82.30 ± 3.94	82.77 ± 4.50	32.37 ± 5.02
Baseline antibody titers, mean ± SD (95 % CI)$ Anti-S IgG Anti-S IgA	76.42 ± 114.4 (54.69 - 98.14) 0.561 ± 0.693 (0.430 - 0.693)	51.51 ± 46.65 (42.89 - 60.12) 0.754 ± 1.296 (0.514 - 0.993)	234.9 ± 183.2 (166.5 - 303.3) 2.839 ± 1.928 (2.119 - 3.559)
Number of participants per study center Innsbruck Kufstein Schwaz	58 10 41	59 11 45	30 0 0

⁎ Body-Mass-Index (weight/heigth²), kg/m².

§ days.

$ Anti-S IgG antibody titers are expressed in BAU/ml, values > 7.1 BAU/ml are considered positive, anti-S IgG antibody titers are expressed as optical density (OD), OD > 1.1 is considered positive.

**Table 2:** Cumulative reactogenicity in modified intention to treat population.§

		AZ/AZ (n = 108)		AZ/BNT (n = 114)		BNT/BNT (n = 30)	
		Number (proportion)*	95 % CI of proportion$	Number (proportion)*	95 % CI of proportion$	Number (proportion)*	95 % CI of proportion$
**Local**	Pain injection site	70 (0.6481)	0.5543 - 0.7319	96 (0.8421)	0.7633 - 0.8986	24 (0.8000)	0.6233 - 0.9086
	Erythema (redness) injection site	7 (0.0648)	0.0296 - 0.1300	16 (0.1404)	0.0872 - 0.2169	3 (0.1000)	0.0266 - 0.2642
	Swelling / tissue hardening injection site	16 (0.1481)	0.0923 - 0.2282	38 (0.3333)	0.2533 - 0.4242	5 (0.1667)	0.0686 - 0.3404
	Swelling or sensitivity axillary lymph node	3 (0.0278)	0.0060 - 0.0820	18 (0.1579)	0.1014 - 0.2367	7 (0.2333)	0.1152 - 0.4120
**Systemic**	Headache	43 (0.3981)	0.3108 - 0.4925	56 (0.4912)	0.4013 - 0.5818	18 (0.6000)	0.4229 - 0.7544
	Tiredness	53 (0.4907)	0.3984 - 0.5837	65 (0.5702)	0.4784 - 0.6573	24 (0.8000)	0.6233 - 0.9086
	Myalgia (muscle pain)	16 (0.1481)	0.0923 - 0.2282	31 (0.2719)	0.1984 - 0.3604	9 (0.3000)	0.1652 - 0.4802
	Arthralgia (joint pain)	11 (0.1019)	0.0563 - 0.1748	16 (0.1404)	0.0872 - 0.2169	6 (0.2000)	0.0914 - 0.3767
	Nausea / vomitus	3 (0.0278)	0.0060 - 0.0820	8 (0.0702)	0.0341 - 0.1343	2 (0.0667)	0.0080 - 0.2237
	Diarrhea	4 (0.0370)	0.0114 - 0.0944	8 (0.0702)	0.0341 - 0.1343	0 (0.0000)	0.0000 - 0.1347
	Increased body temperatura / fever	6 (0.0556)	0.0233 - 0.1184	17 (0.1491)	0.0943 - 0.2268	7 (0.2333)	0.1152 - 0.4120
	Chill	6 (0.0556)	0.0233 - 0.1184	8 (0.0702)	0.0341 - 0.1343	2 (0.0667)	0.0080 - 0.2237
	Rash / nettle rash	1 (0.0093)	<0.0001 - 0.0557	1 (0.0088)	<0.0001 - 0.0529	0 (0.0000)	0.0000 - 0.1347
	Itching	2 (0.0185)	0.0010 - 0.0691	3 (0.0263)	0.0056 - 0.0779	1 (0.0333)	<0.0001 - 0.1809
	Difficulty to breath	0 (0.0000)	0.0000 - 0.0413	2 (0.0175)	0.0009 - 0.0657	0 (0.0000)	0.0000 - 0.1347
	Other	19 (0.1759)	0.1148 - 0.2593	27 (0.2368)	0.1677 - 0.3231	7 (0.2333)	0.1152 - 0.4120

§ modified intention to treat population is defined as all vaccinated individuals who completed at least one follow-up visit after vaccination.

⁎ cumulative number and proportion of participants with respective symptom at any given time point between day 1-7 after second dose vaccination.

$ 95 % confidence intervals (CI) of proportion of participants with respective symptome at any given time point between day 1-7 after second dose vaccination by modified Wald method were calculated using GraphPad online tool (https://www.graphpad.com/quickcalcs/confInterval1/).

### Safety

In general, the vaccines were well tolerated with similar levels of reactogenicity in all three study groups (Table 2, Supplementary Figure S3, and Supplementary Table S2). In the heterologous group, slightly more participants reported symptoms such as pain, redness and swelling/tissue hardening at the injection site, elevated temperature/fever (self-reported), nausea/vomiting, myalgia, headache, and swelling of axillary lymph node compared to the homologous AZ/AZ group. However, these side effects were in a similar range as for the homologous BNT/BNT group. Comparisons for safety analysis were exploratory. No persisting symptoms were reported after 30 days. To further elaborate on potential toxicity of the heterologous AZ/BNT vaccination, blood parameters were analyzed for all participants on days 2 and 10. Values were similar in all groups (Supplementary Figure S4).

One serious adverse event was observed on day 7 in the heterologous group, where myocardial infarction occurred in a woman with preexisting severe coronary artery disease. However, this event was not considered to be related to the vaccination by the Data Safety Monitoring Board.

### Immunogenicity

Several assays were used to determine the antibody response (Figure 2). Anti-S IgG and IgA antibody levels were similar before the second vaccination in the two randomized study groups pre-vaccinated with AZ (median 49.6 binding antibody units (BAU)/ml (95% CI 45.3 - 62.4) and 37.8 BAU/ml (95% CI 32.3 - 47.6) for IgG; 0.379 (95% CI 0.316 - 0.451) and 0.360 (95% CI 0.325 - 1.296) for IgA). Antibody levels were higher in the BNT-primed group (median 225.6 BAU/ml (95%CI 136.3 - 258.2) for IgG; 2.387 (95% CI 1.625 - 3.301) for IgA). However, comparison of AZ- and BNT-primed groups is limited by the different intervals of boost immunization leading to a screening visit ∼9-11 weeks after prime for AZ-primed individuals and ∼2-5 weeks for BNT-primed individuals. In all groups, antibodies were found to be boosted by day 10 after the second vaccination with a small decline again up to day 30. The antibody levels after the boost vaccination were statistically significant higher in the heterologous AZ/BNT group than in the homologous AZ/AZ group (p<0.0001; median IgG titers for AZ/BNT 2538 BAU/ml (95% CI 2217 - 3045) on day 10 and 1478 BAU/ml (95% CI 1234 - 1826) on day 30 versus 245.6 BAU/ml (95% CI 194.0 - 284.9) on day 10 and 197.8 BAU/ml (95% CI 161.7 - 227.0) on day 30 for AZ/AZ), while the heterologous group reached similar levels as the homologous BNT/BNT group (median IgG titers for BNT/BNT 3385 BAU/ml (95% CI 2399 - 4255) on day 10 and 2011 BAU/ml (95% CI 1298 - 3913) on day 30, see Supplementary Table S3 for an overview of median binding antibody titers and 95 % CI for all groups and time points). Similar kinetics were seen for the nAb titers as determined with the vesicular stomatitis virus-based pseudovirus neutralization assay against ancestral (wild type) S. NAbs were boosted in all three groups. However, titers were statistically significantly higher in the heterologous AZ/BNT group compared to the homologous AZ/AZ regimen (p<0.0001 for comparison of AZ/AZ with AZ/BNT both on day 10 and on day 30, with median reciprocal nab titers for AZ/BNT of 670.4 (99.9 % CI 506.2 - 896.7) on day 10 and 464.0 (348.2 - 612.5) compared to median reciprocal nab titers for AZ/AZ of 73.8 (55.2 - 97.0) on day 10 and 58.1 (44.7 - 74.9) on day 30). Nab titers were similar for AZ/BNT and BNT/BNT vaccinated individuals (Supplementary Table S4). In addition, we tested the nAb titers in a focus-forming unit assay using live SARS-CoV-2 variants B.1.1.7, B.1.351, and B.1.617.2 (Figure 2d, Supplementary Figure S4). Comparable to previous reports for vaccine induced immunity,^21^, ^22^, ^23^, ^24^ nAb responses within each of the three study groups were lower against B.1.351 and B.1.617.2 than against the B.1.1.7 variant. For all three variants, absolute nAb titers were statistically significant lower after homologous AZ/AZ immunization than in the AZ/BNT groups (see Supplementary Table S4 for median and 99.9 % CI antibody titers for all groups against the different variants, Supplementary Table S5 for effect size for comparison of the two randomized arms, and Supplementary Table S9 for p values for the comparisons between the two randomized arms).

**Figure 3:**
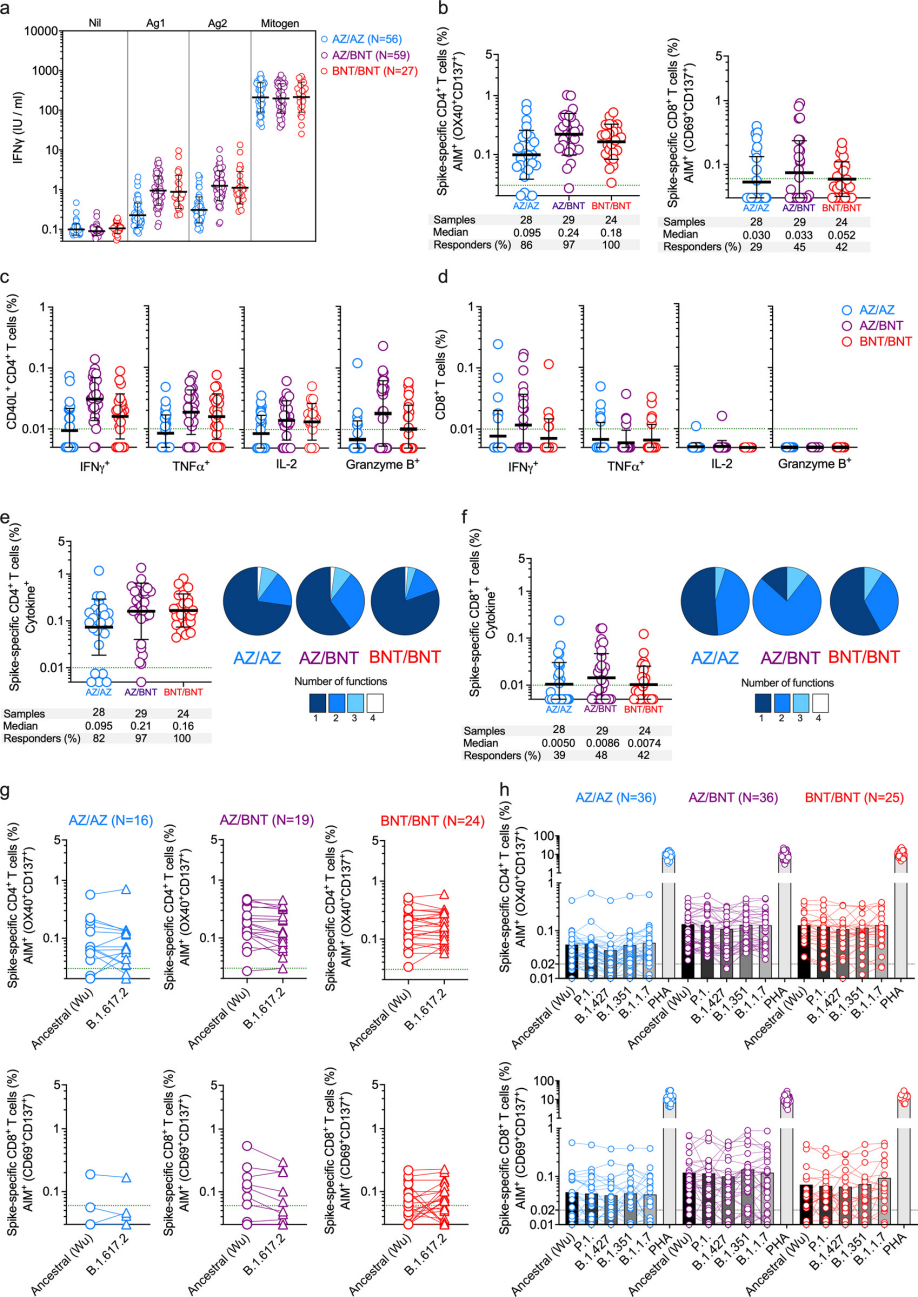
**Spike specific T cell responses are higher after heterologous vaccination.** A. Quantiferon IFNγ release assay measuring IFNγ (IU/ml) in blood samples from AZ/AZ (blue circles), AZ/BNT (purple circles), BNT/BNT (red cicrles) vaccine groups at 10 days after boost vaccination. Whole blood samples were either non-stimulated (Nil) or stimulated with specific CD4 (Ag1) and CD4+CD8 (Ag2) SARS-CoV-2 peptide pools from spike antigen (S1 S2 RDB). As positive control mitogen-stimulated samples were also analyzed. B. spike-specific CD4^+^ T cells and CD8^+^ T cells were quantified by AIM (surface OX40^+^CD137^+^ and CD69^+^CD137^+^, respectively). Comparison of spike-specific AIM^+^ CD4^+^ T cell and CD8^+^ T frequencies between AZ/AZ (blue circles), AZ/BNT (purple circles), and BNT/BNT (red circles) vaccine regimens at day 10 post boost are shown. The bars indicate the geometric mean and geometric SD in the analysis of the spike-specific CD4^+^ and CD8^+^ T cell frequencies. C. spike-specific CD4^+^ T cells expressing intracellular CD40L (iCD40L) and producing IFNγ, TNFα, IL-2 or granzyme B (GzB) and spike-specific CD8^+^ T cells producing IFNγ, TNFα, IL-2 or GzB by intracellular cytokine staining (ICS) (D). The dotted green line indicates limit of quantification (LOQ). Proportions of multifunctional activity profiles of the spike-specific CD4^+^ (E) and CD8^+^ T cells (F) evaluated on days 10 post boost. The dark blue, navy blue, turquoise and white colors in the pie charts depict the production of one, two, three, and four functions, respectively. spike specific AIM^+^ CD4 and CD8 T cells against the ancestral spike sequence were compared to the spike sequence derived from the B.1.617.2 (G) as well as the P.1., the B.1.427/B.1.429, the B.1.351 and the B.1.1.7 (H) variants at day 10 post boost. Background-subtracted and log data analyzed in all cases. N as indicated in figure. 95 % confidence intervals are shown in Supplementary Tables S6-S8.

We then investigated SARS-CoV-2-specific cellular responses. All comparisons with the non-randomized BNT/BNT arm were exploratory. Ten days post boost vaccination, we found for all groups a production of IFN-γ in response to SARS-CoV-2-derived CD4 (Ag1) and CD4/CD8 (Ag2) peptide pools in the QFN IGRA assay relative to the non-stimulated controls (Figure 3A, Supplementary Table S6). Importantly, heterologous vaccination resulted in a statistically significant higher production of IFN-γ against both Ag1 (geometric mean 1.34 international units (IU)/ml (95% CI 1.03-1.65) versus 0.31 IU/ml (95% CI 0.21-0.41)) and Ag2 (geometric mean 1.78 IU/ml (95% CI 1.33-2.22) versus 0.43 IU/ml (95% CI 0.30-0.56)) relative to AZ/AZ vaccination. The heterologous group reached levels similar to the homologous BNT group (geometric mean 1.51 IU/ml (95% CI 0.67-2.35) for Ag1 and 1.94 IU/ml (95% CI 0.79-3.08) for Ag2 for BNT/BNT). IFN-γ response to SARS-CoV-2 had slightly declined 30 days post boost. However, IFN-γ levels were still statistically significant higher in the heterologous group for both Ag1 (geometric mean 0.68 IU/ml (95% CI 0.52-0.84) for AZ/BNT versus 0.22 IU/ml (95% CI 0.17-0.27) for AZ/AZ) and Ag2 (geometric mean 0.94 IU/ml (95% CI 0.67-1.21) for AZ/BNT versus 0.30 IU/ml (95% CI 0.22-0.39) for AZ/AZ) (Supplementary Figure S6, Supplementary Table S6).

To determine antigen-specific responding cellular subsets, spike-specific T cell responses were also studied in the AIM assay by measuring the surface expression of activation-induced cell markers CD134 (OX40) and CD137 on CD4^+^, and CD69 and CD137 on CD8^+^ T cells (Supplementary Figure S2). In line with the IGRA results, levels of spike-specific AIM^+^ CD4^+^ T cells were statistically significant higher in the heterologous group compared to homologous AZ/AZ vaccination (Figure 3B, geometric mean 0.30 (95% CI 0.20-0.39) versus 0.15 (95% CI 0.089-0.22)). However, lower differences in the AIM^+^ CD8^+^ T cell frequencies were found (Figure 3B and Supplementary Table S7, geometric mean 0.16 (95% CI 0.069-0.25) versus 0.088 (95% CI 0.047-0.13)). Importantly, statistically significant higher frequencies of CD4^+^ T cells producing IFNγ, TNFα, and GzB in response to spike-peptide pool stimulation were detected in the heterologous group compared to the homologous AZ vaccination (Figure 3C and Supplementary Table S8). However, no marked differences in the production of IL-4 and IL-17 by CD4^+^ T cells could be detected (Supplementary Figure S2). Notably, we also found increased proportions of multifunctional activity profiles of spike-specific CD4^+^ in the heterologous group (Figure 3E). Of note, we found no difference in the levels of spike-specific circulating T follicular helper cells (cT_FH_) cells between heterologous AZ/BNT and homologous AZ/AZ groups (Supplementary Figure S2). In contrast to CD4^+^ T cells, heterologous AZ/BNT and homologous AZ/AZ vaccination regimens induced a similar magnitude and quality of spike-specific CD8^+^ T cells (Figure 3D and 3F).

Taken together, all three different assays for T cell reactivity to SARS-CoV-2 S protein showed a higher number and functionality of T cells induced by heterologous AZ/BNT and homologous BNT/BNT regimen relative to the homologous AZ/AZ group.

T cell responses against SARS-CoV-2 VoCs utilizing variant-specific peptide pools were studied by AIM assay. First, we analyzed the B.1.617.2 variant and found no differences in AIM^+^ CD4^+^ and CD8^+^ T cell frequencies compared to the ancestral virus variant (Figure 3G and Supplementary Table S7). Next, we extended these experiments to a set of other variants including peptide pools derived from P.1, B.1.427/B.1.429, B.1.351 and B.1.1.7 (Figure 3H). We found statistically significant higher frequencies of AIM^+^ CD4^+^ T cells in the heterologous AZ/BNT group in response to all peptide pools when compared to the homologous AZ/AZ regimen at 10 days post boost (Figure 3H and Supplementary Table S7 for 95 % CI in Supplementary Appendix). However, only slightly elevated frequencies of AIM^+^ (CD69^+^CD137^+^) CD8^+^ T cells were found in PBMCs at 10 days post boost vaccination in the heterologous group if stimulated with ancestral Wuhan-1 and variant pools compared to the homologous AZ group. At 30 days post vaccination, only minor differences between the AZ/AZ and AZ/BNT groups both in AIM^+^ CD4^+^ nor in CD8^+^ T cells were observable (Supplementary Figure S6 and Supplementary Table S7). Interestingly, compared to ancestral wild type peptide pool we found lower frequencies of AIM^+^ CD4^+^ T cells in response to B.1.427/B.1.429 variant pool in all vaccine groups at day 10 but not at day 30 post boost vaccination (Figure 3H and Supplementary Figure S6). Importantly, both, antibody response and IFNγ release were comparable between women and men (Supplementary Figure S7).

## Discussion

In the HEVACC trial, we found that the heterologous vaccination with an AZ prime followed by a BNT boost induced higher antibody and T cell responses to SARS-CoV-2 than the homologous AZ vaccination. The immune response to the heterologous schedule was similar to the response induced by homologous BNT vaccination.

In general, the vaccines were well tolerated. This is in line with the CombiVacS study reporting only mild to moderate site effects after heterologous AZ/BNT vaccination.^25^ However, this study did not include homologous AZ/AZ or BNT/BNT control groups. In contrast to a previous publication from the Com-COV study,^26^ the heterologous vaccination did not cause a pronounced higher reactogenicity. The reason could be the longer period between prime and boost, which was 28 days in the Com-COV study and 8-12 weeks in the study presented here and the CombiVacS study.

Our study is in line with very recent studies but adds additional crucial data. In the first study, the prospective, observational CoCo study, heterologous vaccination with AZ followed by BNT was also found to induce a higher level of antibody and T cell responses than homologous AZ/AZ vaccination in healthcare professionals. Our study extends the data to larger study groups randomized for the boost vaccination stratified by sex. In addition, we analyzed nAbs to the B.1.1.7, B.1.351 and B.1.617.2 variants using live virus variants instead of using surrogate neutralization assays and the T cell responses in more detail and to a broad range of different SARS-CoV-2 variants.^27^^,^^28^ Also in other prospective or retrospective observational cohort studies enrolling primarily health-care workers, the heterologous combination AZ/BNT was found to be as or even more immunogenic than the homologous regimen. However, in addition to randomization of the two AZ-primed arms, our study includes a far more detailed analysis of the T cell and antibody responses.^29^, ^30^, ^31^, ^32^ In our study, there was no difference in the antibody or T-cell response between the heterologous AZ/BNT and homologous BNT/BNT arm, which is in line with the randomized Com-COV study results on immunogenicity also published recently.^33^ While the Com-COV study used a 28-day prime-boost interval in all groups our study used a longer interval between prime and boost for the two randomized AZ-primed arms as recommended by national authorities in Austria and by the WHO. An important complementation of our study to the Com-COV study is the younger study population in our study (18-65 years) compared to 50-70 year old participants in the Com-COV study.

Antibody responses, including nAb response to the immune escape variants B.1.351 and B.1.617.2, were boosted to statistically significant lower levels in the homologous AZ/AZ groups relative to the AZ/BNT group. Although, the nAb responses to these variants were lower than to the B.1.1.7 variant within each study group, the responses in the heterologous AZ/BNT and homologous BNT/BNT groups to B.1.351 and B.1.617.2 were higher than in the homologous AZ/AZ group to B.1.1.7. As there most likely is a correlation between antibody titer and protection, this finding would indicate that the protection of the AZ/AZ vaccine against B.1.1.7 is lower than of the other two vaccination regimens against the immune escape variants.^3^

A higher magnitude and multifunctionality of spike specific T cell responses was found in the homologous BNT/BNT and heterologous AZ/BNT regimen relative to the homologous AZ/AZ vaccination. Importantly, spike-specific T cells induced by either vaccination schedule were able to mount a comparable response against all VoC tested, including B.1.617.2. This confirms previous data that immune escape is generally not seen for the T-cell response.^34^

One limitation of the study is the relative short follow-up. A recent study has shown that the immunity after BNT/BNT vaccination declines over time in the first six months.^35^ Here, it will be of major interest to see how stable the immunity will be in the different study groups during follow-up. Another limitation is the different interval between prime and boost, 11-13 weeks for AZ/AZ and AZ/BNT and 3-6 weeks for BNT/BNT. This could cause differences in immunogenicity.

A second limitation of the study is that patient recruitment for the AZ/AZ arm was stopped early after interim analysis according to the Haybittle Peto rule as neutralizing antibodies (primary endpoint) were clearly inferior for this group compared to the second randomized arm (AZ/BNT). Therefore, the total number of participants in the AZ/AZ arm regarding the secondary endpoint breakthrough infections is limited and the study might consequently not have enough power to analyze this secondary endpoint. However, at the time point of the interim analysis also others reported a superiority of the heterologous AZ/BNT regiment, which might have complicated further patient recruitment.

A third limitation of our study is that nAb and T cell responses were only analyzed against pre-omicron variants but not against the currently circulating B.1.1.529 subvariants. However, we and others showed earlier that nAb against B.1.1.529 were low and only short lived after two vaccine doses. However, a third dose of vaccination or hybrid immunity strongly enhanced titers against B.1.1.529.^6^^,^^8^^,^^36^ In contrast, T cell responses are more conserved between pre-omicron variants and B.1.1.529.^37^^,^^38^

The results of this study clearly support heterologous prime/boost vaccination with AZ followed by BNT and encourage similar studies for other heterologous vector vaccine combinations against COVID 19.

## Contributors

ZB and JM performed the T cell response assays; AR and LR performed the antibody assays; AG performed all the bioinformatic analyses related to the variant pool generation and design under AS supervision; VS contributed reagents; HS, DB, BF, WB did the clinical work; HU was responsible for the statistics; BN managed the study; DW (T cells), FK, DvL (PI), JK (antibodies) designed, supervised and coordinated the study and wrote the manuscript. ZB, JM, DW (T cells) and AR, DvL and JK (safety and antibodies) had access to the whole data set and verified the underlying data. All authors edited and reviewed the manuscript prior to submission. All authors read and approved the final version of the manuscript.

## Declaration of interests

The Icahn School of Medicine at Mount Sinai has filed patent applications relating to SARS-CoV-2 serological assays and NDV-based SARS-CoV-2 vaccines which list Florian Krammer as co-inventor. Viviana Simon is also listed on the serological assay patent application as co-inventor. Mount Sinai has spun out a company, Kantaro, to market serological tests for SARS-CoV-2. Florian Krammer has consulted for Merck and Pfizer (before 2020), and is currently consulting for Pfizer, Seqirus and Avimex. The Krammer laboratory is also collaborating with Pfizer on animal models of SARS-CoV-2. A.S. is a consultant for Gritstone, Flow Pharma, Arcturus, Immunoscape, CellCarta, OxfordImmunotech and Avalia. LJI has filed for patent protection for various aspects of T cell epitope and vaccine design work. Dorothee von Laer received fundings from the Medical University of Innsbruck. All other authors declare no conflict of interest.

## Appendix Supplementary materials

Image, application 1

Study Protocol. Image, application 2
