# Supplementary material for: Heterologous ChAdOx1/BNT162b2 vaccination induces stronger immune response than homologous ChAdOx1 vaccination: The pragmatic, multi-center, three-arm, partially randomized HEVACC trial
Source: eBioMedicine. 2022 May 23;80:104073. doi: 10.1016/j.ebiom.2022.104073 (PMC9126042; doi:10.1016/j.ebiom.2022.104073)
Supplement: Supplementary file 1 [file mmc1.pdf]

Supplementary Appendix

HEVACC Study Group

Petra Flatscher[a], Lukas Forer[b], Elisabeth Graf[c], Gerhard Hausberger[c], Peter Heininger[a], Michael Kundi[d], Christine Mantinger[a], Conny Ower[e], Daniel Rainer[a], Magdalena Sacher[f], Lisa Seekircher[g], Sebastian Schönherr[b], Marton Szell[h], Tobias Trips[c], Ursula Wiedermann[i], Peter Willeit[g,j], Reinhard Würzner[j], August Zabernigg[c]

[a] Hospital Schwaz, Schwaz, Austria

[b] Institute of Genetic Epidemiology, Medical University of Innsbruck, Austria

[c] Hospital Kufstein, Kufstein, Austria

[d] Center for Public Health, Department of Environmental Health, Medical University of Vienna, 1090 Vienna, Austria

[e] Hospital Innsbruck, Innsbruck, Austria

[f] Department of Visceral, Transplant and Thoracic Surgery, Center of Operative Medicine, Medical University of Innsbruck, Innsbruck, Austria

[g] Clinical Epidemiology Team, Department of Neurology, Medical University of Innsbruck, Innsbruck, Austria

[h] Emergency Department, Klinik Donaustadt, Langobardenstrasse 122, 1220, Vienna, Austria

[i] Institute of Specific Prophylaxis and Tropical Medicine, Center of Pathophysiology, Infectiology and Immunology, Medical University of Vienna, Vienna, Austria

[j] Department of Public Health and Primary Care, University of Cambridge, United Kingdom

[k] Department of Hygiene, Microbiology and Public Health, Medical University of Innsbruck, Innsbruck, Austria

Supplementary Methods

Antibody testing

Anti-N antibodies were determined using the Elecsys Anti-SARS-CoV-2 (Roche Diagnostics, Indianapolis, USA) according to manufacturer's recommendations. A cutoff index (COI), which is calculated using the standards provided by the manufacturer ≥1.0 was considered positive. Anti-S IgA was quantified using a commercially available anti-SARS-CoV-2-IgA ELISA (Euroimmun, Lübeck, Germany) using the fully automated 4-plate benchtop instrument Immunomat™ (Virion/Serion, Würzburg, Germany). Optical density (OD) values were interpreted according to the recommendations in the manufacturer's information and values >1.1 were considered positive. Borderline values (0.8-1.1) were considered negative. Anti-S IgG was quantified using the Architect anti-SARS-CoV-2 IgG II Quant assay from Abbott and an Alinity Ci-series device. Results from the kit in AU/ml are calculated to the WHO unit binding antibody units (BAU)/ml by multiplication with 0.142. Values >7.1 BAU/ml were considered positive. SARS-CoV-2 neutralizing antibodies were determined as described previously using a vesicular stomatitis virus (VSV) pseudovirus assay or a focus forming assay with replication competent SARS-CoV-2.[1] For the VSV pseudovirus assay, neutralizing antibody titers against

the ancestral Whuan-1 S protein were determined. For the focus forming assay, titers against B.1.1.7 (isolate C69.1, GISAID ID EPI_ISL_3277382), B.1.351 (isolate C24.1, GISAID ID EPI_ISL_1123262) and B.1.617.2 (isolate SARS-CoV-2-hCoV-19/USA/NY-MSHSPSP-PV29995/2021(B.1.617.2)) were determined. Continuous 50% neutralization titers were calculated using a nonlinear regression as described earlier.[2] Titers >1:16 were considered as positive. Values >1:1024 were set to 1:1024 and values <1:16 to 1:16.

T cell assays

Interferon-γ release assay (IGRA). SARS-CoV-2-specific interferon-γ release from whole blood cells was measured by QuantiFERON (QFN) SARS-CoV-2 RUO IGRA (Qiagen) according to manufacturer's instructions.

Mutation analysis of SARS-CoV-2 B.1.1.7, B.1.351, P.1, B.1.427 and B.1.617.2 variants. Genome sequences for the variant viruses were downloaded from GISAID. These sequences were screened to select those without ambiguous residues and generated from Illumina sequencing technologies using an in-house sequence QC script. The selected genomic sequences were then translated into protein amino acid sequences using the VIGOR4 tool available on the Virus PathogenResource (ViPR) website (https://www.viprbrc.org/). Sequence variations in the variant viruses were derived by comparison with Wuhan-1 (NC_045512.2). One or more representative sequences were considered for the B.1.1.7 (EPI_ISL_601443), P.1 (EPI_ISL_804823), B.1.427/B.1.429 (EPI_ISL_847619; EPI_ISL_847621; EPI_ISL_847643) and B.1.351 (EPI_ISL_660629; EPI_ISL_736930; EPI_ISL_736932; EPI_ISL_736944; EPI_ISL_736966; EPI_ISL_736971; EPI_ISL_736973; EPI_ISL_825104; EPI_ISL_825120; EPI_ISL_825131) variants. A summary of all the amino acids mutated in the different variants in respect to the ancestral Wuhan-1 sequence has been published before.[3]

Peptide megapools (MP). We have previously developed an approach that allows simultaneous testing of a large number of epitopes in small amounts of blood.[4] According to this so called Megapool (MP) approach, large numbers of different epitopes are solubilized, pooled, and re-lyophilized to avoid cell toxicity problems associated with high concentrations of DMSO typically encountered when single pre-solubilized epitopes are pooled.[4-7] To capture spike-specific responses, we utilized a MP of 253 overlapping peptides corresponding to the entire lengths of the spike protein of either the ancestral of variant sequences. As this peptide pool consists of peptides with a length of 15 amino acids, both $CD4^+$ and $CD8^+$ T cells have the capacity to recognize this MP.[8] We have previously shown that these MPs are suitable to stimulate T cell responses from either COVID-19 exposed or SARS-CoV-2-vaccinated individuals.[6,9]

Isolation of peripheral blood mononuclear cells (PBMCs). Whole blood was collected in S-Monovette® EDTA tubes (Sarstedt). PBMC were separated using Leucosep™ tubes (Greiner Bio-One) and then cryopreserved in cell recovery media containing 10% DMSO (Sigma) and 90% heat inactivated fetal bovine serum (FBS, Gibco), and stored in liquid nitrogen until used in the assays.

Activation induced markers (AIM) assay. Antigen-specific CD4+ T cells were measured as a percentage of AIM^+ ($OX40^+CD137^+$) $CD4^+$ and ($CD69^+CD137^+$) $CD8^+$ T cells after stimulation of

PBMCs with peptide MPs as previously described.[5,10] Antigen-specific circulating T follicular helper (cT_{FH}) cells were defined as percentage of CD4$^+$ T cells co-expressing CXCR5$^+$OX40$^+$CD40L$^+$. Prior to addition of MPs, cells were blocked at 37°C for 15 min with 0.5 µg/ml anti-CD40 mAb (Miltenyi Biotec). Then, cells were incubated at 37°C for 24 hours in the presence of fluorescently labeled chemokine receptor antibodies (anti-CCR6, CXCR5, CXCR3, CCR7, and CCR4) and SARS-CoV-2 MPs (1 µg/ml) or an equimolar amount of DMSO as negative control and with phytohemagglutinin (5 µg/ml) (PHA, Roche) as a positive control. For the surface stain, 1×10^6 PBMCs were reincubated with BD human FC block (BD Biosciences, San Diego, CA) and the LIVE/DEAD marker in the dark for 15 min and washed with PBS. Then, antibody mix containing the rest of the surface antibodies were added directly to cells and incubated for 60 min at 4°C in the dark. All samples were acquired on a Cytek Aurora (Cytek Biosciences, Fremont, CA). A list of antibodies used in this panel can be found in Supplementary Table 2 and a representative gating strategy of spike-specific CD4$^+$ and CD8$^+$ T cells using the AIM assay is shown in Supplementary Figure 2. Antigen-specific CD4$^+$ and CD8$^+$ T cells were measured as background (DMSO) subtracted data, with a minimal DMSO level set to 0.005%. Response > 0.02% and a stimulation index (SI) > 2 for CD4$^+$ and > 0.03% and SI > 3 for CD8$^+$ T cells were considered positive. The limit of quantification (LOQ) for antigen-specific CD4$^+$ T cell responses (0.03%) and antigen-specific CD8$^+$ T cell responses (0.06%) was calculated using the median two-fold standard deviation of all negative controls.

Intracellular cytokine staining (ICS) assay. To detect cytokine specific T cell responses PBMCs were cultured in the presence of SARS-CoV-2 MPs (1 µg/ml) for 24 hours at 37°C as previously described.[5] DMSO was used as negative control. After 24 hours, Golgi-Plug and Golgi-Stop were added to the culture for 4 hours. Cells were then washed and surface stained for 30 min at 4°C in the dark and fixed with 1% of paraformaldehyde (Sigma-Aldrich, St. Louis, MO). Antibodies used in the ICS assay are listed in Supplementary Table 2 and a representative gating strategy of spike-specific CD4$^+$ and CD8$^+$ T cells using the ICS assay is shown in Supplementary Figure 2. Antigen-specific CD4$^+$ and CD8$^+$ T cells were measured as background (DMSO) subtracted data, with a minimal DMSO level set to 0.001%. Responses >0.005% and a SI>2 for CD4+ and CD8$^+$ T cells were considered positive. The limit of quantification for antigen-specific CD4$^+$ and CD8$^+$ T cell responses (0.01%) was calculated using the median two-fold standard deviation of all negative controls. To define the multifunctional profiles of antigen-specific T cells, all positive background-subtracted data (> 0.005% and a SI > 2 for CD4+ T cells and > 0.002% and a SI > 2 for CD8+ T cells) was aggregated into a combined sum of antigen-specific CD4$^+$ or CD8$^+$ T cells based on the number of functions. Values higher than the LOQ (0.01%) were considered for the analysis of the multifunctional antigen-specific T cell responses. The average of the relative CD4$^+$ and CD8$^+$ T cell response was calculated per group to define the proportion of multifunctional antigen-specific T cell responses.

AIM assay to detect T cell responses against variants. PBMCs (1×10^6) were cultivated in the presence of 1 µg/ml SARS-CoV-2-derived peptide pools (ancestral Wuhan and variants) in 96-well U-bottom plates. A negative control with an equimolar amount of DMSO as well as a positive control containing 5 µg/ml phytohemagglutinin (PHA, Sigma) were also used. After 24 hours incubation at 37°C, 5% CO_2, PBMCs were stained with antibodies listed in Supplementary Table 2. Samples were measured on a BD FACSCanto™ II flow cytometer (BD Bioscience) and data were analyzed with FlowJo™ software (version 10, BD Bioscience).

Supplementary Figures

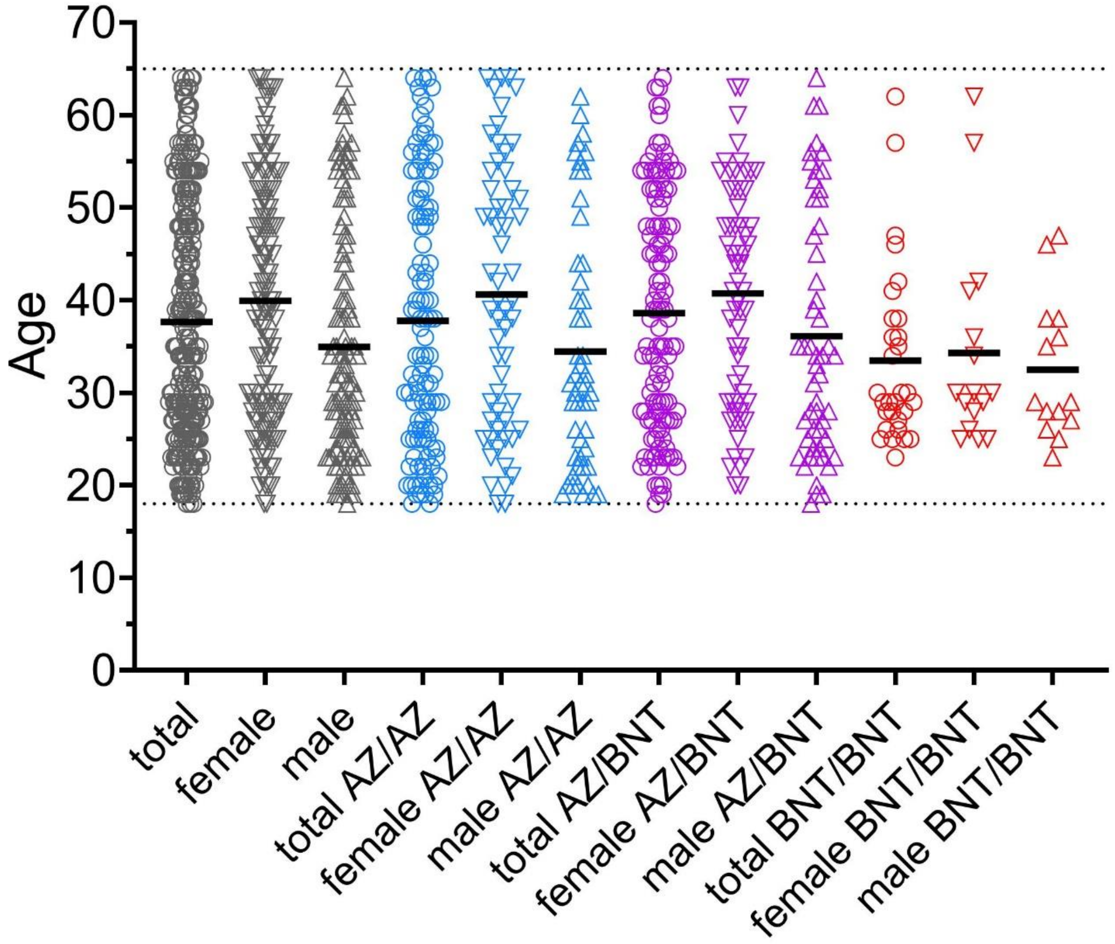

Supplementary Figure S1. Age distribution of participants. Participants between 18 and 65 (dotted lines) were included into the study. Shown are mean and individual values for total, female and male participants in each group. N, mean ± SD and 95% CIs are shown additionally in Table 1.

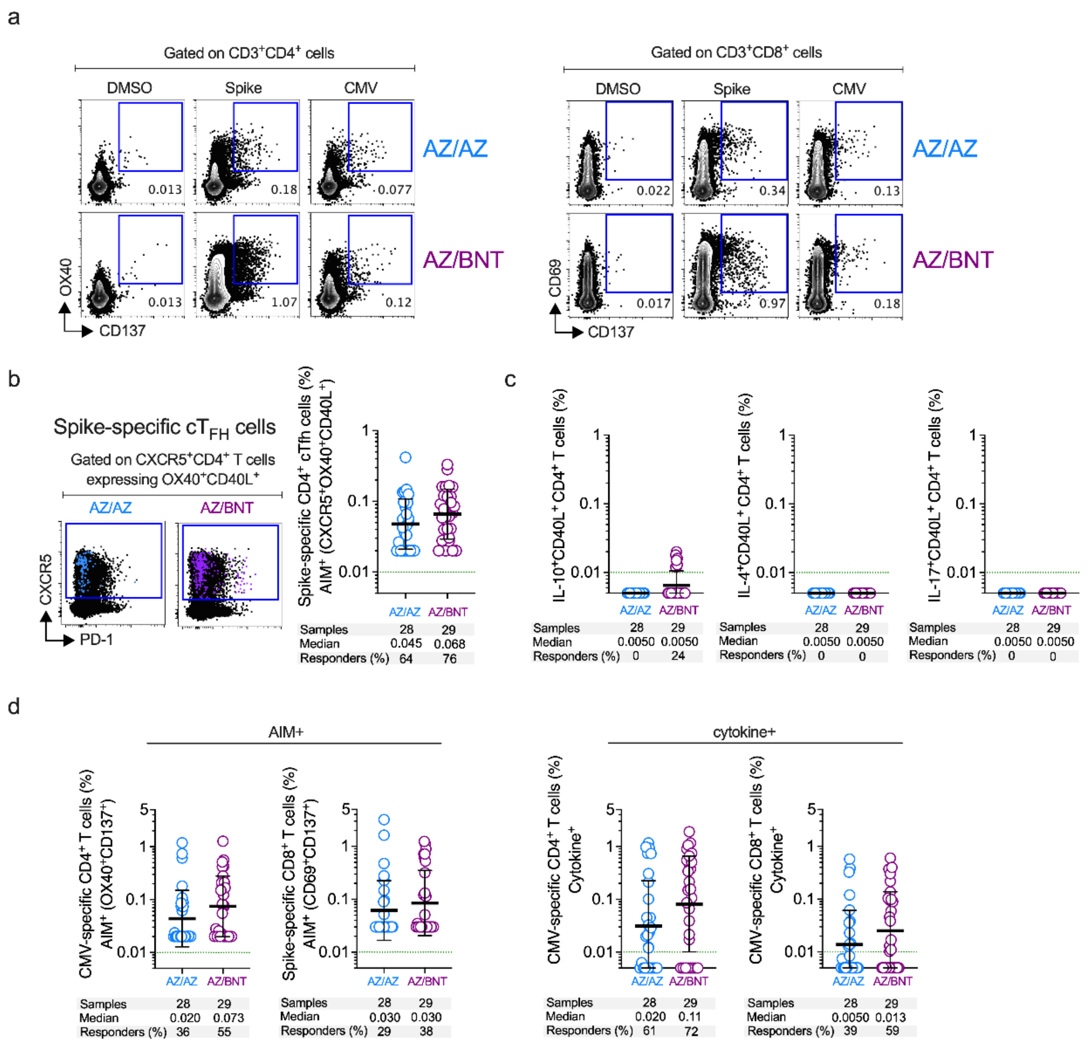

Supplementary Figure S2. T cell responses on day 10 post boost. A. Representative strategies to define CD3⁺CD4⁺ and CD3⁺CD8⁺ T cells by AIM assay. B. Representative examples of Spike-specific circulating T follicular helper (cT_{FH}) cells (blue = AZ/AZ; purple = AZ/BNT), overlaid on total $CD4^+$ T cells. Quantitation of Spike-specific cT_{FH} cells ($CXCR5^+OX40^+$surface $CD40L^+$, as percentage of $CD4^+$ T cells) after stimulation with Spike MP. C. Spike-specific $CD4^+CD40L^+$ T cells producing IL-4, IL-10, and IL-17 in vaccinees. No IL-4- or IL-17-producing $CD4^+$ T cells in response to Spike MP were detected at any time-point post immunization. Spike-specific IL-10-producing $CD4^+$ T cell response was detected in 24% of the subjects that received the heterologous vaccine. The dotted green line indicates limit of quantification (LOQ). The bars indicate the geometric mean and geometric SD in the analysis of the Spike-specific $CD4^+$ T cell frequencies. Background-subtracted and log data analyzed in all cases. D. Percentage of background subtracted CMV-specific $CD4^+$ and $CD8^+$ T cells quantified by AIM ($OX40^+CD137^+$ and $CD69^+CD137^+$, respectively) after stimulation with CMV MP. Percentage of background subtracted CMV-specific $CD4^+$ and $CD8^+$ T cells quantified by ICS after stimulation with the CMV MP. A Boolean gating strategy was used to define the frequencies of $CD8^+$ T cells producing IFNγ, TNFα, IL-2 or GzB. The dotted green line indicates limit of quantification (LOQ). The bars indicate the geometric mean and geometric SD in the analysis of the CMV-specific $CD8^+$ T cell frequencies. The bottom panels show the samples included in each group, the median of the frequencies, and the percentage of responders. Background-subtracted and log data analyzed in all cases.

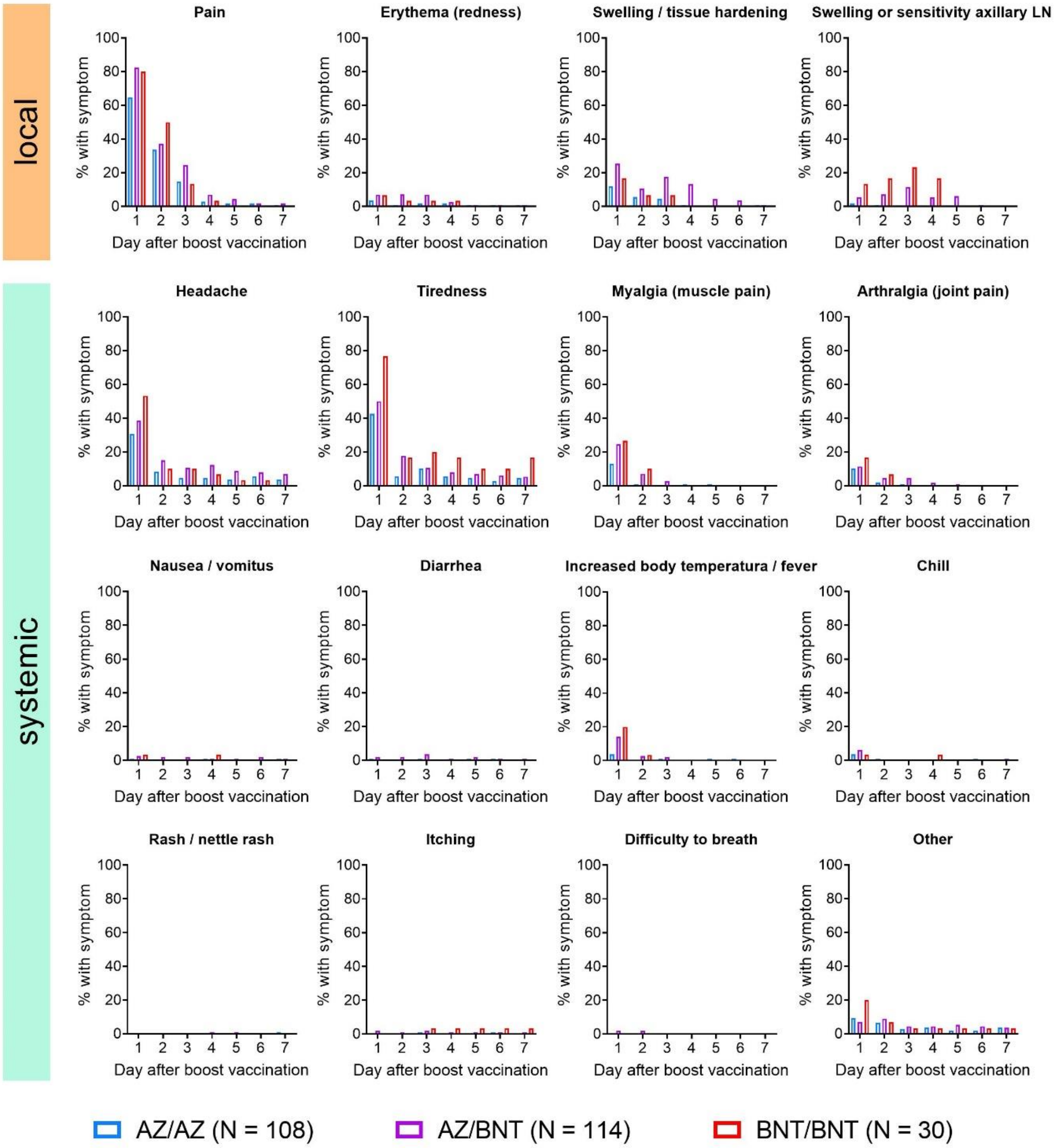

Supplementary Figure S3. Self-reported daily side effects after boost vaccination for modified intention to treat population. Shown is the percentage of participants, which reported the respective symptom after boost vaccination (days 1-7). One participant for the AZ/AZ and one for the AZ/BNT group dropped out of the study directly after the vaccination. Therefore, the number of participants followed for side effects was N=108 for AZ/AZ and N=114 for AZ/BNT (modified intention to treat population).

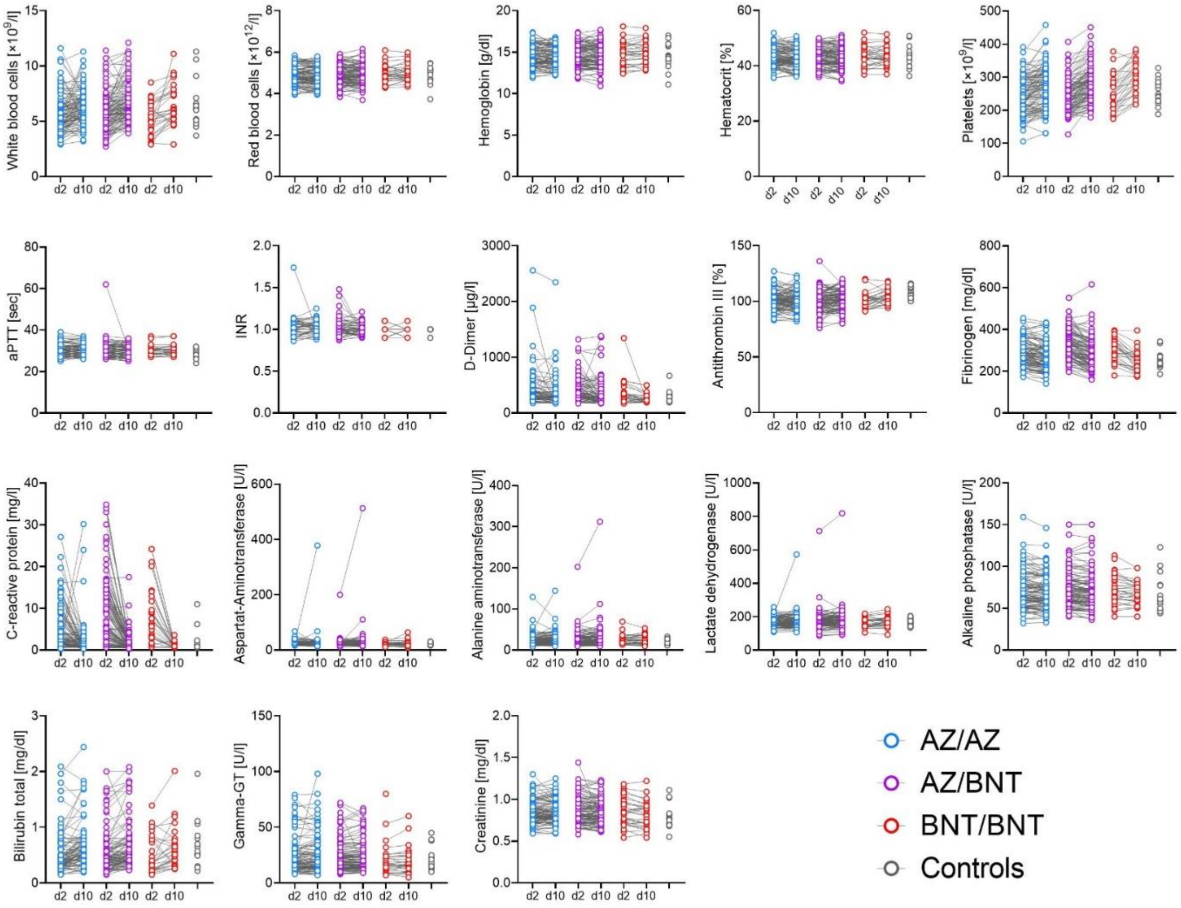

Supplementary Figure S4. Blood parameters day 2 and day 10 post boost vaccination. Blood was drawn on day 2 and day 10 post vaccination and blood values were determined. As control, blood was drawn from 17 healthy controls, which were at least 6 weeks post vaccination.

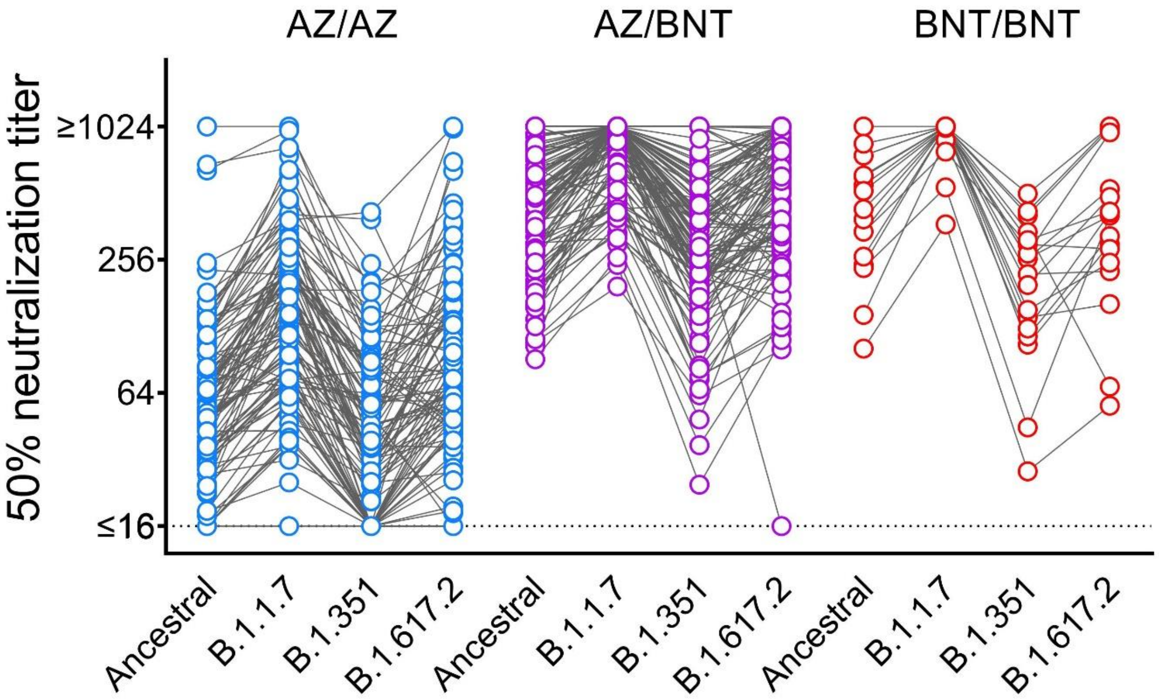

Supplementary Figure S5. Antibody responses are higher after heterologous vaccination compared to homologous AZ vaccination. 50% neutralization titers as determined in a VSV pseudovirus neutralization assay using ancestral (wild type) spike or focus forming assay using replication competent B.1.1.7, B.1.351 and B.1617.2 isolates. Individual values are shown. Samples from the same patient analyzed for the different variants are connected with lines. Titers ≤ 1:16 were considered negative (indicated by the dotted line). Values <1:16 were set to 1:16 and values >1:1024 to 1:1024.

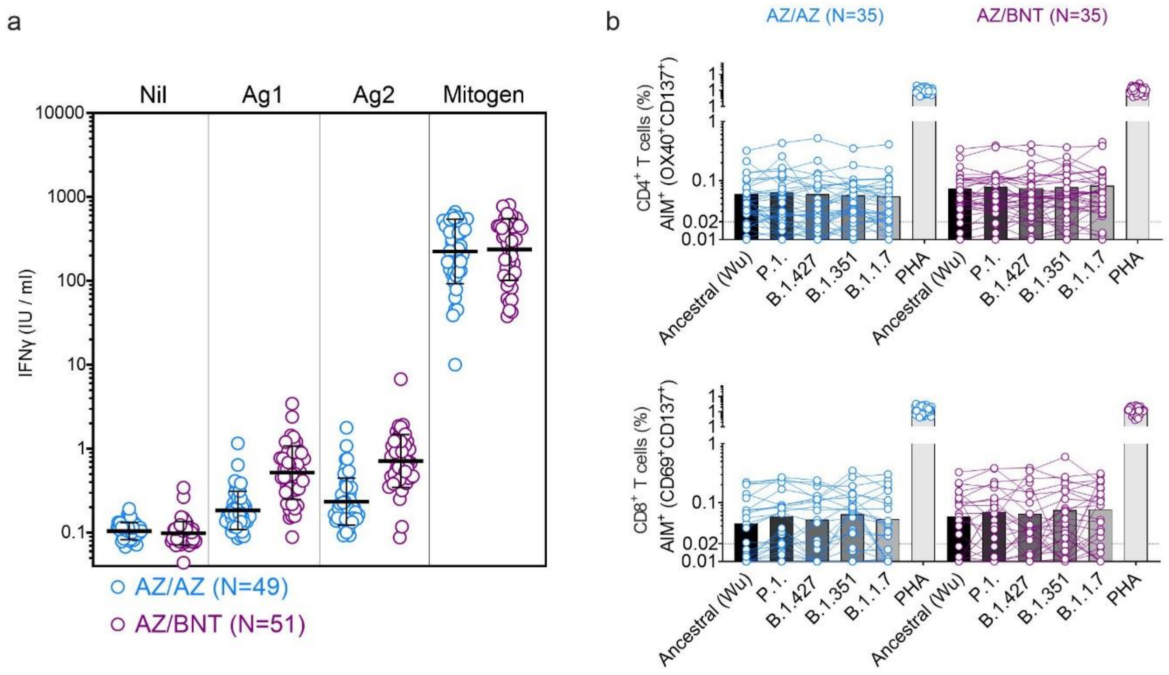

Supplementary Figure S6. T cell responses on day 30 post boost. A. QuantiFERON IFNγ release assay measuring IFNγ (IU/ml) in blood samples from AZ/AZ (blue circles), AZ/BNT (purple circles) vaccine groups at 30 days after boost vaccination. Whole blood samples were either non-stimulated (Nil) or stimulated with specific CD4 (Ag1) and CD4+CD8 (Ag2) SARS-CoV-2 peptide pools from spike antigen (S1 S2 RDB). As positive control Mitogen-stimulated samples were also analyzed. Shown are individual participants, geometric mean and SD. B. Spike-specific AIM$^+$ CD4$^+$ and CD8$^+$ T cells against the ancestral Spike sequence were compared to the Spike sequence derived from the P.1., the B.1.427, the B.1.351 and the B.1.1.7 variants at day 30 post boost. Shown are individual samples and geometric mean (bars).

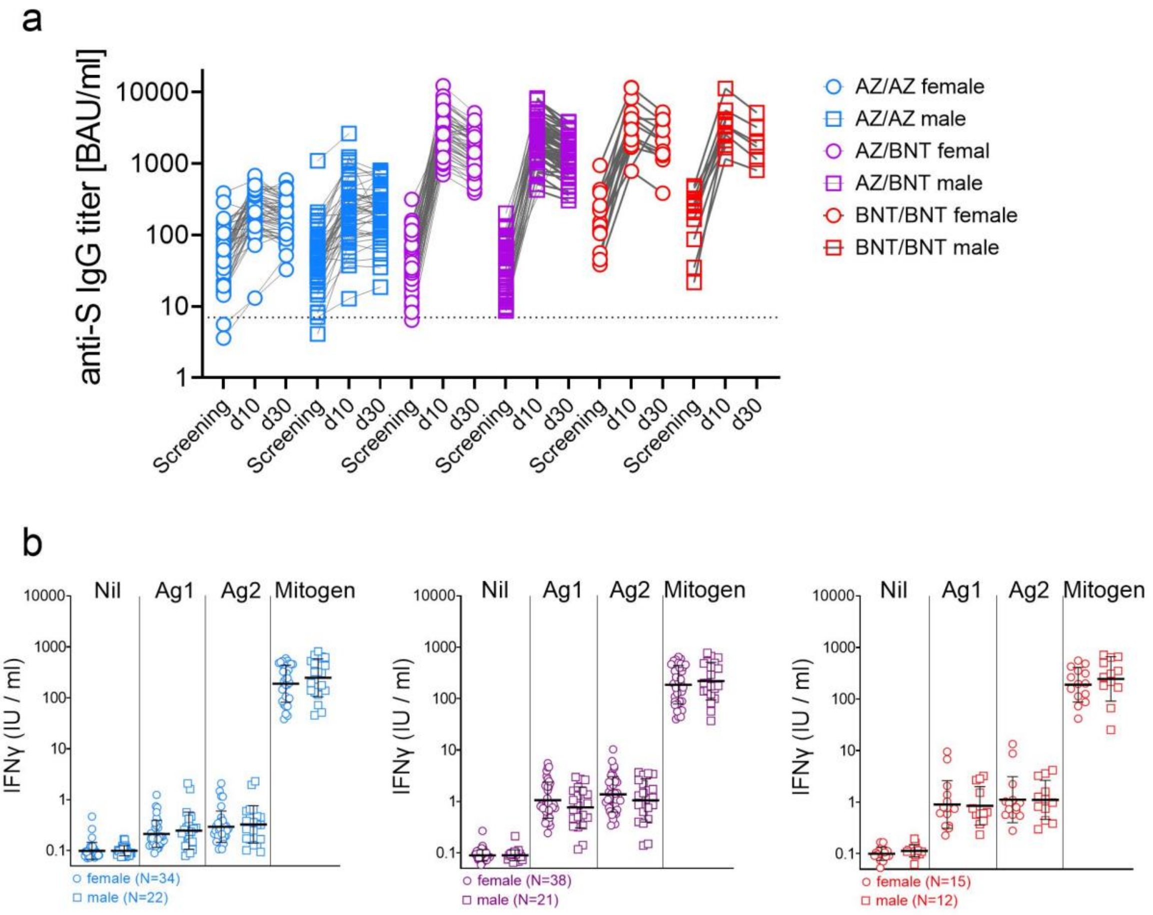

Supplementary Figure S7. Antibody response and IFNγ release are comparable between women and men. A. Titers of anti-S IgG at screening (3-7 days prior to boost), day 10 and day 30 post boost vaccination. Dotted line indicate detection limit (7.1 BAU/ml). B. QuantiFERON IFNγ release assay measuring IFNγ (IU/ml) in blood samples at 10 days after boost vaccination. Individual values and geometric mean ± SD are shown.

Supplementary Tables

Supplementary Table S1. Antibodies for T cell assays

Antibodies used for AIM assays.

Reagent	Clone (Source)	Catalog. No.	Dilution
Live/Dead Blue	(ThermoFisher)	L23105	1:1000
CCR6-BUV496	11A9 (BD Biosciences)	612948	1:200
CXCR5-BV421	J252D4 (Biolegend)	356920	1:200
CXCR3-BV605	G025H7 (Biolegend)	353728	1:200
CCR7-BV711	G043H7 (Biolegend)	353228	1:200
CD3-BUV395	UCHT1 (BD Biosciences)	563546	1:1000
CD137-BUV737	4B4-1 (BD Biosciences)	741861	1:100
CD8-BUV805	SK1 (BD Biosciences)	612889	1:1000
CD16-BV510	3G8 (Biolegend)	302048	1:1000
CD14-BV510	63D3 (Biolegend)	367124	1:1000
CD20-BV510	2H7 (Biolegend)	302340	1:1000
CD45RA-BV570	HI100 (Biolegend)	304132	1:1000
CD38-BV650	HB-7 (Biolegend)	356620	1:200
PD-1-BV785	EH12.2H7 (Biolegend)	329930	1:200
CD69-FITC	FN50 (Biolegend)	310904	1:200
CD4-cFluor b548	SK3 (Cytek Biosciences)	R7-20043	1:500
CD40L-PE-Dazzle594	24-31 (Biolegend)	310840	1:200
OX40-APC	Ber-Act35 (Biolegend)	350008	1:100
HLA-DR-APC-R700	G46-6 (BD Biosciences)	565127	1:500

Antibodies used for AIM assays to detect T cell responses against variants.

Reagent	Clone (Source)	Catalog. No.	Dilution
CD3-FITC	OKT3 (BD Biosciences)	566783	5μl/test, 1:20
CD8 APC-Cy7	RPA-T8 (BD Biosciences)	557760	5μl/test, 1:20
CD4 BV421	SK3 (Biolegend)	300532	5μl/test, 1:20
CD69 PE	FN50 (BD Biosciences)	555531	20μl/test, 1:5
CD134 (OX40) PE-Cy7	Ber-ACT35 (BD Biosciences)	563663	5μl/test, 1:20
CD134 (OX40) PE-Cy7	Ber-ACT35 (Biolegend)	350012	5μl/test, 1:20
CD137 APC	4B4-1 (BD Bioscience)	550890	20μl/test, 1:5
CD14 V500	M5E2 (BD Bioscience)	561391	5μl/test, 1:20
CD19 V500	HIB19 (BD Bioscience)	561121	5μl/test, 1:20
BD Horizon™ Fixable Viability Stain 510	(BD Bioscience)	564406	1:1000

Antibodies used for ICS assays.

Reagent	Clone (Source)	Catalog. No.	Dilution
Live/Dead Blue	(ThermoFisher)	L23105	1:1000
CD3-BUV395	UCHT1 (BD Biosciences)	563546	1:100
CD8-BUV805	SK1 (BD Biosciences)	612889	1:100
CD16-BV510	3G8 (Biolegend)	302048	1:200
CD14-BV510	63D3 (Biolegend)	367124	1:200
CD20-BV510	2H7 (Biolegend)	302340	1:200
CD45RA-BV570	HI100 (Biolegend)	304132	1:50
CD4-cFluor b548	SK3 (Cytek Biosciences)	R7-20043	1:25

CCR7-PE-Cy7	G043H7 (Biolegend)	353226	1:100
IL-4-BUV737	MP4-25D2 (BD Biosciences)	612835	1:200
IL-17-BV785	BL168 (Biolegend)	512338	1:100
IFNg-FITC	4S.B3 (ThermoFisher)	11-7319-82	1:500
IL-2-BB700	MQ1-17H12 (BD Biosciences)	566405	1:200
IL-10 -PE-Dazzle594	JES3-19F1 (Biolegend)	506812	1:100
TNFa-eFluor450	Mab11 (ThermoFisher)	48-7349-42	1:200
Granzyme B-AF647	GB11 (BD Biosciences)	560212	1:50
CD40L-PerCP-ef710	24-31 (ThermoFisher)	46-1548-42	1:50

Supplementary Table S2. Reactogenicity for modified intention to treat population§

		AZ/AZ (n = 108)* Day							AZ/BNT (n = 114)* Day							BNT/BNT (n = 30)* Day						
		1	2	3	4	5	6	7	1	2	3	4	5	6	7	1	2	3	4	5	6	7
Local	Pain injection site	64.8	33.6	14.8	2.8	1.9	1.9	0.9	82.5	37.2	24.6	7.0	4.4	1.8	1.8	80.0	50.0	13.3	3.3	0.0	0.0	0.0
	Erythema (redness) injection site	3.7	0.9	1.9	1.9	0.9	0.9	0.9	7.0	7.1	7.0	2.6	0.9	0.9	0.9	6.7	3.3	3.3	3.3	0.0	0.0	0.0
	Swelling / tissue hardening injection site	12.0	5.6	4.6	0.9	0.0	0.0	0.9	25.4	10.6	17.5	13.2	4.4	3.5	0.9	16.7	6.7	6.7	0.0	0.0	0.0	0.0
	Swelling or sensitivity axillary lymph node	1.9	0.9	0.0	0.0	0.0	0.0	0.0	5.3	7.1	11.4	5.3	6.1	0.9	0.0	13.3	16.7	23.3	16.7	0.0	0.0	0.0
Systemic	Headache	30.6	8.4	4.6	4.6	3.7	5.6	3.7	38.6	15.0	10.5	12.3	8.8	7.9	7.0	53.3	10.0	10.0	6.7	3.3	3.3	0.0
	Tiredness	42.6	5.6	10.2	5.6	4.6	2.8	4.6	50.0	17.7	10.5	7.9	7.0	6.1	5.3	76.7	16.7	20.0	16.7	10.0	10.0	16.7
	Myalgia (muscle pain)	13.0	0.9	0.0	0.9	0.9	0.0	0.0	24.6	7.1	2.6	0.0	0.0	0.0	0.0	26.7	10.0	0.0	0.0	0.0	0.0	0.0
	Arthralgia (joint pain)	10.2	1.9	0.9	0.0	0.0	0.0	0.0	11.4	4.5	4.4	1.8	0.9	0.0	0.0	16.7	6.7	0.0	0.0	0.0	0.0	0.0
	Nausea / vomitus	0.9	0.0	0.0	0.9	0.0	0.0	0.9	2.6	1.8	1.8	0.9	0.9	1.8	0.9	3.3	0.0	0.0	3.3	0.0	0.0	0.0
	Diarrhea	0.9	0.0	0.9	0.0	0.9	0.9	0.0	1.8	1.8	3.5	0.9	1.8	0.9	0.9	0.0	0.0	0.0	0.0	0.0	0.0	0.0
	Increased body temperatura / fever	3.7	0.0	0.9	0.0	0.9	0.9	0.0	14.0	2.7	1.8	0.0	0.0	0.0	0.0	20.0	3.3	0.0	0.0	0.0	0.0	0.0
	Chill	3.7	0.9	0.0	0.0	0.0	0.9	0.0	6.1	0.0	0.0	0.0	0.0	0.0	0.9	3.3	0.0	0.0	3.3	0.0	0.0	0.0
	Rash / nettle rash	0.0	0.0	0.0	0.0	0.0	0.0	0.9	0.0	0.0	0.0	0.9	0.9	0.0	0.0	0.0	0.0	0.0	0.0	0.0	0.0	0.0
	Itching	0.0	0.0	0.9	0.0	0.0	0.9	0.0	1.8	0.9	1.8	0.9	0.9	0.9	0.9	0.0	0.0	3.3	3.3	3.3	3.3	3.3
	Difficulty to breath	0.0	0.0	0.0	0.0	0.0	0.0	0.0	1.8	1.8	0.0	0.0	0.0	0.0	0.0	0.0	0.0	0.0	0.0	0.0	0.0	0.0
	Other	9.3	6.6	2.8	3.7	1.9	1.9	3.7	7.0	8.9	4.4	4.4	5.3	4.4	3.5	20.0	6.7	3.3	3.3	3.3	3.3	3.3

*Shown is the percentage of participants with the respective symptom on days 1-7 after boost vaccination; §modified intention to treat population is defined as all vaccinated individuals who completed at least one follow-up visit after vaccination.

Supplementary Table S3. Effect of heterologous vaccination on binding antibody titers (anti-S IgG)

**anti-S IgG (Figure 2a)** Median (95 % CI)*	**AZ/AZ**	**AZ/ BNT**	**BNT/BNT#**
Screening	49.6 (45.3 - 62.4)	37.8 (32.3 - 47.6)	225.6 (136.3 - 258.2)
Day 10	245.6 (194.0 - 284.9)	2538 (2217 - 3045)	3385 (2399 - 4255)
Day 30	197.8 (161.7 - 227.0)	1478 (1234 - 1826)	2011 (1298 - 3913)

**anti-S IgA (Figure 2b)** Median (95 % CI)§	**AZ/AZ**	**AZ/ BNT**	**BNT/BNT#**
Screening	0.379 (0.316 - 0.451)	0.360 (0.325 - 1.296)	2.387 (1.625 - 3.301)
Day 30	0.655 (0.483 - 0.789)	2.438 (2.014 - 3.021)	6.285 (5.141 - 7.880)

* Anti-S IgG antibody titers are expressed in BAU/ml, values > 7.1 BAU/ml are considered positive; § anti-S IgG antibody titers are expressed as optical density (OD), OD > 1.1 is considered positive; # all comparisons with the non-randomized BNT/BNT arm were exploratory.

Supplementary Table S4. Effect of heterologous vaccination on neutralizing antibody titers

**Ancestral (Figure 2c)** Median (99.9 % CI)*	**AZ/AZ**	**AZ/ BNT**	**BNT/BNT#**
Day 10	73.8 (55.2 - 97.0)	670.4 (506.2 - 896.7)	590.0 (266.1 - 1024)
Day 30	58.1 (44.7 - 74.9)	464.0 (348.2 - 612.5)	433.4 (144.3 - 856.5)

**B.1.1.7 (Figure 2d)** Median (99.9 % CI)*	**AZ/AZ**	**AZ/ BNT**	**BNT/BNT#**
Day 30	163.0 (94.9 - 218.3)	1022 (779.2 - 1024)	1024 (543.9 - 1024)

**B.1.351 (Figure 2d)** Median (99.9 % CI)*	**AZ/AZ**	**AZ/ BNT**	**BNT/BNT#**
Day 30	42.1 (28.4 - 60.8)	295.9 (223.7 - 395.6)	239.6 (44.7 - 423.5)

**B.1.617.2 (Figure 2d)** Median (99.9 % CI)*	**AZ/AZ**	**AZ/ BNT**	**BNT/BNT#**
Day 30	75.9 (58.0 - 132.5)	571.5 (396.6 - 733.1)	404.5 (68.3 - 1024)

* Neutralizing antibody titers are expressed as reciprocal titers; # all comparisons with the non-randomized BNT/BNT arm were exploratory.

Supplementary Table S5. Effect size for neutralizing antibodies (primary endpoint)*

	B.1.1.7		B.1.351		B.1.617.2	
	**AZ/AZ**	**AZ/BNT**	**AZ/AZ**	**AZ/BNT**	**AZ/AZ**	**AZ/BNT**
**Mean**	209.4	827.9	63.58	369.3	140.1	584.2
**Standard derivation**	194.0	253.6	67.96	250.6	176.0	298.4
**Sample size**	102	110	102	110	102	110
**Effect size d_{Cohen}**	2.726		**1.638**		**1.796**	
**95% CI**	2.352 – 3.1		1.327 – 1.95		1.477 – 2.116	

* Calculated with https://www.psychometrica.de/effect_size.html

Supplementary Table S6. Effect of heterologous vaccination on T cell response in Quantiferon IFNɣ release assay

**Tag 10 (Figure 3a)** Geometric mean (95 % CI)*	**AZ/AZ**	**AZ/ BNT**	**BNT/BNT#**
Nil	0.0989 (0.0906 – 0.108)	0.0895 (0.0839 – 0.0955)	0.106 (0.0941 – 0.119)
Ag1	0.226 (0.187 – 0.273)	0.942 (0.754 – 1.18)	0.877 (0.599 – 1.28)
Ag2	0.306 (0.250 – 0.375)	1.25 (0.996 – 1.56)	1.11 (0.765 – 1.62)
Mitogen	210 (168 – 264)	198 (158 – 247)	214 (150 – 305)

**Tag 30 (Figure S6a)** Geometric mean (95 % CI)*	**AZ/AZ**	**AZ/ BNT**	**BNT/BNT#**
Nil	0.105 (0.0980 – 0.112)	0.0989 (0.0903 – 0.108)	n.a
Ag1	0.185 (0.159 – 0.215)	0.521 (0.424 – 0.641)	n.a.
Ag2	0.235 (0.195 – 0.282)	0.717 (0.584 – 0.879)	n.a.
Mitogen	225 (174 – 291)	238 (187 – 302)	n.a.

* IFNɣ release in IU/ml; # all comparisons with the non-randomized BNT/BNT arm were exploratory; n.a. = not analyzed.

Supplementary Table S7. Effect of heterologous vaccination on T cell response in AIM assay

**Total (Figure 3b)** **Day 10** Geometric mean (95 % CI)*	**AZ/AZ**	**AZ/ BNT**	**BNT/BNT#**
$CD4^+$ T cells	0.0989 (0.0684 – 0.143)	0.221 (0.162 – 0.301)	0.165 (0.123 – 0.220)
$CD8^+$ T cells	0.053 (0.037 – 0.076)	0.074 (0.048 – 0.12)	0.059 (0.045 – 0.077)

**B.1.617.2 (Figure 3g)** **Day 10** Geometric mean (95 % CI)*	**AZ/AZ**	**AZ/ BNT**	**BNT/BNT#**
$CD4^+$ T cells - ancestral	0.0695 (0.0414 – 0.117)	0.165 (0.115 – 0.237)	0.165 (0.123 – 0.220)
$CD4^+$ T cells - B.1.617.2	0.0680 (0.0416 – 0.111)	0.138 (0.0950 – 0.200)	0.170 (0.132 – 0.219)
$CD8^+$ T cells- ancestral	0.0350 (0.0272 – 0.0451)	0.0547 (0.0355 – 0.0843)	0.0590 (0.0454 – 0.0769)
$CD8^+$ T cells - B.1.617.2	0.0350 (0.0277 – 0.0442)	0.0488 (0.0336 – 0.0710)	0.0654 (0.0496 – 0.0862)

**Variants (Figure 3h)** **Day 10** Geometric mean (95 % CI)*	**AZ/AZ**	**AZ/ BNT**	**BNT/BNT#**
$CD4^+$ T cells - ancestral	0.0354 (0.0272 – 0.0461)	0.0992 (0.0753 – 0.131)	0.102 (0.0758 – 0.138)
$CD4^+$ T cells - P.1	0.0331 (0.0251 – 0.0437)	0.0928 (0.0698 – 0.123)	0.0872 (0.0613 – 0.124)
$CD4^+$ T cells - B.1.427	0.0250 (0.0192 – 0.0326)	0.0755 (0.0559 – 0.102)	0.0777 (0.0544 – 0.111)
$CD4^+$ T cells - B.1.351	0.0317 (0.0239 – 0.0419)	0.0992 (0.0766 – 0.129)	0.0855 (0.0609 – 0.120)
$CD4^+$ T cells - B.1.1.7	0.0335 (0.0247 – 0.0456)	0.0936 (0.0713 – 0.123)	0.0940 (0.0660 – 0.134)
$CD4^+$ T cells - PHA	8.73 (7.60 – 10.0)	8.89 (7.56 – 10.5)	10.2 (8.54 – 12.1)
$CD8^+$ T cells- ancestral	0.0239 (0.0168 – 0.0341)	0.0579 (0.0377 – 0.0888)	0.0328 (0.0208 – 0.0517)
$CD8^+$ T cells - P.1	0.0226 (0.0160 – 0.0319)	0.0504 (0.0325 – 0.0782)	0.0355 (0.0230 – 0.0550)
$CD8^+$ T cells - B.1.427	0.0220 (0.0159 – 0.0306)	0.0457 (0.0297 – 0.0704)	0.0324 (0.0206 – 0.0510)
$CD8^+$ T cells - B.1.351	0.0234 (0.0165 – 0.0332)	0.0580 (0.0364 – 0.0924)	0.0347 (0.0218 – 0.0553)
$CD8^+$ T cells - B.1.1.7	0.0224 (0.0159 – 0.0317)	0.0490 (0.0306 – 0.0785)	0.0448 (0.0272 – 0.0737)
$CD8^+$ T cells - PHA	11.3 (9.49 – 13.6)	10.4 (8.73 – 12.3)	13.3 (11.5 – 15.5)

**Variants (Figure S6b)** **Day 30** Geometric mean (95 % CI)*	**AZ/AZ**	**AZ/ BNT**	**BNT/BNT#**
$CD4^+$ T cells - ancestral	0.0396 (0.0294 – 0.0533)	0.0504 (0.0372 – 0.0681)	n.a.
$CD4^+$ T cells - P.1	0.0362 (0.0255 – 0.0513)	0.0546 (0.0406 – 0.0733)	n.a.
$CD4^+$ T cells - B.1.427	0.0331 (0.0235 – 0.0465)	0.0515 (0.0386 – 0.0686)	n.a.
$CD4^+$ T cells - B.1.351	0.0382 (0.0283 – 0.0516)	0.0534 (0.0393 – 0.0723)	n.a.
$CD4^+$ T cells - B.1.1.7	0.0339 (0.0248 – 0.0464)	0.0553 (0.0408 – 0.0749)	n.a.
$CD4^+$ T cells - PHA	10.4 (9.14 – 11.9)	10.1 (8.57 – 11.9)	n.a.
$CD8^+$ T cells- ancestral	0.0226 (0.0155 – 0.0329)	0.0273 (0.0180 – 0.0414)	n.a.
$CD8^+$ T cells - P.1	0.0287 (0.0192 – 0.0429)	0.0308 (0.0199 – 0.0474)	n.a.
$CD8^+$ T cells - B.1.427	0.0252 (0.0169 – 0.0375)	0.0301 (0.0202 – 0.0449)	n.a.
$CD8^+$ T cells - B.1.351	0.0319 (0.0212 – 0.0482)	0.0325 (0.0212 – 0.0499)	n.a.
$CD8^+$ T cells - B.1.1.7	0.0245 (0.0165 – 0.0365)	0.0366 (0.0236 – 0.0568)	n.a.
$CD8^+$ T cells - PHA	12.2 (9.92 – 14.9)	11.4 (9.32 – 13.9)	n.a.

* spike-specific T cell responses in the AIM assay by measuring the surface expression of activation-induced cell markers CD134 (OX40) and CD137 on $CD4^+$, and CD69 and CD137 on $CD8^+$ T cells; # all comparisons with the non-randomized BNT/BNT arm were exploratory; n.a. = not analyzed.

Supplementary Table S8. Effect of heterologous vaccination on T cell response in intracellular cytokine staining assay

**CD4$^+$ (Figure 3c + e + S2c)** **Day 10** Geometric mean (95 % CI)*	**AZ/AZ**	**AZ/ BNT**	**BNT/BNT#**
IFNγ$^+$	0.0094 (0.0068 – 0.013)	0.031 (0.023 – 0.042)	0.016 (0.011 – 0.023)
TNFα$^+$	0.0085 (0.0065 – 0.011)	0.019 (0.014 – 0.026)	0.016 (0.011 – 0.023)
IL-2$^+$	0.0086 (0.0066 – 0.011)	0.014 (0.011 – 0.019)	0.013 (0.010 – 0.018)
Granzyme B$^+$	0.0069 (0.0053 – 0.0090)	0.018 (0.011 – 0.029)	0.010 (0.0070 – 0.015)
IL-10$^+$	0.0050 (0.0050 – 0.0050)	0.0065 (0.0054 – 0.0078)	n.a.
IL-4$^+$	0.0050 (0.0050 – 0.0050)	0.0050 (0.0050 – 0.0050)	n.a.
IL-17$^+$	0.0050 (0.0050 – 0.0050)	0.0050 (0.0050 – 0.0050)	n.a.
Any cytokine	0.0736 (0.0431 – 0.126)	0.161 (0.0947 – 0.272)	0.167 (0.118 – 0.236)

**CD8$^+$ (Figure 3d + f)** **Day 10** Geometric mean (95 % CI)*	**AZ/AZ**	**AZ/ BNT**	**BNT/BNT#**
IFNγ$^+$	0.0077 (0.0053 – 0.011)	0.012 (0.0075 – 0.018)	0.0071 (0.0052 – 0.0096)
TNFα$^+$	0.0068 (0.0053 – 0.0086)	0.0060 (0.0050 – 0.0071)	0.0066 (0.0052 – 0.0084)
IL-2$^+$	0.0051 (0.0049 – 0.0054)	0.0052 (0.0048 – 0.0057)	0.0050 (0.0050 – 0.0050)
Granzyme B$^+$	0.0050 (0.0050 – 0.0050)	0.0050 (0.0050 – 0.0050)	0.0050 (0.0050 – 0.0050)
Any cytokine	0.010 (0.0069 – 0.016)	0.014 (0.0092 – 0.022)	0.010 (0.0071 – 0.015)

* % spike-specific CD4$^+$ T cells expressing intracellular CD40L (iCD40L) and producing IFNɣ, TNFα, IL-2, granzyme B, IL-10, IL-4, IL-17, or any cytokine and spike-specific CD8$^+$ T cells producing IFNɣ, TNFα, IL-2 , granzyme B, IL-10, IL-4, IL-17, or any cytokine by intracellular cytokine staining; # all comparisons with the non-randomized BNT/BNT arm were exploratory; n.a. = not analyzed.

Supplementary Table S9 Efficacy for primary and secondary endpoints as specified in study protocol.

Endpoint	Primary/ secondary	Comparison	P value*	Median (99.9 % CI) for prim. endpoint; geom. mean (95 % CI) for sec. endpoint	Comment
1a Level of neutralizing antibodies against wild-type and immune escape variants in the 3 study groups at 10, 30, 90 and 180 days post boost	Primary	AZ/AZ vs. AZ/BNT - Level of nab against wild-type on day 10	<0.0001	73.8 (55.2 - 96.99) vs. 670.4 (506.2 - 896.7)	No day 90 and day 180 comparison as interim analysis presented here
		AZ/AZ vs. AZ/BNT - Level of nab against wild-type on day 30	<0.0001	58.1 (44.7 - 74.9) vs. 464.0 (348.2 - 612.5)	
		AZ/AZ vs. AZ/BNT - Level of nab against B.1.1.7 on day 30	<0.0001	163.0 (94.9 - 218.3) vs. 1022 (779.2 - 1024)	
		AZ/AZ vs. AZ/BNT - Level of nab against B.1.351 on day 30	<0.0001	42.1 (28.4 - 60.8) vs. 295.9 (223.7 - 395.6)	
		AZ/AZ vs. AZ/BNT - Level of nab against B.1.617.2 on day 30	<0.0001	75.9 (58.0 - 132.5) vs. 571.5 (396.6 - 733.1)	
1b Level of neutralizing antibodies against wild-type and immune escape variants at 10, 30, 90 and 180 days post 3rd immunization	Primary	n.a.	-	n.a.	N.a. as interim analysis at day 30 after 2nd immunization
1 Level of T cell responses against SARS-CoV-2 spike protein peptides pools per subgroup at 30 days.	Secondary	AZ/AZ vs. AZ/BNT - QuantiFERON IFNγ release assay on day 30 Nil	-	0.105 (0.0980 - 0.112) vs. 0.0989 (0.0903 - 0.108)	
		AZ/AZ vs. AZ/BNT - QuantiFERON IFNγ release assay on day 30 Ag1	-	0.185 (0.159 - 0.215) vs. 0.521 (0.424 - 0.641)	
		AZ/AZ vs. AZ/BNT - QuantiFERON IFNγ release assay on day 30 Ag2	-	0.235 (0.195 - 0.282) vs. 0.717 (0.584 - 0.879)	
		AZ/AZ vs. AZ/BNT - QuantiFERON IFNγ release assay on day 30 Mitogen	-	225 (174 - 291) vs. 238 (187 - 302)	
2 Occurrence of breakthrough infection with wild type, B.1.351 and other immune escape variants of SARS-CoV-2	Secondary	n.a.	-	n.a.	N.a. as interim analysis at day 30 after 2nd immunization
3 Occurrence of adverse events, serious adverse events and adverse events of special interest	Secondary	Safety analysis has been done in a descriptive way			No study related serious adverse events
4 The clinical course (hospitalization, ICU admission) and outcome of COVID-19 diseases (recovery, long-Covid mortality)	Secondary	n.a.	-	n.a.	N.a. as interim analysis after 2nd immunization

5 Occurrence of adverse events, serious adverse events and adverse events of special interest after 3rd immunization	Secondary	n.a.	-	n.a.	N.a. as interim analysis after 2nd immunization
6 Level of T cell responses against SARS-CoV-2 spike protein peptides pools in non-responders after 3rd immunization	Secondary	n.a.	-	n.a.	N.a. as interim analysis after 2nd immunization
7 Occurrence of breakthrough infection with wild type, B.1.351 and other immune escape variants of SARS-CoV-2 after 3rd immunization	Secondary	n.a.	-	n.a.	N.a. as interim analysis after 2nd immunization

*P value <0.001 (significance level according to stopping rule of the interim analysis as applied for the comparison of the primary endpoint neutralizing antibodies between AZ/AZ and AZ/BNT arms). All other comparisons were exploratory using confidence intervals (see Supplementary Table S4 for 99.9 % CI of neutralizing antibodies and Supplementary Table S6-S8 for 95 % CI for T cell responses for all groups); n.a. = not applicable as this endpoint has not been analyzed as part of the interim analysis on day 30.

Supplementary Table S10. GISAID sequences used

Virus name	Accession No.	Collected	Originating laboratory	Submitting laboratory*	Authors
hCoV-19/England/MILK-9E05B3/2020	EPI_ISL_601443	2020-09-20	Lighthouse Lab in Milton Keynes	Wellcome Sanger Institute for the COVID-19 Genomics UK (COG-UK) consortium	The Lighthouse Lab in Milton Keynes and Alex Alderton, Roberto Amato, Sonia Goncalves, Ewan Harrison, David K. Jackson, Ian Johnston, Dominic Kwiatkowski, Cordelia Langford, John Sillitoe on behalf of the Wellcome Sanger Institute COVID-19 Surveillance Team (http://www.sanger.ac.uk/covid-team)
hCoV-19/Brazil/AM-L70-CD1722/2020	EPI_ISL_804823	2020-12-17	DB Diagnosticos do Brasil	Laboratório de Parasitologia Médica - Instituto de Medicina Tropical - Universidade de São Paulo	Nuno Faria, Ingra Morales Claro, Darlan Candido, Lucas A. Moyses Franco, Pamela dos Santos Andrade, Thais de Moura Coletti, Camila A. Maia da Silva, Flavia Cristina Sales, Erika Regina Manuli, Renato A. Santana, Nelson Gaburo, Cecília da Cunha Camilo, Nelson Abrahim Fraiji, Myuki Alfaia Esashika Crispim, Maria do Perpétuo Socorro Sampaio Carvalho, Andrew Rambaut, Nick Loman, Oliver G. Pybus, Ester C. Sabino; DB; HEMOAM; CDL; CADDE Genomic Network.
hCoV-19/USA/CA-UCSF-UC684/2020	EPI_ISL_847619	2020-12-20	Chiu Laboratory, University of California, San Francisco	Chiu Laboratory, University of California, San Francisco	Charles Chiu, Xianding (Wayne) Deng, Candace Wang, Brian Bushnell, Scot Federman, Jill Hacker, Debra Wadford
hCoV-19/USA/CA-UCSF-UC689/2020	EPI_ISL_847621	2020-12-20	Chiu Laboratory, University of California, San Francisco	Chiu Laboratory, University of California, San Francisco	Charles Chiu, Xianding (Wayne) Deng, Candace Wang, Brian Bushnell, Scot Federman, Jill Hacker, Debra Wadford
hCoV-19/USA/CA-UCSF-UC698/2020	EPI_ISL_847643	2020-12-15	Chiu Laboratory, University of California, San Francisco	Chiu Laboratory, University of California, San Francisco	Charles Chiu, Xianding (Wayne) Deng, Candace Wang, Brian Bushnell, Scot Federman, Jill Hacker, Debra Wadford
hCoV-19/South Africa/KRISP-K004599/2020	EPI_ISL_660629	2020-11-06	NHLS-IALCH	KRISP, KZN Research Innovation and Sequencing Platform	Giandhari J, Pillay S, Lessells R, Mdlalose K, York D, Khan S, Tegally H, Wilkinson E, de Oliveira T

hCoV-19/South Africa/KRISP-MDSH920854/2020	EPI_ISL_736930	2020-12-06	MDS	KRISP, KZN Research Innovation and Sequencing Platform	Giandhari J, Pillay S, Lessells R, ChimukangaraB, Mdlalose K, York D, Khan S, Tegally H, Wilkinson E, de Oliveira T
hCoV-19/South Africa/KRISP-BH02956385/2020	EPI_ISL_736932	2020-11-25	NHLS-IALCH	KRISP, KZN Research Innovation and Sequencing Platform	Giandhari J, Pillay S, Lessells R, ChimukangaraB, Mdlalose K, York D, Khan S, Tegally H, Wilkinson E, de Oliveira T
hCoV-19/South Africa/KRISP-CD02410157/2020	EPI_ISL_736944	2020-11-24	NHLS-IALCH	KRISP, KZN Research Innovation and Sequencing Platform	Giandhari J, Pillay S, Lessells R, ChimukangaraB, Mdlalose K, York D, Khan S, Tegally H, Wilkinson E, de Oliveira T
hCoV-19/South Africa/KRISP-MDSH920866/2020	EPI_ISL_736966	2020-12-06	MDS	KRISP, KZN Research Innovation and Sequencing Platform	Giandhari J, Pillay S, Lessells R, ChimukangaraB, Mdlalose K, York D, Khan S, Tegally H, Wilkinson E, de Oliveira T
hCoV-19/South Africa/KRISP-EC-MDSH924814/2020	EPI_ISL_736971	2020-12-08	NHLS-IALCH	KRISP, KZN Research Innovation and Sequencing Platform	Giandhari J, Pillay S, Lessells R, ChimukangaraB, Mdlalose K, York D, Khan S, Tegally H, Wilkinson E, de Oliveira T
hCoV-19/South Africa/KRISP-EC-MDSH925012/2020	EPI_ISL_736973	2020-12-09	NHLS-IALCH	KRISP, KZN Research Innovation and Sequencing Platform	Giandhari J, Pillay S, Lessells R, ChimukangaraB, Mdlalose K, York D, Khan S, Tegally H, Wilkinson E, de Oliveira T
hCoV-19/South Africa/KRISP-K006830/2020	EPI_ISL_825104	2020-11-19	NHLS-IALCH	KRISP, KZN Research Innovation and Sequencing Platform	Giandhari J, Pillay S, Lessells R, Mdlalose K, York D, Khan S, Tegally H, Wilkinson E, de Oliveira T
hCoV-19/South Africa/KRISP-K006863/2020	EPI_ISL_825120	2020-11-24	NHLS-IALCH	KRISP, KZN Research Innovation and Sequencing Platform	Giandhari J, Pillay S, Lessells R, Mdlalose K, York D, Khan S, Tegally H, Wilkinson E, de Oliveira T
hCoV-19/South Africa/KRISP-K007622/2020	EPI_ISL_825131	2020-12-12	NHLS-IALCH	KRISP, KZN Research Innovation and Sequencing Platform	Giandhari J, Pillay S, Lessells R, Mdlalose K, York D, Khan S, Tegally H, Wilkinson E, de Oliveira T

*All submitters of data may be contacted directly via www.gisaid.org

Supplementary References

1. Riepler L, Rössler A, Falch A, et al. Comparison of Four SARS-CoV-2 Neutralization Assays. Vaccines (Basel) 2020;9.
2. Ferrara F, Temperton N. Pseudotype Neutralization Assays: From Laboratory Bench to Data Analysis. Methods Protoc 2018;1.
3. Tarke A, Sidney J, Methot N, et al. Impact of SARS-CoV-2 variants on the total CD4(+) and CD8(+) T cell reactivity in infected or vaccinated individuals. Cell reports Medicine 2021;2:100355.
4. Carrasco Pro S, Sidney J, Paul S, et al. Automatic Generation of Validated Specific Epitope Sets. Journal of Immunology Research 2015;2015:763461.
5. Dan JM, Mateus J, Kato Y, et al. Immunological memory to SARS-CoV-2 assessed for up to 8 months after infection. Science 2021;371.
6. Grifoni A, Weiskopf D, Ramirez SI, et al. Targets of T Cell Responses to SARS-CoV-2 Coronavirus in Humans with COVID-19 Disease and Unexposed Individuals. Cell 2020;181:1489-501 e15.
7. Rydyznski Moderbacher C, Ramirez SI, Dan JM, et al. Antigen-Specific Adaptive Immunity to SARS-CoV-2 in Acute COVID-19 and Associations with Age and Disease Severity. Cell 2020;183:996-1012.e19.
8. Kotturi MF, Peters B, Buendia-Laysa F, Jr., et al. The CD8+ T-cell response to lymphocytic choriomeningitis virus involves the L antigen: uncovering new tricks for an old virus. J Virol 2007;81:4928-40.
9. Mateus J, Grifoni A, Tarke A, et al. Selective and cross-reactive SARS-CoV-2 T cell epitopes in unexposed humans. Science 2020;370:89-94.
10. Mateus J, Dan JM, Zhang Z, et al. Low-dose mRNA-1273 COVID-19 vaccine generates durable memory enhanced by cross-reactive T cells. Science 2021;374:eabj9853.
