# Supplementary material for: Heterologous ChAdOx1/BNT162b2 vaccination induces stronger immune response than homologous ChAdOx1 vaccination: The pragmatic, multi-center, three-arm, partially randomized HEVACC trial
Source: eBioMedicine. 2022 May 23;80:104073. doi: 10.1016/j.ebiom.2022.104073 (PMC9126042; doi:10.1016/j.ebiom.2022.104073)
Supplement: Supplementary file 2 — Study Protocol. [file mmc2.pdf]

Clinical Investigation Plan

Heterologous vaccination with an Vaxzevria (ChAdOx1-S) prime and a Comirnaty (BNT162b2) boost compared with homolog vaccination with Vaxzervria (prime/boost) or Comirnaty (prime/boost)

HEVACC

EudraCT No. 2021-002171-19
Version: 1.3
Date: 18.08.2021

Confidentiality

The information provided in this clinical investigation plan are strictly confidential and will only be available for potential investigators, involved investigators and their study team as well as for the medical director of the conducting hospital, health authorities and ethics committees to review, verify or implement the clinical trial. Any publication or disclosure to a third party without prior written consent of the sponsor is expressly prohibited. By signing the clinical investigation plan, the provisions of this clinical investigation plan are for all parties binding.

General Information

**Sponsor of the Clinical Trial**	Medical University Innsbruck<br>Institute of Virology<br>Univ.-Prof. Dr. Dorothee von Laer<br>Peter-Mayr-Straße 4b<br>6020 Innsbruck, Austria
**Coordinating Investigator of the Clinical Trial**	Medical University Innsbruck<br>Institute of Virology<br>Prof. Dr. Dorothee von Laer<br>Peter-Mayr-Straße 4b<br>6020 Innsbruck, Austria
**Author of the Clinical Investigation Plan**	Medical University Innsbruck<br>Institute of Virology<br>Prof. Dr. Dorothee von Laer<br>Peter-Mayr-Straße 4b<br>6020 Innsbruck, Austria
**Statistician**	Medical University Innsbruck<br>Department of Medical Statistics,<br>Prof. Dr. H. Ulmer<br>Informatics and Health Economics<br>Schöpfstraße 41/1, 6020 Innsbruck, Austria

Signatures

**Study Title:**	Heterologous vaccination with a Vaxzevria (ChAdOx1-S) prime and a Comirnaty (BNT162b2) boost compared with homolog vaccination with Vaxzervria (prime/boost) or Comirnaty (prime/boost)
**Short Title:**	HEVACC
**EudraCT-No.:**	2021-002171-19
**Investigational Medicinal Product:**	**Vaxzevria ChAdOx1-S** [COVID-19 Vaccine AstraZeneca suspension for injection COVID-19 Vaccine (ChAdOx1-S [recombinant])]<br>**Comirnaty BNT162b2** [Comirnaty concentrate for dispersion for injection COVID-19 mRNA Vaccine (nucleoside modified)]

Declaration of the Sponsor

The present clinical investigation plan (CIP) was subject to critical review. Its content is consistent with the current risk/benefit evaluation of the investigational medicinal product (IMP) as well as with moral, ethical and scientific principles of good clinical practice (GCP), the latest version of the Declaration of Helsinki, the local laws and regulations as well as applicable regulatory requirements.
With the signature below the person confirms to have read this Clinical Investigation Plan and to agree that it contains all information required for study performance. Furthermore, the person agrees to conduct the study as set in this CIP and to adhere to the sponsor's standard operation procedure (SOPs), if provided and as far as agreed. It has been understood that all documentation previously not published will be kept confidential. Furthermore, the person agrees to take all necessary measures to ensure safety and confidentiality of the patient´s identities.

Univ.-Prof. Dr. Dorothee von Laer<br>Medical University of Innsbruck<br>Head of Institute of Virology	____________________________<br>Place, Date Signature

Signatures

**Study Title:**	Heterologous vaccination with a Vaxzevria (ChAdOx1-S) prime and a Comirnaty (BNT162b2) boost compared with homolog vaccination with Vaxzervria (prime/boost) or Comirnaty (prime/boost)
**Short Title:**	HEVACC
**EudraCT-No.:**	2021-002171-19
**Investigational Medicinal Product:**	**Vaxzevria ChAdOx1-S** [COVID-19 Vaccine AstraZeneca suspension for injection COVID-19 Vaccine (ChAdOx1-S [recombinant])]<br>**Comirnaty BNT162b2** [Comirnaty concentrate for dispersion for injection COVID-19 mRNA Vaccine (nucleoside modified)]

Declaration of the Author, the Coordinating Principal Investigator and the Statistician

The present CIP was subject to critical review. Its content is consistent with the current risk/benefit evaluation of the IMP as well as with moral, ethical and scientific principles of GCP, the latest version of the Declaration of Helsinki, the local laws and regulations as well as applicable regulatory requirements.
With their signature below the persons confirm to have read this Clinical Investigation Plan and to agree that it contains all information required for study performance. Furthermore, the persons agree to conduct the study as set in this CIP and to adhere to the sponsor's SOPs (as far as agreed and provided). It has been understood that all documentation previously not published will be kept confidential. Furthermore, the persons agree to keep patient´s identities in strictest confidence and to take all required measures to ensure this confidentiality.

Univ.-Prof. Dr. Dorothee von Laer<br>Medical University of Innsbruck<br>Author	______________________________<br>Place, Date, Signature
Univ.-Prof. Dr. Dorothee von Laer<br>Medical University of Innsbruck<br>Coordinating Investigator and Principal Investigator - Innsbruck	______________________________<br>Place, Date, Signature
ao. Univ.Prof. Mag. Dr. Hanno Ulmer<br>Medical University of Innsbruck<br>Statistician	______________________________<br>Place, Date, Signature

List of Study Sites

List name and address of each study site and study site investigator/s including full contact detail such as telephone/fax number and email address as shown below:

**A01**	**Medical University Innsbruck, Institute of Virology,** Peter-May-Straße 4b, 6020 Innsbruck Austria<br><br>**Principal Investigator**<br>Univ.-Prof. Dr. Dorothee von Laer
**A02**	**Bezirkskrankenhaus Kufstein,** Endach 27, 6330 Kufstein, Austria<br>direktion@bkh-kufstein.at<br><br>**Principal Investigator**<br>Primar Dr. Tobias Trips
**A03**	**Bezirkskrankenhaus Schwaz,** Swarovskistraße 1-3, 6130 Schwaz , Austria<br><br>**Principal Investigator**<br>Dr. Daniel Rainer
**A04**	**Medical University of Vienna, Center for Pathophysiology, Infectiology and Immunology, Institute of Specific Prophylaxis and Tropical Medicine**, Kinderspitalgasse 15, 1090 Wien<br><br>**Principal Investigator**<br>Univ.-Prof. Dr. Ursula Wiedermann Schmidt

Site-specific Signatures

**Study Title:**	Heterologous vaccination with an Vaxzevria (ChAdOx1-S) prime and a Comirnaty (BNT162b2) boost compared with homolog vaccination with Vaxzervria (prime/boost) or Comirnaty (prime/boost)
**Short Title:**	HEVACC
**EudraCT-No.:**	2021-002171-19
**Investigational Medicinal Product:**	**Vaxzevria ChAdOx1-S** [COVID-19 Vaccine AstraZeneca suspension for injection COVID-19 Vaccine (ChAdOx1-S [recombinant])]<br>**Comirnaty BNT162b2** [Comirnaty concentrate for dispersion for injection COVID-19 mRNA Vaccine (nucleoside modified)]

Declaration of the Principal (site specific) Investigator

The present CIP was subject to critical review. Its content is consistent with the current risk/benefit evaluation of the IMP as well as with moral, ethical and scientific principles of GCP, the latest version of the Declaration of Helsinki, the local laws and regulations as well as applicable regulatory requirements.
With their signature below the persons confirm to have read this CIP and to agree that it contains all information required for study performance. Furthermore, the person agrees to conduct the study as set in this CIP and to adhere to the sponsor's SOPs (as far as agreed and provided). It has been understood that all documentation previously not published will be kept confidential. Furthermore, the persons agree to keep patient´s identities in strictest confidence and to take all required measures to ensure this confidentiality.

It is agreed that the clinical trial will be conducted according to Austrian pharmaceutical act (AMG) as well as to the ICH-GCP Guideline (ICH-E6, CPMP/ICH/135/95) and the latest version of the Declaration of Helsinki.

To ensure data quality, clinical trial´s integrity and compliance with the CIP as well as with the various legal and regulatory requirements the sponsor will visit the participating sites.
With their signature below the persons agree to support visits of authorized persons (e.g. representatives of the sponsor) and to provide them directly to enter the source and other relevant documents in regard to the clinical trial (e.g. CRF, patient files).

**Univ.-Prof. Dr. Dorothee von Laer**<br>Medical University Innsbruck /<br><br>Principal Investigator	______________________________<br>Place, Date, Signature

Additional involved Persons and Institutions

List name and address of each additional involved person or institution including full contact detail such as telephone/fax number and email address as shown below:

**Data Safety Monitoring Board**	Prof. Dr. Florian Krammer, Krammer Laboratory, Department of Microbiology, Icahn School of Medicine at Mount Sinai<br>One Gustave L. Levy Place, Box 1124, New York, NY 10029<br><br>Dr. Marton Széll<br>Am Heumarkt 3/1/11<br>1030 Wien<br><br>Prof. Dr. Michael Kundl<br>Abteilung für Umwelthygiene und Umweltmedizin<br>Medizinische Universität Wien<br>Zentrum für Public Health<br>Kinderspitalgasse 15<br>1090 Wien
**Laboratory**	Medical University Innsbruck<br>Contact person: Institute of Virology<br>Peter Mayr Straße 4b, 6020 Innsbruck<br>Dr. Janine Kimpel
**Data Management**	Medical University Innsbruck<br>**ASKIMED**<br>Tel: +43 512 9003-70562<br>E-Mail: askimed@i-med.ac.at
**Monitoring**	Medical University Innsbruck<br>**OE CCCT**<br>Competence Center for Clinical Trials<br>Anichstraße 35, 6020 Innsbruck, Austria<br>Tel. +43 512 9003 70085, Fax +43 512 9003 73086<br>E-Mail: kks-innsbruck@i-med.ac.at

Table of Content

1. Introduction 23

1.1 Study Background 23

1.2 Preclinical and Clinical Data 23

1.3 Need of the Study 24

1.4 Risk-Benefit Analysis 25

2. Study Objectives and Endpoints 25

2.1 Primary Study Objectives and Endpoints 25

2.2 Secondary Study Objectives and Endpoints 26

3. Study Design 27

3.1 Study Description 27

3.2 Timetable 27

4. Study Population 27

4.1 Number of Patients 28

4.2 Inclusion Criteria 28

4.3 Exclusion Criteria 30

4.4 Withdrawal of study subjects after study start/Discontinuation of Study Treatment 31

5. Study Procedures 32

5.1 General Study Procedures and Assessments Schedule 32

5.2 Screening Visit (Day - 7 to -3) 33

5.3 Visit I (Day 0, Day of Boost shot) 33

5.4 Visit III (Day 2 after boost shot) 34

5.5 Visit II, IV - VIII (Day 1, 3 – 7 after boost shot) 35

5.6 Visit IX (Day 10 ± 1 day) 35

5.7 Visit VIII, X – XXXIII (every 7 days ± 2 day after boost shot) 36

5.8 Additional visits on Day 30 (AV 1), Day 90 (AV 2) and Day 180 (AV 3) after boost shot (± 3 day) 36

5.9 Unscheduled Visits 36

5.10 Study Assessments 36

5.11 Discontinuation of the Study/Premature Termination of the Trial 38

5.12 Closure of the Study 42

6. Study Medication (IMP) 42
6.1 Vaxzevria Suspension for injection and Comirnaty concentrate for dispersion for injection. 42
6.2 Dosage and Administration 42
6.3 Dose Modifications 43
6.4 Concomitant Medication 43
6.5 Labelling 45
6.6 Handling of IMP at the Site and Drug Accountability 45
6.7 Subject Compliance 45
6.8 Blinding and Unblinding 46
7. Adverse Events 46
7.1 Summary of known and possible Adverse Events of the IMP 46
7.2 Adverse Events 50
7.3 Serious Adverse Events 51
7.4 Adverse Drug Reaction (ADR) & Unexpected Adverse Drug Reaction 52
7.5 Suspected Unexpected Serious Adverse Reaction (SUSAR) 52
7.6 Pregnancy 53
7.7 Grading of Severity of Adverse Events 53
7.8 Causality 54
7.9 Reporting Procedures 54
8. Documentation 57
8.1 Data Recording (CRF/eCRF) 57
8.2 Trial Folders 57
8.3 Data Storage 58
9. Data Management 58
10. Protocol Deviations 59
11. Statistics 59
11.1 Sample Size 59
11.2 Randomization 60
11.3 Statistical Design, Methods and Analysis process 60
12. Quality Management 62
12.1 Qualifications 62

12.2 Monitoring 63

12.3 Audits and Inspections 63

13. Reporting 64

13.1 Final Study Report 64

13.2 Publication 64

14. Amendments 64

15. Ethical and Regulatory Aspects 64

15.1 Responsibilities of Sponsor and Investigator 64

15.2 Approval of Ethics Committee and Notification to the Authority 65

15.3 Patient Information and Consent Form 65

15.4 Insurance 65

15.5 Data Protection and Confidentiality 66

15.6 Financing 66

15.7 Regulatory Aspects 66

16. DSMB 66

17. Literature 66

Abbreviations and Definitions

ADR	Adverse Drug Reaction
AE	Adverse Event
AMG	Austrian Pharmaceutical Act
CDM	Clinical Data Management
CDMS	Clinical Data Management System
CI	Coordinating Investigator
CRF	Case Report Form
CRO	Contract Research Organization
CTCAE	Common Terminology Criteria for Adverse Events
DCF	Data Clarification Form
DIBD	Development International Birth Date
DMC	Data Monitoring Committee
DMP	Data Management Plan
DSMB	Data and Safety Monitoring Board
DVP	Data Validation Plan
eCRF	electronic Case Report Form
EEA	European Economic Area (
EC	Ethics Committee
ECG	Electrocardiogram
EU	European Union
EudraCT	European Clinical Trials Database
FPFV	First Patient First Visit
GCP	Good Clinical Practice
GMP	Good Manufacturing Practice
IB	Investigator‘s Brochure
ICF	Informed Consent Form
ICH	International Conference on Harmonization
IDMC	Independent Medicinal Product Dossier
IMP	Investigational Medicinal Product
IMPD	Independent data monitoring committee
IRB	Institutional Review Board
ISF	Investigator Site File
LPLV	Last Patient Last Visit
LTFU	Lost To Follow-Up
MedDRA	Medical Dictionary for Regulatory Activities
PCI	Principal Coordinating Investigator
pCRF	paperbased Case Report Form
PFS	Progression-Free Survival
PI	Principal Investigator
SAE	Serious Adverse Event
SAP	Statistical Analysis Plan
SEC	Self-Evident Corrections
SmPC	Summary of product characteristics
SOP	Standard Operating Procedure
SPC	Supplementary Protection Certificate
SUSAR	Suspected Unexpected Serious Adverse Reaction
TMF	Trial Master File

Synopsis

**Study title**	**Heterologous vaccination with an ChAdOx1-S prime and a BNT162b2 boost**
**Investigational Product**	**ChAdOx1-S** [COVID-19 Vaccine AstraZeneca suspension for injection COVID-19 Vaccine (ChAdOx1-S [recombinant])]<br>**BNT162b2** [Comirnaty concentrate for dispersion for injection COVID-19 mRNA Vaccine (nucleoside modified)]
**Indication**	ChAdOx1-S and BNT162b2 are intended for immunization for SARS-CoV-2
**Design of clinical trial**	Multi-centre, single-blinded, three-arm, randomized clinical trial<br>After interim analysis one arm is closed and the study is continued as multi-centre, non-blinded, two-arm, non-randomized clinical trial
**Number of trial sites**	4
**Duration of clinical trial / Timetable**	**Information concerning the clinical trial:**<br>Time of recruitment:<br>Planned start (FPFV): May 2021<br>Planned end of trial (LPLV): November 2022<br>Information concerning subjects:<br>Duration of treatment: Single (boost) vaccination as part of this clinical trial (Prime shot had already been administered before study entry – inclusion criterion), after day 90 non-responders are offered a third immunization, all other participants are offered a third immunization after day 180
**Objectives (Primary/Secondary)**	*Primary objectives:*<br>1. To determine whether the immune response to vaccine SARS-CoV-2 spike protein is at least as effective for the heterologous prime-boost regime Vaxzevria followed by Comirnaty as in the approved homologous Vaxzevria and Comirnaty regimen.<br>2. To determine immune responses in non-responders after a third immunization<br>3. To determine immune responses all participants after a third immunization<br><br>*Secondary objective:*<br>1. Potentially show clinical efficacy against infection, especially with B.1.351, B.1.1.7+E484K and other immune escape variants<br>2. To analyze in detail safety and tolerability after the second vaccination especially in the heterologous arm. (clinical, lab)<br>3. To evaluate the clinical course and outcome of COVID-19 diseases after vaccination with ChAdOx1-S prime & boost vs. ChAdOx1-S prime and BNT162b2 boost vs BNT162b2 prime & boost<br>4. To analyze safety and tolerability after the 3rd dose vaccination (clinical, lab)

	5. To analyze T-cell responses in non-responders after 3rd immunization<br>6. Potentially show clinical efficacy against infection, especially with B.1.351, B.1.1.7+E484K and other immune escape variants after 3rd immunization
**Endpoints (Primary/Secondary)**	Primary endpoints:<br>1a Level of neutralizing antibodies against wild-type and immune escape variants in the 3 study groups at 10, 30, 90 and 180 days post boost.<br>1b Level of neutralizing antibodies against wild-type and immune escape variants at 10, 30, 90 and 180 days post 3rd immunization.<br><br>*Secondary endpoints:*<br>1. Level of T cell responses against SARS-CoV-2 spike protein peptides pools per subgroup at 30 days.<br>2. Occurrence of breakthrough infection with wild type, B.1.351 and other immune escape variants of SARS-CoV-2<br>3. Occurrence of adverse events, serious adverse events and adverse events of special interest<br>4. The clinical course (hospitalization, ICU admission) and outcome of COVID-19 diseases (recovery, long-Covid mortality).<br>5. Occurrence of adverse events, serious adverse events and adverse events of special interest after 3rd immunization<br>6. Level of T cell responses against SARS-CoV-2 spike protein peptides pools in non-responders after 3rd immunization<br>7. Occurrence of breakthrough infection with wild type, B.1.351 and other immune escape variants of SARS-CoV-2 after 3rd immunization
**Planned number of subjects**	N=2124 (n=124 have already been randomized into the homologous Vaxzevria arm, up to 1000 participants will be included in each of the other two arms)
**In- and exclusion criteria**	**Inclusion criteria:**<br>1. Subject provides written informed consent<br>2. Participant is ≥ 18 and ≤ 65 years of age on the day of signing the ICF<br>3. Individuals that are eligible for vaccination according to the Austrian vaccination plan.<br>4. Participants that have been vaccinated with either ChAdOx1-S prime within the last 12 weeks or BNT162b2 prime within the last 3 – 6 weeks<br>5. Subject understands and agrees to comply with study procedures<br>6. Subject must be willing to be contacted by telephone or willing to complete an eDiary during study participation<br>7. Female participants of childbearing potential may be enrolled in the study if the participant fulfills all the following criteria:<br>- has a negative urine pregnancy test at screening

	- has agreed to practice adequate contraception from providing consent until 3 months after administration of study vaccine<br>- is not currently breastfeeding<br><br>Adequate female contraception is defined as consistent and correct use of an approved contraceptive method, for example:<br>• Barrier method (condoms, diaphragm, cervical cap) used in conjunction with spermicide<br>• Prescription hormonal contraceptive taken administered via oral (pill), transdermal (patch), subdermal or IM route<br>• Intrauterine device<br>• Sterilization of a female participant`s monogamous male partner prior to study inclusion<br>Cave: periodical abstinence (eg calendar, ovulation, symptothermal,...) and withdrawal are not acceptable methods of contraception.<br><br>8. Female participants of non-childbearing potential may be enrolled in the study. Non-childbearing potential is defined as: surgically sterile (history of bilateral dubal ligation, bilateral oophorectomy, hysterectomy) or postmenopausal (amenorrhea for ≥ 12 consecutive months prior to Screening without an alternative medical cause).<br>9. Participants agrees to not donate bone marrow, blood and blood products from the study vaccine administration until 3 months after receiving the study vaccine<br><br>**Inclusion criteria for 3rd immunization:**<br><br>1. Subjects that have received the second dose of vaccination within this study.<br><br>2. No neutralizing antibodies on day 90 post second dose (only for participants that will receive the third dose between day 110 and 180). All other participants will be offered a third dose between day 200 and 260 after the second dose, independent of their antibody titers.<br>3. Subject provides written informed consent<br>4. Subject understands and agrees to comply with study procedures<br>5. Subject must be willing to be contacted by telephone or willing to complete an eDiary during study participation<br>6. Female participants of childbearing potential may be enrolled in the study if the participant fulfills all the following criteria:

	- has a negative urine pregnancy test at screening<br>- has agreed to practise adequate contraception from providing consent until 3 months after administration of study vaccine<br>- is not currently breastfeeding<br><br>Adequate female contraception is defined as consistent and correct use of an approved contraceptive method, for example:<br>• Barrier method (condoms, diaphragm, cervical cap) used in conjunction with spermicide<br>• Prescription hormonal contraceptive taken administered via oral (pill), transdermal (patch), subdermal or IM route<br>• Intrauterine device<br>• Sterilization of a female participant`s monogamous male partner prior to study inclusion<br>Cave: periodical abstinence (eg calendar, ovulation, symptothermal,...) and withdrawal are not acceptable methods of contraception.<br>7. Female participants of non-childbearing potential may be enrolled in the study. Non-childbearing potential is defined as: surgically sterile (history of bilateral dubal ligation, bilateral oophorectomy, hysterectomy) or postmenopausal (amenorrhea for ≥ 12 consecutive months prior to Screening without an alternative medical cause).<br>8. Participants agrees to not donate bone marrow, blood and blood products from the study vaccine administration until 3 months after receiving the study vaccine<br><br>**Exclusion criteria:**<br>1. Participant has already received full vaccination against SARS-CoV-2<br>2. Prior administration of an investigational coronavirus (SARS-CoV, MERS-CoV) vaccine or current/planned simultaneous participation in another interventional study to either prevent or treat COVID-19<br>3. Participant has received/plans to receive a non-study vaccine within 14 days prior to or after any dose of IP<br>4. Participant has a contraindication to IM injections and blood draws (eg, bleeding disorders)<br>5. Participants has a known or suspected allergy or history of anaphylaxis, urticaria or other significant adverse reactions to vaccines or their excipients (including specifically the excipients of the study vaccine; refer to the IB)

	6. Subjects with previous positive PCR-test result for SARS-CoV-2 or positive anti-SARS-CoV-2 N protein antibody test<br>7. History of leukemia, lymphoma, or underlying bone marrow disorder (eg, myelodysplasia, myeloma, myeloproliferative disorder) or history of bone marrow transplant.<br>8. Malignancy that required treatment with chemotherapy, immunotherapy, radiation therapy, or other antineoplastic target therapies within 24 months prior to study enrollment.<br>9. Has participated in an interventional clinical study within 30 days prior to study inclusion<br><br>Subjects are excluded from the third dose of vaccination if any of the following criteria apply in addition to the above mentioned ones:<br>1. Participant already received a third dose of vaccination.<br>2. Subjects with positive PCR-test result for SARS-CoV-2 after study enrolment or a positive anti-SARS-CoV-2 N antibody test on day 180.
**Methodology of the study**	Prospective controlled randomized study on vaccination against SARS-CoV-2; after an interim analysis the study is continued as a non-randomized study. Participants that have already been enrolled will be followed until day 180. New participants are only enrolled into Group A and Group B.<br>Dosage BNT162b2: 30µg/0.3ml/injection<br>Dosage ChAdOx1-S: 5x10^{10} viral particles /0.5ml/ injection<br>The study includes 3 arms:<br>Group A (randomized): ChAdOx1-S prime and BNT162b2 boost12 weeks (± 1 week) between prime and boost. Until the interim analysis this group had been randomized. Following interim analysis new participants will be enrolled in a non-randomized, non-blinded fashion.<br>Group B (non-randomized): 2 Doses of BNT162b2, 4 - 6 weeks between prime and boost<br>Group C (randomized): 2 Doses of ChAdOx1-S, 12 weeks (± 1 week) between prime and boost. Recruitment of new participants is discontinued for this group after interim analysis.
**Statistical methods & analyses**	An interim analyses applying the Haybittle Peto rule for early stopping [1] had been applied. Safety and efficacy have been analyzed on day 30 after n=200 patients had been randomized. level of neutralizing antibodies (primary endpoint) had been significantly lower in the homologous Vaxzevria arm compared to the heterologous (p-value < 0.001) this arm had been stopped. Already recruited participants in all arms will be further followed for durability of antibody response and the number of breakthrough infections. New participants in the two remaining arms are recruited with the aim to recruit up to 1000 participants in each of these two arms to analyse the number of breakthrough infections. Efficacy analysis will employ contingency table analysis,

	chi-square testing and a logistic regression adjusting for the stratifying variables study site and sex.

Visit Plan after boost vaccination

Subjects with previous prime shot		Visit I (Study enrollment & day of boost shot)	Visit II Day 1 after boost shot	Visit III Day 2 after boost shot	Visit IV - VIII Day 3 – Day 7 after boost shot	Visit IX Day 10 after boost shot	Visit VIII, X – XXXIII every 7 days until Day 182 after boost shot	AV1, AV2, AV3 on Day 30, 90, 180 after boost shot
Visit window	- 7 to -3 days	0	0	0	0	± 1 day	± 2 day	± 3 day
Written Informed Consent	X							
Inclusion & Exclusion criteria	X							
Demographic data	X							
Medical History/Comorbidities	X							
Physical examination (incl. vital signs)	X			X		X		
Urine Pregnancy test	X							
Laboratory[3]				X		X		
Immunogenicity blood draw	X[1, 2]					X[2]		X[2]
Concomitant vaccinations	x							
Concomitant medication	X	X	X	X	X	X		
AEs and SAEs		X	X	X	X	X	X	X
Vaccination		X						
Post-vaccination surveillance – eDiary or phone calls							X	
Antigen-testing						X[4]		
PCR-testing / Antigen-testing		X[7]				X[5]	X[5]	
Antibodies	X							
Post vaccination observation		X[6]						
ediary, symptom reporting			x		x			

[1] Before vaccination;
[2] Assessment of antibody against wt and immune escape variants,
[3] Blood count, coagulation status, CRP, etc.
[4] Refer to COVID-19 disease observation schedule
[5] In case of positive Antigen-test
[6] Participants will be closely observed for at least 30 minutes post-vaccination to monitor for the development of acute reactions. Participants will be allowed to leave the study site after it is documented that the post-vaccination observation period is complete.

[7] not older than 72 hours before vaccination, alternatively: registered antigen test (not older than 24 hours before vaccination), participants will additionally give a sample for a PCR test at visit I prior to vaccination

Visit Plan after 3rd vaccination

Subjects with previous prime/boost vaccination an no/low neutralizing antibodies	**Visit I (Study enrollment & day of 3rd vaccination)**	**Visit II - VIII Day 1 – 7 after 3rd vaccination**			**Visit IX Day 10 after 3rd vaccination**	**Visit VIII, X – XXXIII every 7 days until Day 182 after 3rd vaccination**	**AV1, AV2, AV3 on Day 30, 90, 180 after 3rd vaccination**
Visit window	**Day 110-180 or 200-260 after boost shot**	**0**			**± 1 day**	**± 2 day**	**± 3 day**
Written Informed Consent	X						
Inclusion & Exclusion criteria	X						
Demographic data	X						
Medical History/Comorbidities	X						
Physical examination (incl. vital signs)	X				X		
Urine Pregnancy test	X						
Laboratory[3]					X		
Immunogenicity blood draw					X[2]		X[2]
Concomitant vaccinations							
Concomitant medication	X	X			X		
AEs and SAEs	X	X			X	X	X
Vaccination	X						
Post-vaccination surveillance – eDiary or phone calls						X	
Antigen-testing					X[4]		
PCR-testing / Antigen-testing	X[7]				X[5]	X[5]	
Antibodies							
Post vaccination observation	X[6]						
ediary, symptom reporting		x					

[1] Before vaccination;
[2] Assessment of antibody against wt and immune escape variants,
[3] Blood count, coagulation status, CRP, etc.
[4] Refer to COVID-19 disease observation schedule
[5] In case of positive Antigen-test

[6] Participants will be closely observed for at least 30 minutes post-vaccination to monitor for the development of acute reactions. Participants will be allowed to leave the study site after it is documented that the post-vaccination observation period is complete.

[7] not older than 72 hours before vaccination, alternatively: registered antigen test (not older than 24 hours before vaccination), participants will additionally give a sample for a PCR test at visit I prior to vaccination

Visit Plan: COVID-19 disease observation schedule

The following table/schedule contains all interventions and examinations planned during the clinical trial in case a PCR-test for SARS-CoV-2 is positive. Further and more detailed lines will be listed in the respective sections of this CIP (e.g. in case of medical errors or premature termination of the trial).

	**Observation Start Day 0**	**Call 1 – 10 every 3 days until second negative PCR-test**
Baseline evaluation	X	
Concomitant medication	X	
AEs and SAEs	X	X
PCR-test and Virus isolation		X
Phone call or eDiary		X

1. Introduction

1.1 Study Background

After one year and despite massive non-pharmaceutical interventions, the COVID-19 pandemic is still not under control. The only way out seems to be an effective vaccination program. However, immune escape variants are evolving in several regions worldwide. Infection with the wild-type SARS-CoV-2 and several vaccines developed so far do not effectively protect against infection with these immune escape variants. ChAdOx1-S, e.g., was shown to have very little efficacy against the immune escape variant B.1.351. However, the mRNA based vaccine BNT162b2 developed by BioNTech/Pfizer induces higher levels of cross neutralizing antibodies than the ChAdOx1-S vector vaccine developed by AstraZeneca and was shown to cross-protect against immune escape variants quite well.[2]

1.2 Preclinical and Clinical Data

Several studies have shown that individuals that have been infected with the SARS-CoV-2 virus have a potent T-cell memory and high levels of neutralizing antibodies for up to 8 months[3]. Reports on reinfections have been rare. In the past few months, however, immune escape variants have emerged first in South Africa (B.1.351) and Brazil (P1). Also in the UK, Denmark, the Czech Republic, and India variants with immune escape mutations or a higher infectivity have emerged[4]. These have multiple mutations in the receptor binding site and the N- terminus of the SARS-CoV-2 surface protein S. Sera from convalescent individuals neutralize these new variants inefficiently.

Only one year after the start of the COVID 19 pandemic three highly effective vaccines are already in use in Europe, with additional vaccines used regionally and several vaccines that are expected to be approved within the next months. Two mRNA vaccines, BNT162b2 and mRNA-1273, developed by BioNTech/Pfizer and Moderna, respectively, are currently approved[5-7]. These have an efficacy of over 90% not only in phase III clinical trials but also during the vaccine program as reported in Israel [8]. The vector vaccine ChAdOx1-S is also very effective with around 85% protection.

Sera from vaccinated and convalescent individuals neutralize the wild-type SARS-CoV-2 very efficiently, but the immune escape variants are inhibited on average appr. 5-10fold less effectively. While the drop in efficacy against B.1.351 was found to be similar for the sera from ChAdOx1-S and BNT162b2 vaccinated individuals, titers after the mRNA vaccine were 3-4 fold higher against wild-type and the immune escape variants. This is in accordance with a small clinical trial, where hardly any efficacy of ChAdOx1-S against B.1.351 was found[9] . Similarly, in larger studies in South Africa, a protein vaccine from Novavax and the vector vaccine from J&J were found to have a reduced efficacy against B.1.351 infection. However, the J&J vaccine was effective in preventing severe disease with a similar efficacy of 85% for wild-type virus and for B.1.351. Thus it is likely that although ChAdOx1-S cannot effectively prevent infection with this immune escape variant it can prevent severe disease and thus reduce the mortality[10].

Potentially, the efficacy of ChAdOx1-S is not optimal, because the same vector is used for prime and boost. Vaccinees develop an immune response to the adenovirus vector after the first vaccinations, which, for the boost vaccination, limits vector cell entry and the length of antigen expression by neutralizing antibodies and T cells, respectively. Thus, it also seems likely that further booster vaccinations with ChAdOx1-S adapted to the immune escape variants will be less effective in individuals that have received their primary immunization with ChAdOx1-S. However, the immunity to the vector wanes over time and this issue is not yet clearly resolved.

1.3 Need of the Study

The immune escape variants are spreading worldwide. Local transmission of the B.1.351 is observed in several European regions while P1 cases are mostly still travel associated. In Austria, there is local transmission of the Czech variant B.1.258Δ in several regions, while the P1 variant from Brazil plays no role yet. However, B.1.351 local transmission is observed in Tyrol, where 20-30% of SARS-CoV-2 infections were caused by this immune escape variant in January/February 2021, but then controlled but not eliminated by intensive testing. In March and April the largest world-wide outbreak of B.1.1.7 + E484K, also an immune escape variant, was observed in Tyrol. Although, some of the diagnosed cases seemed to be false positive for the E484K mutation, still several hundred have currently already been confirmed, a number that is internationally still unprecedented. It is expected that until the winter infection season, that is likely to hit Europe after a phase of low-level infections in summer, the immune escape variants will make up a substantial portion of SARS-CoV-2 infections in the countries with high seroprevalence after the vaccine campaigns such as Austria. Therefore, the vaccine programs must build up an effective immunity against the wild-type virus as well as the immune escape variants.

ChAdOx1-S contributes considerably to the arsenal of vaccines purchased by the European Community. During this study we already recruited approximately 300 participants. So far, 100 for the homologous Vaxzevria group, 106 for the heterologous Comirnaty prime/Vaxzevria boost group and 13 for the homologous Comirnaty group reached day 30 and were analyzed for safety and efficacy. The data of this interim analysis has been evaluated by the Data Safety Monitoring Board, which recommended a discontinuation of the homologous Vaxzevria arm due to significantly inferior immune responses. However, the study needs to be continued to analyze durability of immune responses in the already recruited participants. Additionally, more participants will be recruited to be able to compare as already initially planned the number of breakthrough infections between the homologous Comirnaty and the heterologous group.

First reports from Israel indicate that durability of vaccine protection after two Comirnaty doses might be limited[11]. We therefore plan to compare the level and durability of cross-neutralizing antibodies induced by classical BNT162b2 and ChAdOx1-S vaccinations with the heterologous prime and boost with ChAdOx1-S followed by BNT162b2, respectively.

While analyzing neutralizing antibody responses on day 30 in the participants that had already been recruited for our study we observed a number of participants with no or only

very low titers of neutralizing antibodies against non-immune escape virus variants. The number of non-responders was even higher for immune escape virus variants such as B.1.351. These non-responders were mainly found in the homologous Vaxzevria group (2 against all three analyzed virus variants (B.1.1.7, B.1.351 and B.1.617.2), further 3 against B.1.351 and B.1.617.2 and additional 14 only against B.1.351). Only one person in the heterologous group did not develop neutralizing antibodies against B.1.617.2 but high titers of neutralizing antibodies against the other two variants. The "Nationales Impfgremium" in Austria recently recommended a third vaccination for non-responders of a complete vaccination scheme starting 4 weeks after the second immunization. Therefore, we plan to apply a third dose with Comirnaty in participants that do not have neutralizing antibodies against the wild-type variant on day 90, which will be applied between day 110 and 180.

The "Nationales Impfgremium" in Austria recommends a third vaccination in fully vaccinated persons either 6-9 or 9-12 months after completion of the regular vaccine scheme depending on risk factors and the type of vaccine used for the basic immunization. We therefore plan to offer all participants that received the second vaccination during this study and were followed until day 180 a third dose with Comirnaty. This third dose will especially be recommended for participants with no or low levels (<1:64) of cross-neutralizing antibodies against B.1.351. We will analyze immune responses, especially cross-neutralizing antibodies after the third dose.

1.4 Risk-Benefit Analysis

Both vaccines used in this trial are licensed. We have seen in the already recruited participants that the mixed vaccine regimen with ChAdOx1-S prime followed by BNT162b2 boost shows similar side effects as the two homologous arms. There is not yet data available on the reactogenicity of a third vaccination. However, we do not assume that side effects of a Comirnaty vaccination after two Vaxzevria doses are much different than after one Vaxzevria dose and the likely benefit for world-wide vaccine programs in controlling the SARS-CoV-2 variants would be enormous, as described above.

2. Study Objectives and Endpoints

2.1 Primary Study Objectives and Endpoints

Primary Objectives	Primary Endpoints
**Efficacy Objective**<br>To determine whether the immune response to vaccine SARS-CoV-2 spike protein is at least as effective for the heterologous prime-boost regime Vaxzevria followed by Comirnaty as in the approved	**Efficacy Endpoint**<br>1a Level of neutralizing antibodies against wild-type and immune escape variants in the 3 study groups at 10, 30, 90 and 180 days post boost.

homologous Vaxzevria and Comirnaty regimen.<br><br>To determine immune responses in non-responders after a third immunization<br><br>To determine immune responses all participants after a third immunization	1b Level of neutralizing antibodies against wild-type and immune escape variants at 10, 30, 90 and 180 days post 3rd immunization.

2.2 Secondary Study Objectives and Endpoints

Secondary Objectives	Secondary Endpoints
**Efficacy Objective**<br>To determine whether the immune response to vaccine SARS-CoV-2 spike protein is at least as effective for the heterologous prime-boost regime Vaxzevria followed by Comirnaty as in the approved homologous Vaxzevria and Comirnaty regimen.<br><br>To analyze T-cell responses in non-responders after 3rd immunization	Level of T cell responses against SARS-CoV-2 spike protein peptides pools per subgroup at 30 days<br><br>Level of T cell responses against SARS-CoV-2 spike protein peptides pools in non-responders after 3rd immunization
**Efficacy Objective**<br>Potentially show clinical efficacy against infection, especially with B.1.351, B.1.1.7+E484K and other immune escape variants<br><br>Potentially show clinical efficacy against infection, especially with B.1.351, B.1.1.7+E484K and other immune escape variants after 3rd immunization	**Efficacy Endpoint**<br>• Occurrence of breakthrough infection with wild type, B.1.351 and other immune escape variants of SARS-CoV-2<br><br>Occurrence of breakthrough infection with wild type, B.1.351 and other immune escape variants of SARS-CoV-2 after 3rd immunization
**Safety Objective**<br>To analyze in detail safety and tolerability after the second vaccination. (clinical, lab)<br><br>To analyze safety and tolerability after the 3rd dose vaccination (clinical, lab)	**Safety Endpoint**<br>• Occurrence of adverse events, serious adverse events and adverse events of special interest<br><br>Occurrence of adverse events, serious adverse events and adverse events of special interest after 3rd immunization

**Efficacy Objective**	**Efficacy Endpoint**
To evaluate the clinical course and outcome of COVID-19 diseases after vaccination with ChAdOx1-S prime & boost vs. ChAdOx1-S prime and BNT162b2 boost	The clinical course (hospitalization, ICU admission) and outcome of COVID-19 diseases (recovery, long-Covid mortality)

3. Study Design

3.1 Study Description

This is a Phase II, multi-centre, single blinded, three-arm, randomized clinical trial to determine the level of neutralizing antibodies against wild-type and immune escape variants in the 3 study groups as well as the level of T cell response against peptide pools from wild-type in adults aged ≥ 18. After discontinuation of the homologous Vaxzevria arm participants that have already been vaccinated are followed for 180 days after vaccination as described below. New participants will be recruited in a non-blinded and non-randomized fashion for the two remaining arms (homologous Comirnaty and heterologous arm). Participants from this second recruitment phase will also be followed initially planned.
In an extension of the study a third Comirnaty dose will be offered to participants with no neutralizing antibodies on day 90 or no cross-neutralizing antibodies on day 180. The additional third dose will be applied between day 110 and 180 or day 200 and 260, respectively.

3.2 Timetable

Following information has to be included within this section:

- start of recruitment: May 2021
- planned duration of recruitment phase: 6 months
- duration of vaccination phase and follow-up phase per patient: 6 months
- follow-up phase of patients after third vaccination: 6 months
- planned start date of clinical trial (FPFV): May 2021
- estimated end of clinical trial (LPLV): December 2022

4. Study Population

The following eligibility criteria are designed to select subjects for whom protocol treatment is considered appropriate. All relevant medical and nonmedical conditions should be taken into consideration when deciding whether this protocol is suitable for a particular subject.

4.1 Number of Patients

A total of 2124 subjects will be included in this study. Each arm of 1000 subjects will be vaccinated as it follows:

Group A: ChAdOx1-S prime and BNT162b2 boost
Group B: BNT162b2 boost prime and BNT162b2 boost
Group C: ChAdOx1-S prime and ChAdOx1-S boost (this arm has been closed after 124 subjects have been randomized to; therefore, the total number of subjects is reduced from 3000 to 2124).

4.2 Inclusion Criteria

Subjects are eligible to be included in the study only if all the following criteria apply:

1. Subject provides written informed consent
2. Participant is ≥ 18 and ≤ 65 years of age on the day of signing the ICF
3. Individuals that are eligible for vaccination according to the Austrian vaccination plan.
4. Participants that have been vaccinated with either ChAdOx1-S prime within the last 12 weeks or BNT162b2 prime within the last 3 – 6
5. Subject understands and agrees to comply with study procedures
6. Subject must be willing to be contacted by telephone or willing to complete an eDiary during study participation
7. Female participants of childbearing potential may be enrolled in the study if the participant fulfills all the following criteria:

- has a negative urine pregnancy test at screening
- has agreed to practise adequate contraception from providing consent until 3 months after administration of study vaccine
- is not currently breastfeeding

Adequate female contraception is defined as consistent and correct use of an approved contraceptive method, for example:

- Barrier method (condoms, diaphragm, cervical cap) used in conjunction with spermicide
- Prescription hormonal contraceptive taken administered via oral (pill), transdermal (patch), subdermal or IM route
- Intrauterine device

- Sterilization of a female participant`s monogamous male partner prior to study inclusion

Cave: periodical abstinence (eg calendar, ovulation, symptothermal,...) and withdrawal are not acceptable methods of contraception.

8. Female participants of non-childbearing potential may be enrolled in the study. Non-childbearing potential is defined as: surgically sterile (history of bilateral dubal ligation, bilateral oophorectomy, hysterectomy) or postmenopausal (amenorrhea for ≥ 12 consecutive months prior to Screening without an alternative medical cause).
9. Participants agrees to not donate bone marrow, blood and blood products from the study vaccine administration until 3 months after receiving the study vaccine

Subjects are eligible to be included for a third vaccination only if all the following criteria apply:

9. Subjects that have received the second dose of vaccination within this study.

10. No neutralizing antibodies on day 90 post second dose (only for participants that will reveive the third dose between day 110 and 180. All other participants will be offered a third dose between day 200 and 260 after the second dose, independent of their antibody titers.
11. Subject provides written informed consent
12. Subject understands and agrees to comply with study procedures
13. Subject must be willing to be contacted by telephone or willing to complete an eDiary during study participation
14. Female participants of childbearing potential may be enrolled in the study if the participant fulfills all the following criteria:

- has a negative urine pregnancy test at screening
- has agreed to practise adequate contraception from providing consent until 3 months after administration of study vaccine
- is not currently breastfeeding

Adequate female contraception is defined as consistent and correct use of an approved contraceptive method, for example:

- Barrier method (condoms, diaphragm, cervical cap) used in conjunction with spermicide
- Prescription hormonal contraceptive taken administered via oral (pill), transdermal (patch), subdermal or IM route
- Intrauterine device
- Sterilization of a female participant`s monogamous male partner prior to study inclusion

Cave: periodical abstinence (eg calendar, ovulation, symptothermal,...) and withdrawal are not acceptable methods of contraception.

15. Female participants of non-childbearing potential may be enrolled in the study. Non-childbearing potential is defined as: surgically sterile (history of bilateral dubal ligation, bilateral oophorectomy, hysterectomy) or postmenopausal (amenorrhea for ≥ 12 consecutive months prior to Screening without an alternative medical cause).

16. Participants agrees to not donate bone marrow, blood and blood products from the study vaccine administration until 3 months after receiving the study vaccine

4.3 Exclusion Criteria

Subjects are excluded from the interventional part of the study if any of the following criteria apply:

1. Participant has already received full vaccination against SARS-CoV-2
2. Prior administration of an investigational coronavirus (SARS-CoV, MERS-CoV) vaccine or current/planned simultaneous participation in another interventional study to either prevent or treat COVID-19
3. Participant has received/plans to receive a non-study vaccine within 14 days prior to or after any dose of IP (except for seasonal influenza vaccine which is not permitted within x days before or after any dose of IP)
4. Participant has a contraindication to IM injections and blood draws (eg, bleeding disorders)
5. Participants has a known or suspected allergy or history of anaphylaxis, urticaria or other significant adverse reactions to vaccines or their excipients (including specifically the excipients of the study vaccine; refer to the IB)
6. Subjects with previous positive PCR-test result for SARS-CoV-2 or positive anti-SARS-CoV-2 N antibody test
7. History of leukemia, lymphoma, or underlying bone marrow disorder (eg, myelodysplasia, myeloma, myeloproliferative disorder) or history of bone marrow transplant.
8. Malignancy that required treatment with chemotherapy, immunotherapy, radiation therapy, or other antineoplastic target therapies within 24 months prior to study enrollment.
9. Has participated in an interventional clinical study within 30 days prior to study inclusion

Subjects are excluded from the third dose of vaccination if any of the following criteria apply in addition to the above mentioned ones:

3. Participant already received a third dose of vaccination.
4. Subjects with positive PCR-test result for SARS-CoV-2 after study enrolment or a positive anti-SARS-CoV-2 N antibody test on day 180.

4.4 Withdrawal of study subjects after study start/Discontinuation of Study Treatment

Subjects have the right to withdraw fully or partially from the study at any time and for any reason without prejudice to their future medical care by the physician or at the institution.

Withdrawal of full consent for a study means that the subject does not wish to receive further investigational treatment and does not wish or is unable to continue further study participation. Subject data up to withdrawal of consent will be included in the subject's study data, but no further information will be collected unless a separate consent has been given.

Withdrawal of partial consent means that the subject does not wish to take protocol-specific product(s) any longer but is still willing to collaborate in providing further data by continuing on study.
Should a subject request or decide to withdraw from the study, all efforts will be made to complete and report the observations as thoroughly as possible up to the end of withdrawal.

For any patient who has received IMP and withdrew prematurely from the study every effort should be made for attendance of a safety follow-up visit. The primary reason for withdrawal from the study should be documented. Patients will not be followed for any reason after consent has been withdrawn, unless a separate consent has been given for further data collection.

The investigator has the right to discontinue a patient from IMP or withdraw a patient from the study at any time. The primary reason for withdrawal from the study should be documented.

The investigator may withhold a participant from the boost shot if he/she experiences any of the following:
-becomes pregnant
-develops symptoms or conditions listed in the exclusion criteria
experiences an AE afater dosing that is considered by the investigator to be related to IP and is of Grade 3 (severe) or higher
-experiences an AE/SAE that, considered by the Investigator, requires IP withdrawal due to its nature, severity or required treatment, regardless of any causal relationship to vaccine

-shows a clinically significant vital sign measurement or finding on physical examination that, in the judgement of the investigator, requires IP withdrawal

If patient discontinues study participation for reasons unrelated to an adverse event, immunization of the patient will be continued according to the recommendations of the Austrian NIG (Nationales Impfgremium). No additional patient will be enrolled to replace that subject.

Reasons for removal investigational treatment or observation might include:

- Withdrawal of consent
- Administrative decision by the investigator
- Pregnancy
- Significant protocol deviation
- Subject noncompliance
- Serious Adverse event
- Other safety concern of the investigator or sponsor
- Death
- Lost to follow-up (participant repeatedly fails to return for scheduled visits without stating an intention to withdraw consent & he/she cannot be contacted by the study site after 3 contact attempts-these contact attempts must be documented in the participant`s medical record)

Participants that had been recruited during the first part of the study when this has still been conducted as a blinded and randomized study might request premature unblinding. In that case, participants might be kept part of the study after unblinding and will be further followed regarding durability of immune responses and breakthrough infections until day 180. If eligible, these participants might be included in a third vaccination.

5. Study Procedures

5.1 General Study Procedures and Assessments Schedule

The person obtaining consent must be suitably qualified and experienced, and have been delegated this duty by the CI/PI on the delegation log.

It is the responsibility of the investigator, or a person delegated by the investigator to obtain written informed consent from each subject prior to participation in the trial, following adequate explanation of the aims, methods, anticipated benefits and potential hazards of the study. The investigator or designee will explain the patients that they are under no

obligation to enter the trial and that they can withdraw at any time during the trial, without having to give a reason.

A copy of the signed informed consent form will be given to the participant. The original signed and dated form will be retained at the study site.

If new safety information results in significant changes in the risk/benefit assessment, the consent form will be reviewed and updated if necessary and subjects will be re-consented as appropriate.

5.2 Screening Visit (Day - 7 to -3)

All subjects must provide written informed consent before any study-specific assessments or procedures are performed.

Only subjects fulfilling all the inclusion and none of the exclusion criteria will be accepted in the study.

The following study screening assessments are to be completed prior to study entry:

- Physical examination including vital signs
- Pregnancy test (in case the previous test was performed more than 21 days before), in case of a positive pregnancy test the subject will be excluded from the study
- Blood draw:

  9 ml EDTA for antibody testing
- Concomitant medication
- Concomitant vaccination

Please note screening does not necessarily constitute enrolment. Screening failures i.e. patients who do not meet eligibility criteria at the time of screening may be eligible for rescreening at a later timepoint upon the decision of the Sponsor.

5.3 Visit I (Day 0, Day of Boost shot)

All subjects who have already received **ChAdOx1-S** as prime shot will receive the second dose with **Comirnaty (BNT162b2)**.

Subjects who have received **Comirnaty (BNT162b2)** as prime will be vaccinated again with **Comirnaty (BNT162b2)**.

Before IMP injection the following assessment should be performed:

- SARS-CoV-2 PCR Test (not older than 72 hours before vaccination)
- Alternatively: registered antigen test (not older than 24 hours before vaccination), participants will additionally give a sample for a PCR test at visit I prior to vaccination, in case this PCR test is positive the participant will be excluded after vaccination

After IMP injection AEs the subject will be observed for 30 minutes and all AEs/SAEs will be recorded.

Participants should seek immediate medical attention if they develop symptoms such as

- shortness of breath
- chest pain
- leg swelling
- persistent abdominal pain following vaccination
- neurological symptoms including severe or persistent headaches and blurred vision
- skin bruising (petechial) beyond the site of vaccination after a few days

5.4 Visit III (Day 2 after boost shot)

The following assessments will be performed on Visit III:

- Physical examination including vital signs
- Blood draw
    - Coagulation (PT INR, antithrombin III, fibrinogen, D-Dimer, APTT)
    - CRP
    - Blood chemistry (albumin, alkaline phosphatase, aspartate aminotransferase (AST), alanine aminotransferase (ALT), lactate dehydrogenase (LDH), Gamma-GT, creatinine, uric acid, total bilirubin, total protein, blood urea nitrogen)
    - Hematology (Hematocrit, Hemoglobin, platelet count, WBC, RBC)
- AEs/SAEs
- Concomitant medication

5.5 Visit II, IV - VIII (Day 1, 3 – 7 after boost shot)

Subjects have to report information on reactogenicity on Day 1 and between Day 3 and Day 7 after boost shot. This information will be reported using an eDiary or via telephone call. The following information will be collected:

- pain at injection site
- erythema (redness) at injection site
- swelling/induration (hardness) at injection site
- localized axillary swelling or tenderness ipsilateral to the injection arm
- Headache
- Fatigue
- myalgia (muscle aches all over the body)
- arthralgia (aching in several joints)
- nausea/vomiting
- body temperature (potentially fever)
- chills
- other AEs

5.6 Visit IX (Day 10 ± 1 day)

The following assessments will be performed on Visit IX:

- Physical examination including vital signs
- Blood draw
    - Coagulation (PT INR, antithrombin III, fibrinogen, D-Dimer, APTT)
    - CRP
    - Blood chemistry (albumin, alkaline phosphatase, aspartate aminotransferase (AST), alanine aminotransferase (ALT), lactate dehydrogenase (LDH), Gamma-GT, creatinine, uric acid, total bilirubin, total protein, blood urea nitrogen)
    - Hematology (Hematocrit, Hemoglobin, platelet count, WBC, RBC)
- AEs/SAEs

- Immunogenicity blood draw:

  9 ml EDTA for antibody testing
  Up to 45 ml EDTA/heparin for T-cell analysis

5.7 Visit VIII, X – XXXIII (every 7 days ± 2 day after boost shot)

Every 7 days (± 2) after boost shot until Day 182 subjects have to undergo antigen or RT-PCR testing. If this test confirms an infection with SARS-CoV-2, the clinical course and outcome will be followed. Virus from swabs will be isolated and sequenced and further analyzed for immune escape.
In addition, subjects complete their reports in the eDiary or via telephone call.

5.8 Additional visits on Day 30 (AV 1), Day 90 (AV 2) and Day 180 (AV 3) after boost shot (± 3 day)

The following examinations will be performed:

- Physical examination including vital signs
- Blood draw:

  9 ml EDTA for antibody testing
  Up to 45 ml EDTA/heparin for T-cell analysis

- Concomitant medication
- Concomitant vaccination
- AEs/SAEs

5.9 Unscheduled Visits

Unscheduled visits will not be integrated in the study except when they are related to a positive SARS-CoV-2 RT-PCR result or an adverse event.

5.10 Study Assessments

5.10.1 Physical Examination

All physical examinations will be performed by trained medical personnel only.
A full physical examination including vital signs, height and BMI/weight (only at screening), will be performed according to the time points in the visit schedule. Symptom-directed physical examinations may be performed at other time-points at the discretion of the Investigator. The full examination includes assessment of skin, head, ears, eyes, nose, throat,

neck, thyroid, lungs, heart, cardiovascular, abdomen, lymph nodes and musculoskeletal system/extremities.
Heart rate and blood pressure will be measured after the patient has been in a sitting position for at least 5 minutes.

On Day0 vital sign measurements will be collected once before IP injection and 30 minutes after IP injection before participants are discharged from study site.
In case of blood collection, vital sign measurements should be performed before.
Febrile Participants at day 0 (body temperature ≥ 38.0 °C) may be rescheduled within the relevant window periods.

If any of the vital sign measurements is out of range and clinically significant at the discretion of the Investigator, the abnormal value will be documented on the AE page of the eCRF.

5.10.2 SARS-CoV-2 RT-PCR-testing

Weekly antigen or RT-PCR tests have to be performed starting on Day 7 until Day 182 after boost shot.

5.10.3 Surveillance of clinical course and outcome of SARS-CoV2 infection

In case of a positive SARS-CoV-2 RT-PCR test the subject will be followed until end of quarantine and full recovery.
The following information will be collected for subjects in outpatient care:

- Fever (temperature ≥ 38ºC) or chills (of any duration, including ≤ 48 hours)
- Shortness of breath or difficulty breathing (of any duration, including ≤ 48 hours)
- Cough (of any duration, including ≤ 48 hours)
- Fatigue
- Muscle or body aches
- Headache
- New loss of taste or smell
- Sore throat
- Congestion or runny nose
- Nausea or vomiting
- Diarrhea
- Symptoms of long-Covid

Additional information must be collected for hospitalized subjects and infection will be defined according to the WHO score for COVID-19 (ordinal scale for Clinical Improvement)

- Clinical signs indicative of severe systemic illness, Respiratory Rate ≥ 30 per minute, Heart Rate ≥ 125 beats per minute, SpO2 ≤ 93% on room air at sea level or PaO2/FIO2 < 300 mm Hg, OR
- Respiratory failure or Acute Respiratory Distress Syndrome (ARDS), (defined as needing high-flow oxygen, non-invasive or mechanical ventilation, or ECMO), evidence of shock (systolic blood pressure < 90 mmHg, diastolic BP < 60 mmHg or requiring vasopressors), OR
- Significant acute renal, hepatic or neurologic dysfunction, OR
- Admission to an intensive care unit or death

5.10.4 Blood sampling volumes

The maximum planned volumes of blood sampled per subject are

	**Screening visit**	**Visit III (Day 2)**	**Visit IX (Day 10)**	**AV1, AV2, AV3 on Day 30, 90, 180 after boost shot**
Visit window	**-7 to -3**	**0**	**± 1 day**	**± 3 day**
Immunogenicity blood samples	9 ml EDTA		9 ml EDTA<br>up to45 ml EDTA/heparin	9 ml EDTA<br>up to 45 ml EDTA/heparin
Other lab values		Clinical chemistry and blood count<br>3 ml citrate<br>5.5 ml heparin<br>2.7 ml EDTA	Clinical chemistry and blood count<br>3 ml citrate<br>5.5 ml heparin<br>2.7 ml EDTA	

5.11 Third dose off vaccination

Participants that are eligible to a third vaccine dose as described above due to a lack of neutralizing antibody response on day 90 might be enrolled for the third vaccination between day 110 and 180. All other participants that have been followed till day 180 might be enrolled for a third vaccination between day 200 and 260.

5.12 Visit I – 3rd vaccination

All subjects will receive the third dose with **Comirnaty (BNT162b2)**.

Before IMP injection the following assessment should be performed:

- SARS-CoV-2 PCR Test (not older than 72 hours before vaccination)

- Alternatively: registered antigen test (not older than 24 hours before vaccination), participants will additionally give a sample for a PCR test at visit I prior to vaccination, in case this PCR test is positive the participant will be excluded after vaccination

After IMP injection AEs the subject will be observed for 30 minutes and all AEs/SAEs will be recorded.

Participants should seek immediate medical attention if they develop symptoms such as
- shortness of breath
- chest pain
- leg swelling
- persistent abdominal pain following vaccination
- neurological symptoms including severe or persistent headaches and blurred vision
- skin bruising (petechial) beyond the site of vaccination after a few days

5.13 Visit II - VIII (Day 1 – 7 after 3rd vaccination)

Subjects have to report information on reactogenicity between Day 1 and Day 7 after 3rd vaccination. This information will be reported using an eDiary or via telephone call. The following information will be collected:

- pain at injection site
- erythema (redness) at injection site
- swelling/induration (hardness) at injection site
- localized axillary swelling or tenderness ipsilateral to the injection arm
- Headache
- Fatigue
- myalgia (muscle aches all over the body)
- arthralgia (aching in several joints)
- nausea/vomiting
- body temperature (potentially fever)
- chills
- other AEs

5.14 Visit IX (Day 10 ± 1 day after 3rd vaccination)

The following assessments will be performed on Visit IX:

- Physical examination including vital signs
- Blood draw
    - Coagulation (PT INR, antithrombin III, fibrinogen, D-Dimer, APTT)
    - CRP
    - Blood chemistry (albumin, alkaline phosphatase, aspartate aminotransferase (AST), alanine aminotransferase (ALT), lactate dehydrogenase (LDH), Gamma-GT, creatinine, uric acid, total bilirubin, total protein, blood urea nitrogen)
- Hematology (Hematocrit, Hemoglobin, platelet count, WBC, RBC)
- AEs/SAEs
- Immunogenicity blood draw:

  9 ml EDTA for antibody testing
  Up to 45 ml EDTA/heparin for T-cell analysis

5.15 Visit VIII, X – XXXIII (every 7 days ± 2 day after 3rd vaccination)

Every 7 days (± 2) after 3rd vaccination until Day 182 subjects have to undergo antigen or RT-PCR testing. If this test confirms an infection with SARS-CoV-2, the clinical course and outcome will be followed. Virus from swabs will be isolated and sequenced and further analyzed for immune escape.
In addition, subjects complete their reports in the eDiary or via telephone call.

5.16 Additional visits on Day 30 (AV 1), Day 90 (AV 2) and Day 180 (AV 3) after 3rd vaccination (± 3 day)

The following examinations will be performed:

- Physical examination including vital signs
- Blood draw:

  9 ml EDTA for antibody testing
  Up to 45 ml EDTA/heparin for T-cell analysis

- Concomitant medication
- Concomitant vaccination

- AEs/SAEs

5.17 Unscheduled Visits after 3rd vaccination

Unscheduled visits will not be integrated in the study except when they are related to a positive SARS-CoV-2 RT-PCR result or an adverse event.

5.18 Study Assessments after 3rd vaccination

See study assessments after boost vaccination above. The following derivations to the procedures described above are applied after the 3rd vaccination: no lab values on day 2 and 10; all participants will be analyzed for T cell responses on day 10, 30, 90 and 180.

5.19 Discontinuation of the Study/Premature Termination of the Trial

The sponsor has the right to discontinue a participating site in this trial at any time. Reasons for discontinuing site participation may include, but are not limited to the following:

- patient enrolment is unsatisfactory
- administrative reasons

The sponsor will notify the investigator if the sponsor decides to discontinue the participation of the site.
The sponsor has the right to terminate the trial prematurely at any time, due to any relevant medical or ethical concerns. The reasons for terminating the trial must be documented in detail. All trial subjects still under treatment at the time of termination must undergo a final examination which must be documented. The principal coordinating investigator must be informed without delay if any investigator has ethical concerns about continuation of the trial.

Premature termination of the trial will be considered if:

- the risk-benefit balance for the trial subjects changes markedly
- it is no longer ethical to continue treatment with the IMP
- the sponsor considers that the trial must be discontinued for safety reasons (e.g. advice of the DSMB)
- An interim analysis or results of other research show that one of the trial treatments is superior or inferior to another
- it is no longer practicable to complete the trial

The sponsor decides about discontinuation of the trial in consultation with the PCI, DMC and/or statistician.
In case of discontinuation of the trial the IEC and competent regulatory authority must be informed within 15 days about early termination.

5.20 Closure of the Study

The clinical trial ends when the last subject has completed the planned last visit according to protocol.
The completion of the study will be reported to the ethics committee as well as to the relevant authorities.

6. Study Medication (IMP)

6.1 Vaxzevria Suspension for injection and Comirnaty concentrate for dispersion for injection

Vaxzevria is a chimpanzee adenovirus encoding the SARS-CoV-2 Spike glycoprotein (ChAdOx1-S) and is produced in genetically modified human embryonic kidney (HEK) 293 cells by recombinant DNA technology. Vaxzevria is indicated for active immunization to prevent COVID-19 caused by SARS-CoV-2, in individuals 18 years of age or older.

Comirnaty is a single-stranded, 5´-capped messenger RNA (mRNA) produced using a cell-free in vitro transcription from the corresponding DNA templates encoding the viral spike (S) protein of SARS-CoV-2. Comirnaty is indicated for active immunization to prevend COVID-19 caused by SARS-CoV-2 virus, in individuals 16 years of age and older.

6.2 Dosage and Administration

In general a complete vaccination course consists of two separate doses.
Participants who have already been vaccinated with Comirnaty (0.3 ml) will receive a second dose of Cormirnaty (0.3 ml) 4 – 6 weeks after prime vaccination to complete the vaccination series. (Group B)

Those participants who have already been vaccinated with Vaxzevria (ChAdOx1-S) will be randomized to Group A (boost shot with Comirnaty [BNT162b] – 0.3 ml) or Group C (boost shot with Vaxzevria [ChAdOx1-S – 0.5 ml]).

In any case the second dose should be administered 12 weeks (± 1 week) after the first dose with Vaxzevria (ChAdOx1-S).

The second dose of Vaxzevria is regularly recommended after 4-12 weeks, whereby studies favor the longer interval and the NIG also advises the longer interval. As the proportion of Vaxzevria in the vaccine portfolio in Europe is declining, more individuals can be recruited if a longer interval for the Vaxzevria prevaccinated is chosen. A second reason is that thereby the homologous Vaxzevria arm has the same vaccination interval as the Vaxzevria-Comirnaty arm. Finally, the Com-Cov Study in Oxford is currently comparing Vaxzevria-Cormirnaty heterologous vaccination with a 4 week and a 12-week interval. These data will be available to complement the data in our study.

6.3 Dose Modifications

No dose modification is allowed in this study.

6.4 Concomitant Medication

Prophylactic administration of antipyretic/analgesic drugs, started at the time of immunization and repeated 6 and 12 hours later is not recommended.

Antipyretic/analgesic drugs may be taken to treat postimmunization fever and irritabilities.

In case of vaccine reactions the following medical management is recommended:

REACTION	SIGNS AND SYMPTOMS	MANAGEMENT
Localized	Soreness, redness, itching, or swelling at the injection site	Apply a cold compress to the injection site. Consider giving an analgesic (pain reliever) or antipruritic (anti-itch) medication.
	Slight bleeding	Apply pressure and an adhesive compress over the injection site.
	Continuous bleeding	Place thick layer of gauze pads over site and maintain direct and firm pressure; raise the bleed-ing injection site (e.g., arm) above the level of the patient's heart.
Psychological fright, presyncope, and syncope (fainting)	Participant feels "faint" (e.g., light-headed, dizzy, weak, nauseated, or has visual disturbance)	Have participant lie flat. Loosen any tight clothing and maintain open airway. Apply cool, damp cloth to patient's face and neck. Keep them under close observation until full recovery.
	Fall, without loss of consciousness	Examine the participant to determine if injury is present before attempting to move the participant. Place patient flat on back with feet elevated.

	Loss of consciousness	Check to determine if injury is present before attempting to move the participant. Place patient flat on back with feet elevated. Participant should be transferred to hospital ambulance for further examinations.
Anaphylaxis	Skin and mucosal symptoms such as general-ized hives, itching, or flushing; swelling of lips, face, throat, or eyes. Respiratory symptomssuch as nasal congestion, change in voice, sensation of throat closing, stridor, shortness of breath, wheeze, or cough. Gastrointestinal symptoms such as nausea, vomiting, diarrhea, cramping abdominal pain. Cardiovascular symptoms such as collapse, dizziness, tachycardia, hypotension.	Follow the recommendation of treating anaphylaxis.

Recommendation of treating anaphylaxis

1. If itching and swelling are confined to the injection site where the vaccination was given, observe patient closely for the development of generalized symptoms.
2. If symptoms are generalized, participant has to be transferred to the next hospital. This should be done by a second person, while the primary healthcare professional assesses the airway, breathing, circulation, and level of consciousness of the patient. Vital signs should be monitored continuously.
3. DRUG DOSING INFORMATION: The first-line and most important therapy in anaphylaxis is epinephrine. There are NO absolute contraindications to epinephrine in the setting of anaphylaxis.
    a. First-line treatment: Epinephrine is the first-line treatment for anaphylaxis, and there is no known equivalent substitute. Use epinephrine in a 1.0 mg/mL aqueous solution (1:1000 dilution). Administer a 0.3 mg dose IM using a premeasured or prefilled syringe or an autoinjector in the mid-outer thigh. If using another epinephrine formulation, the recommended dose is 0.01 mg/kg,ranging for adults from 0.3 mg to maximum dose of 0.5 mg. Administer

IM, preferably in the mid-outer thigh. Epinephrine dose may be repeated 2 addi-tional times every 5–15 minutes (or sooner as needed) while waiting for EMS to arrive.

   b. Optional treatment: H1 antihistaminesrelieve itching and urticaria (hives). These medications DO NOT relieve upper or lower airway obstruction, hypo-tension, or shock. Consider giving diphenhydramine (e.g., Benadryl) for relief of itching and hives. Administer orally 1–2 mg/kg every 4–6 hours, up to a maximum single dose of 100 mg.

4. Monitor the patient closely until EMS arrives. Perform cardiopulmonary resusci-tation (CPR), if necessary, and maintain airway. Keep patient in recumbent posi-tion (flat on back) unless he or she is having breathing difficulty. If breathing is difficult, patient's head may be elevated, provided blood pressure is adequate to prevent loss of consciousness. If blood pressure is low, elevate legs. Monitor blood pressure and pulse every 5 minutes.
5. Record the patient's reaction (e.g., hives, anaphylaxis) to the vaccine, all vital signs, medications administered to the patient, including the time, dosage, response, and the name of the medical personnel who administered the medi-cation, and other relevant clinical information.
6. Notify the sponsor according to section

6.5 Labelling

As all IMPs hold a marketing authorization no additional manufacturing steps are applied. There is no need of a specific labelling.

6.6 Handling of IMP at the Site and Drug Accountability

The investigator ensures that the IMP is only used according to this protocol. The investigator is responsible for the drug accountability log. Drug accountability checked by a monitor during site visits and at the completion of the trial.

6.7 Subject Compliance

IMP will be administered by study physician and the prime shot has already been administered outside the study (before informed consent).

6.8 Blinding and Unblinding

All groups have already received the prime vaccination. The two groups that have received a prime with Vaxzevria (ChAdOx1-S) will be randomized into a group that receive again Vaxzevria (ChAdOx1-S) or Comirnaty (BNT162b2). The administration is blinded. The participants only are not informed in which arm they were randomized.

The syringes with the vaccines will be prepared in a separate room and brought into the vaccination room immediately before application. The unblinding will take place on day 90 post boost.

All participants recruited after closing of the homologous Vaxzevria arm that have received the prime immunization with Vaxzevria will receive the boost vaccination with Comirnaty in a non-blinded fashion.

All participants who have already received a prime with Comirnaty (BNT162b2) will receive Comirnaty (BNT162b2) again.

7. Adverse Events

7.1 Summary of known and possible Adverse Events of the IMP

Adverse events observed for BNT162b2 (Comirnaty)

From EMA product information: https://www.ema.europa.eu/en/documents/product-information/comirnaty-epar-product-information_en.pdf

Table 1: Adverse reactions from Comirnaty clinical trials and post-authorisation experience

System Organ Class	Very common (≥ 1/10)	Common (≥ 1/100 to < 1/10)	Uncommon (≥ 1/1,000 to < 1/100)	Rare (≥ 1/10,000 to < 1/1,000)	Not known (cannot be estimated from the available data)
Blood and lymphatic system disorders			Lymphadenopathy		
Immune system disorders			Hypersensitivity reactions (e.g. rash, pruritus, urticaria,[a] angioedema[a])		Anaphylaxis
Psychiatric disorders			Insomnia		
Nervous system disorders	Headache			Acute peripheral facial paralysis[b]	
Gastrointestinal disorders	Diarrhoea[c]	Nausea; Vomiting[c]			
Musculoskeletal and connective tissue disorders	Arthralgia; Myalgia		Pain in extremity[d]		
General disorders and administration site conditions	Injection site pain; Fatigue; Chills; Pyrexia;[e] Injection site swelling	Injection site redness	Malaise; Injection site pruritus		

a. The frequency category for urticaria and angioedema was Rare.
b. Through the clinical trial safety follow-up period to 14 November 2020, acute peripheral facial paralysis (or palsy) was reported by four participants in the COVID-19 mRNA Vaccine group. Onset was Day 37 after Dose 1 (participant did not receive Dose 2) and Days 3, 9, and 48 after Dose 2. No cases of acute peripheral facial paralysis (or palsy) were reported in the placebo group.
c. Adverse reaction determined post-authorisation.
d. Refers to vaccinated arm.
e. A higher frequency of pyrexia was observed after the second dose.

Adverse events reported for ChAdOx1-S

From https://www.ema.europa.eu/en/documents/product-information/covid-19-vaccine-astrazeneca-product-information-approved-chmp-29-january-2021-pending-endorsement_en.pdf

Table 1 Adverse drug reactions

MedDRA SOC	Frequency	Adverse Reactions
Blood and lymphatic system disorders	Uncommon	Lymphadenopathy
Metabolism and nutrition disorders	Uncommon	Decreased appetite
Nervous system disorders	Very common	Headache
	Uncommon	Dizziness<br>Somnolence
Gastrointestinal disorders	Very common	Nausea
	Common	Vomiting<br>Diarrhoea
Skin and subcutaneous tissue disorders	Uncommon	Hyperhidrosis<br>Pruritus<br>Rash
Musculoskeletal and connective tissue disorders	Very common	Myalgia<br>Arthralgia
General disorders and administration site conditions	Very common	Injection site tenderness<br>Injection site pain<br>Injection site warmth<br>Injection site pruritus<br>Injection site bruising[a]<br>Fatigue<br>Malaise<br>Feverishness<br>Chills
	Common	Injection site swelling<br>Injection site erythema<br>Fever[b]

[a] Injection site bruising includes injection site haematoma (uncommon)
[b] Measured fever ≥38°C

Participants randomized to Arm A (Vaxzevria prime followed by Comirnaty boost) may suffer from a combination of the adverse events listed above.

Additionally the following “adverse event of special interests” should be followed closely in all 3 arms:

Thrombosis with Thrombocytopenia Syndrome (TTS)/Vaccine-induced Thrombotic Thrombocytopenia (VITT)

TTS/VITT is caused by antibodies that recognize platelet factor 4 (PF4, also called CXCL4) bound to platelets. These antibodies are immunoglobulins (Ig) that activate platelets via low affinity platelet FcγIIa receptors (receptors on the platelet surface that bind the Fc portion of IgG).

Urgent medical evaluation for TTS/VITT is indicated if any of the following develops after vaccination:
- Severe headache
- Visual changes

- Abdominal pain
- Nausea and vomiting
- Back pain
- Shortness of breath
- Leg pain or swelling

Petechiae, easy bruising, or bleeding

Many individuals are hospitalized due to the severity of their clinical condition. An exception may be an individual with isolated thrombocytopenia (without thrombosis) who can be treated with a direct oral anticoagulant (DOAC) with very close follow-up.

As an initial indicator for potential TTS, platelets will be counted in the blood sampled on day 10.

If the slightest indication for TTS/VITT arises from clinical symptoms or low platelet counts, the individual will be immediately referred to the Department for Internal Medicine V, at the University Hospital Innsbruck, to the coagulation disorder specialists.

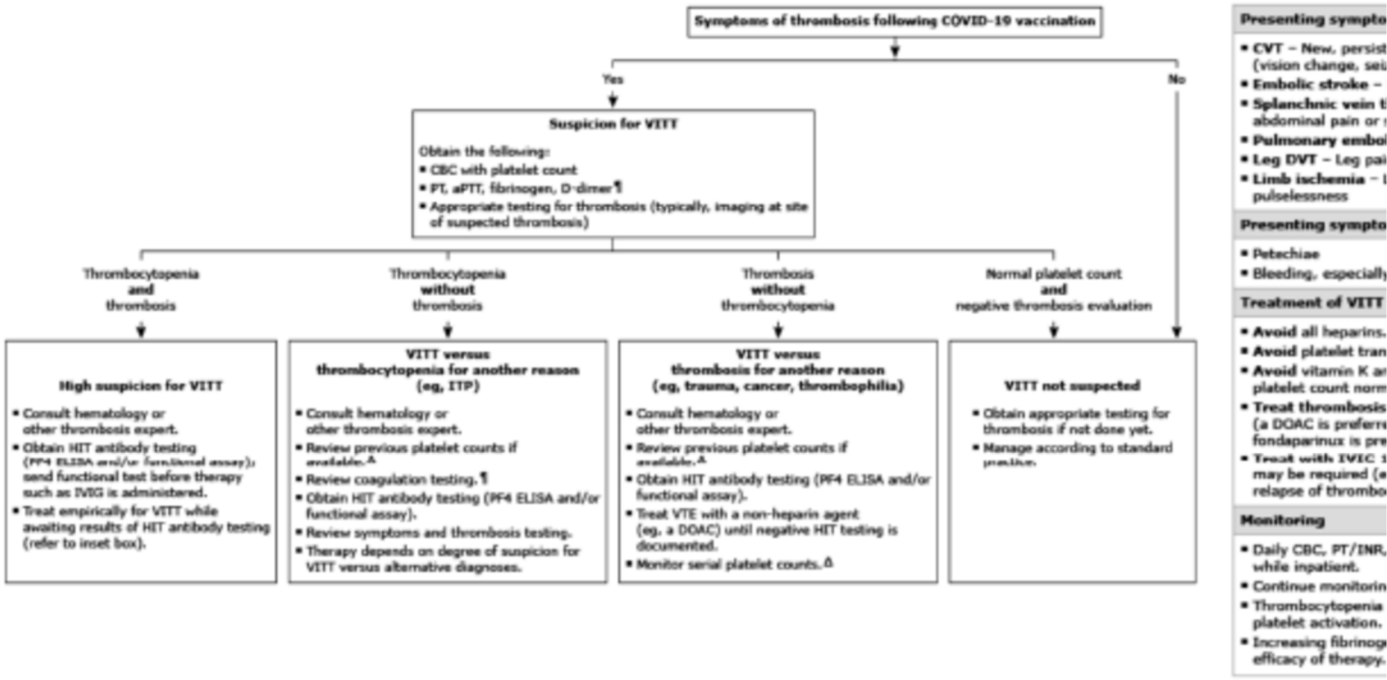

7.2 Adverse Events

An adverse event (AE) is any untoward adverse change from the subject's baseline condition, i.e. any unfavorable and unintended sign including an abnormal laboratory finding, symptom or disease which is considered to be clinically relevant by the physician that occurs during the course of the study, whether or not considered related to the study drug.
Adverse events include:

- worsening or increase in frequency or intensity of a pre-existing disease or medical condition
- abnormal laboratory tests

Adverse events do not include:

- pre-planned interventions/hospitalizations
- medical or surgical procedures, e.g. surgery, endoscopy, tooth extraction, transfusion. however, the event leading to the procedure is an AE. If this event is serious, the procedure must be described in the SAE narrative.
- pre-existing disease or medical condition that does not worsen

- overdose of either study drug or concomitant medication without any signs or symptoms

Lack of efficacy (vaccine failure)

Lack of efficacy (vaccine failure) is defined as 2 consecutive positive tests for SARS-CoV-2 within a week (2 PCRs OR one antigen test + one PCR), independent of potential symptoms. These cases will be reported and documented as Serious Adverse Reactions ("lack of efficacy"). A higher frequency in the combination regimen (Arm A) as would be expected based on the efficacy profile of Comirnaty® or Vaxzevria® will be reported to the BASG as SUSAR.
In case of infection in vaccinated individuals, the cases will be reported to the authorities, incl. mutation analysis, advised to stay at home and if symptomatic treated according to the guide lines (Covid-19 guide lines). If these vaccine failures occur significantly more frequently in the heterologous than in the homologous arm, which is extremely unlikely based on previous experience with heterologous vaccination regimes containing adenovirus vectors, the study will immediately be terminated. An amendment to the study plan will be submitted. If predictors for vaccine failures in the heterologous arm can be determined (low neutralizing antibody levels), individuals with this predictor will receive a 2nd Comirnaty vaccination and followed up.

7.3 Serious Adverse Events

If a serious adverse event (SAE) occurs, the investigator must alert the sponsor without unjustified delay to any AE (whether causally or not) from this study that results in one of the following outcomes, or is significant for any other reason:

- death (excluding death from progressive disease)
- a life-threatening experience – refers to an event in which the subject was at risk of death at the time of the event; it does not refer to an event which hypothetically might have caused death if it were more severe
- initial hospitalization, or prolongation of existing inpatients' hospitalization
- persistent or significant disability or incapacity
- congenital anomaly or birth defect

Addtionally all Thrombosis with Thrombocytopenia Syndrome (TTS)/Vaccine-induced Thrombotic Thrombocytopenia (VITT) will be classified as serious adverse event.

Medical judgment should be exercised in deciding whether an AE/adverse drug reaction (ADR) is serious in other situations. Important AE/ADRs that are not immediately life-threatening or do not result in death or hospitalization but may jeopardize the subject or

may require intervention to prevent one of the other outcomes listed in the definition above, should also be considered serious.
Patients should be closely observed for adverse events while receiving treatment with the IMP. Further on for 12 weeks after discontinuation from study therapy in order to detect delayed toxicity. After this period, the clinical trial medical safety desk will only be alerted to serious adverse events if the investigator believes that the event may have been caused by the investigational device or by a protocol procedure.
Events not considered to be serious adverse events are hospitalizations for the:

- routine treatment or monitoring of the studied indication, not associated with any deterioration in condition
- treatment, which was elective or pre-planned, for a pre-existing condition that did not worsen
- treatment on emergency, outpatient basis for an event not fulfilling any of the definitions of serious given above and not resulting in hospital admission.

Disease progression will not be reported as AE or SAE unless the progression is unexpected in severity or early occurrence. Progressive disease will be appropriately documented on the case report form as part of the efficacy parameters.

The study will specifically pay attention to known potential serious adverse events of special interest (AESI) describe for COVID-19 vaccines, which are non-hemorrhagic stroke, hemorrhagic stroke, acute myocardial infarction, deep vein thrombosis, pulmonary embolism, anaphylaxis, Bell’s palsy, myocarditis/pericarditis, narcolepsy, appendicitis, immune thrombocytopenia, disseminated intravascular coagulation, encephalomyelitis (including acute disseminated encephalomyelitis), Guillain-Barre syndrome, and transverse myelitis.

7.4 Adverse Drug Reaction (ADR) & Unexpected Adverse Drug Reaction

Noxious and unintended responses to a IMP related to any dose should be considered adverse drug reactions. The phrase responses to a medicinal product means that a causal relationship between a medicinal product and an adverse event is at least a reasonable possibility, i.e. the relationship cannot be ruled out.
Regarding marketed medicinal products: a response to a drug which is noxious and unintended and which occurs at doses normally used in man for prophylaxis, diagnosis, or therapy of diseases or for modification of physiological function (see the ICH Guideline for Clinical Safety Data Management: Definitions and Standards for Expedited Reporting).

7.5 Suspected Unexpected Serious Adverse Reaction (SUSAR)

SUSARs are serious adverse reactions with a suspected causal relationship to the study drug that is unexpected (not previously described the SmPC or IB) and serious.

7.6 Pregnancy

Any pregnancy that occurs during study participation must be reported to the investigator/sponsor immediately. If a pregnancy should be confirmed after informed consent has been obtained but prior to the initiation of the study drug, the patient must be excluded from the trial.
The pregnancy must be followed up to determine outcome (including premature termination) and status of mother and child. Pregnancy complications (including spontaneous abortions) and elective terminations must be reported as an AE or SAE.
Any SAE occurring in association with a pregnancy brought to the investigator's attention after the subject has completed the study and considered by the investigator as possibly related to the IMP, must be immediately reported to the sponsor.
As the consent for the study does not cover the consent for the follow-up of the pregnancy, a separate consent has to be obtained.

7.7 Grading of Severity of Adverse Events

Intensity of all adverse events will be graded according to the NCI common terminology criteria for adverse events (CTCAE), version 6.0 on a five-point scale (grade 1 to 5) and reported in detail in the CRF.
Adverse events not listed in the CTCAE version xxx should be graded as follows:

**CTC grade**	**Equivalent to**	**Definition**
Grade 1	mild	Discomfort noticed but no disruption of normal daily activity
Grade 2	moderate	Discomfort sufficient to reduce or affect daily activity; no treatment or medical intervention is indicated although this could improve the overall wellbeing or symptoms of the patient.
Grade 3	severe	Inability to work or perform normal daily activity; treatment or medical intervention is indicated in order to improve the overall wellbeing or symptoms; delaying the onset of treatment is not putting the survival of the patient at direct risk.
Grade 4	life-threatening /disabling	An immediate threat to life or leading to a permanent mental or physical condition that prevents work or performing normal daily activities; treatment or medical intervention is required in order to maintain survival.
Grade 5	death	AE resulting in death

Mild, moderate as well as severe adverse events do not need to be serious events. Those definitions are commonly used for describing the intensity of a concrete event (e.g.: mild, moderate and severe myocardial infarction). A serious adverse event with a relatively small medical importance (e.g.: strong headache) does not necessarily need to be a severe event. For example: Nausea for several hours can be indicated as a severe adverse event, without being clinically significant. Fever with 39°C won't be graded as severe event; however, if the fever delays the discharge from hospital, it can be graded as serious adverse event.
The severity of an adverse event serves as a guideline for the regulatory reporting obligation.

7.8 Causality

For all, the investigator will assess the causal relationship between the IMP and the AE using his/her clinical expertise and judgment according to the following algorithm that best fits the circumstances of the AE:

Unrelated

- May or may not follow a reasonable temporal sequence from administration of the IMP
- Is biologically implausible and does not follow known response pattern to the suspect IMP (if response pattern is previously known)
- can be explained by the known characteristics of the subject's clinical state or other modes of medication administered to the subject.

Unlikely

- There is a reasonable temporal relation between the AE and the IMP, but there is a plausible other explanation for the occurrence of the AE possibly.
- Follows a reasonable temporal sequence from administration of the IMP. The AE may equally be explained by the study subject's clinically state, environmental or toxic factors, or concomitant therapy administered to the study subject.
- The relationship between the medical device and AE may also be clinically plausible.

Probably

- Follows a reasonable temporal sequence from administration of the IMP, and plausible reasons point to a causal relation with the IMP.

Related

- Follows a reasonable temporal sequence form administration of the medical device.
- Follows a known response pattern to the medical device (if response pattern is previously known).
- No other reasonable cause is present.

Not assessable

- The causal relationship between the IMP and the AE cannot be judged.

7.9 Reporting Procedures

7.9.1 Reporting Procedures for Adverse Events (AEs)

A special section is designated to adverse events in the CRF where the following details must be entered:

- type of adverse event (diagnosis or syndrome; if not known signs or symptoms)

- start (date)
- end (date)
- severity (mild, moderate, severe, life-threatening/disabling, death)
- serious (no / yes)
- unexpected (no / yes)
- outcome (resolved, ongoing, ongoing – improved, ongoing – worsening)
- action taken (none, study medication dose reduced, study medication interrupted, study medication discontinued, medication therapy, surgical procedure, hospitalization, other)
- relation to study drug (possibly, probably or definitely related or unlikely, probably not related or definitely not related)

Certain events require immediate reporting to allow the sponsor to take appropriate measures to address potential new risks in a clinical trial. The investigator must report such events to the sponsor immediately; The reporting has to take place within 24 hours after notification. Regardless of relationship to study drug the investigator has to follow within 24 hours of notification:

- Serious adverse events
- Pregnancies

The investigator has to report immediately new significant follow-up information resulting from follow up examination to the sponsor (i.e. no more than 24 hours after becoming aware of the information). New significant information includes the following:

- new signs or symptoms or a change diagnosis
- new significant new diagnostic test results
- change in causality based on new information
- change in the event's outcome, including recovery
- additional narrative information on the clinical course of the event

Investigators must also comply with local requirements for reporting SAEs to the local health authority and IRB/EC

7.9.2 Reporting Procedures for SAEs and SUSARs

Investigators be asked to determine for possible AEs. All AEs, whether reported by the patient or noted by study personnel, will be recorded in the patient's medical record and on the Adverse Event eCRF.
After informed consent has been obtained but prior to initiation of the study drug, only SAEs considered to be related to a protocol-mandated intervention should be reported (e.g. SAEs related to invasive procedures such as biopsies).

After initiation of study drug, all AEs and SAEs regardless of relationship to the study drug will be reported until 7 days or until the event has resolved to baseline grade or better the event is assessed as stable by the investigator the patient is lost to follow-up or the patient withdraws consent.
After 8 days, investigators should report any deaths, SAEs, or other AEs of concern that are believed to be related to prior treatment with the study drug.
Any Serious Adverse Event (SAE) that is ongoing at the time of 7 days should be followed-up until resolved.

In the case of a serious adverse event, the investigator has to use all supportive measures for best patient treatment. The SAE form must be completed by the investigator and reported no more than 24 hours after awareness of the event.
The following details should be available with the initial report:

- patient number
- patient: date of birth, sex
- name of investigator and trial site
- period of administration
- the suspected investigational medicinal product (IMP)
- the adverse event assessed as serious
- concomitant disease and medication
- relevant medical history
- short description of the event and outcome
- description
    - onset and end (if applicable)
    - therapeutic intervention
    - causal relationship to each of the (study drugs)
    - hospitalization or prolongation of hospitalization
    - death, life-threatening, persistent or significant disability or incapacity

If applicable, the initial report should be followed by the follow-up report, indicating the outcome of the SAE.
For SAEs, SUSARs and pregnancies, the sponsor or a designee may follow up by telephone, fax, electronic mail, and/or a monitoring visit to obtain additional case details and outcome information (e.g. from hospital discharge summaries, consultant reports, autopsy reports) in order to perform an independent medical assessment of the reported case. A follow-up SAE form must also be completed and reported appropriately.
SUSARs will be reported to the required regulatory authorities, investigators/institutions, and ethical committees in compliance with all reporting requirements according to local regulations and good clinical practice by the sponsor and/or its designees.

Once per year, the sponsor or PCI will supply a report on the safety of trial subjects with all available relevant information concerning patient safety during the reference period to the competent supreme and the competent authorities of all other member states of the EU or EEA where the trial is being conducted. This report will also be supplied to the responsible ethics committee.
The annual safety report will be compiled according to the corresponding ICH guideline E2F „Development Safety Update Report – DSUR“
The data lock point for the patient data to be included and analyzed is the day of the approval of the clinical trial.
Detailed requirements regarding form and content are covered by the ICH Guideline E2F „Development Safety Update Report – DSUR“. The DSUR presents a comprehensive analysis of the current safety profile concerning the study drug.
The sponsor or PCI will supply the report within 60 days of one year after the reference date (data-lock point).
The data lock point is the last day of the one-year reporting period following the "Development International Birth Date" (DIBD). This date is the sponsor's first authorization to conduct a clinical trial in any country worldwide. When the DBID is not available to the sponsor, the data lock point can be defined as the first authorization to conduct a clinical trial in the EU if an adequate explanation can be provided.

8. Documentation

The conduct of the study in agreement with the GCP-guidelines and the investigational plan as well as the accuracy of all data documented in the CRF are the responsibility of the investigator. All collected data of this study have to be recorded on the CRF by appropriate authorized persons. This also includes data of patients who dropped-out of the study.
The investigator records the participation on a special identification list of patients. This list gives the possibility for a later identification of the patients and contains the patient number, full name, date of birth and the date of the enrollment into the study. The identification list of patients remains in the study center after the closure of the study.
Additionally, the participation of the patient in this clinical study has to be recorded in the patient`s medical record (investigational medicinal product, number of patient or randomization, start and end of the study).
Further it has to be assured that all persons authorized for CRF entries can be identified. A list with signatures and identification codes of the persons must be archived in the ISF and TMF. Furthermore logs according to ICH E6 (e.g. Signature/Delegation/Screening/Drug Accountability log) will be implemented and maintained by the Investigator.

8.1 Data Recording (CRF/eCRF)

8.2 Trial Folders

The trial folders should contain the complete documentation of the trial. They should allow the evaluation of the conducted trial and data quality.

8.2.1 Trial Master File (TMF)

The paper-based/electronic TMF, established at the beginning of the trial and secured in a safe place, contains all essential documents that demonstrate that the trial is conducted in accordance with regulatory requirements and ICH GCP. All documents will be maintained and updated as appropriate throughout the trial. Previous versions of the documents must be retained in the TMF and will be clearly labelled as outdated or will be relocated in a section for outdated documents. The TMF is archived at the end of the study for 15 years.

8.2.2 Investigator Site File (ISF)

The paper-based/electronic ISF, established at the beginning of the trial will be secured in a safe place (the file is provided to the site at the site initiation visit). It contains all essential documents maintained by the PI(s). All documents will be maintained and updated as appropriate throughout the trial. Previous versions of the documents must be retained in the ISF and will be clearly labelled as outdated or versions will be relocated in a section for outdated documents. Within the Monitoring, the ISF will be checking regarding actuality and completeness in accordance with the formalities. After completion or discontinuation of the study this ISF has to be kept for 15 years.

8.3 Data Storage

8.3.1 Storage duties of the Sponsor

The Sponsor has to keep all study-relevant documents of the completed or discontinued clinical trial after completion or discontinuation of the study for a minimum of 15 years.

8.3.2 Storage duties of the Investigator

The Investigators have to keep all records and documents, which are related with the study or the allocation of investigational medicinal products (e.g. data entry form, consent form, list of the allocations of investigational medicinal products and further relevant documents), for a minimum of 15 years.
Medical records and other original data have to be kept for 30 years.

9. Data Management

The protocol has been reviewed from a database designing perspective, for clarity and consistency. The entries made in the CRF will be monitored by the Clinical Monitor for completeness and filled up CRFs are retrieved and handed over to the CDM.

Discrepancies are either flagged to the investigator for clarification or closed in-house by Self-Evident Correction (SEC) in case of obvious spelling errors. For discrepancies that require clarification from the investigator, Data Clarification Forms (DCFs) will be sent to site. Investigators will write the resolution or explain the circumstances that led to the discrepancy

in data within a given time period. When a resolution is provided by the investigator, the same will be updated in the database by the CDM. The resolved data discrepancies are recorded as „closed“. In cases, where the investigator will not be able to provide a resolution for the discrepancy, they will be considered as „irresolvable“ and will be updated in the discrepancy database.

10. Protocol Deviations

All deviations to the study protocol have to be documented with an explanation. Deviations have to be reported to the sponsor, who is responsible for their valuation.
Reasons for trial termination of a patient have to be documented. In case of termination for safety reasons or insufficient effect of the IMP, the patient has to be further monitored.
Deviations have to be analyzed whether changes of the clinical investigation plan or the closure of the study are necessary.
If necessary, the ethics committee or the responsible authorities have to be informed.
Please define instructions for documentation, report and analysis of protocol deviations.
Requirements for reporting including time frame.
Corrective and preventive measures and criteria for the exclusion of a principal investigator.

11. Statistics

11.1 Sample Size

The sample size is generated for the primary endpoint of a superiority testing of level of neutralizing antibodies against the B.1.351variant between the two randomized study arms (arm 1: ChAdOx1-S prime & boost, arm 2: ChAdOx1-S prime and BNT162b2 boost). A comparison against arm 3 (BNT162b2 prime & boost) is exploratory and will be done descriptively.

In a study of Madhi [19] 92% of participants showed neutralizing antibodies following vaccination with ChAdOx1-S prime & boost against the B 1.1.7 (British) variant and 58% against the B.1.351 (South African) variant. Focusing on the improvement of the heterologous vaccination with the Biontech boost against the South African variant with regard to primary the endpoint of neutralizing antibodies we consider a sample size of n=1000 per study arm as appropriate. With this sample size, we can detect an increase in the percentage of participants with neutralizing antibodies against the South African variant from 58% to 65.5% as statistically significant, with a power of >90% and alpha of 5%. If the increase is 10% the statistical power would be >99%.

For the secondary endpoint of efficacy against breakthrough infection with the B.1.351 variant, based on the current number of 8 new cases per week in Tyrol with the B.1.351 variant we expect 208 cases during the next 180 days with no change in rate of infection.

Currently, the B.1.351 is concentrated in 4 districts – Kufstein, Schwaz, Innsbruck Land and Innsbruck Stadt giving a total population of 416,758, this gives a rate of 49,9/100,000 people in 180 days. As not all cases are sequenced we estimate that only 5% of B.1.351 variants are detected, this would mean in total a rate of approximately 1000 cases per 100000 population of the B.1.351 variant would occur over 180 days – this means 10 cases per 1000. This scenario could lead to 10 cases in the ChAdOx1-S prime & boost arm (Arm 1) and 0 case in the ChAdOx1-S prime and BNT162b2 boost arm (Arm 2). In the extreme situation that all 10 cases occur in Arm 1, this would be statistically significant. However, due to the decreasing infection rates in Tyrol we would currently consider this an underpowered analysis and as such would perform a posthoc power analysis. In addition, a second immune escape variant, B.1.1.7.+E484K has recently occurred in Tyrol with over 900 cases until now. However, the development of this outbreak is currently not predictable.

11.2 Randomization

Randomization of two of the study arms (group A and group C) will be performed in a 1:1 ratio stratified by 1) study site and 2) sex (male/female).

The randomization code will be generated by the study statistician using permutated blocks and implemented into the electronic CRF system (Askimed) in a two-step process using an external randomization tool or paper based. Patients will be randomized after verification of inclusion and exclusion criteria as well as stratification status and after screening assessments have been performed. Randomization code will be strictly blinded to clinical investigators and participants in Arms 1 and 2 for 90 days after the boost.

For the T cell determination sample, only participants from the Innsbruck trial site will be included to prevent poor cell quality due to sample transport. The first 100 samples at Innsbruck trial site in each of the three arms will be selected for T cell analysis.

Following the third immunization between day 200 and 260, 100 subjects will be selected for T cell analysis. Preferentially, subjects for which the T cell response had already been determined after boost immunization will be selected.

11.3 Statistical Design, Methods and Analysis process

11.3.1 Study Design

This is a multi-center, single-blinded, three-arm, randomized clinical trial in a parallel group design, after discontinuation of the homologous Vaxzevria arm a multi-center, non-blinded, two-arm, non-randomized clinical trial in a parallel group design.

11.3.2 Definition of Analysis Populations

The following populations will be used for statistical analysis

Safety population
The safety population will include all patients who received at least one boost vaccination.
In the safety analyses, patients will be analysed by vaccine received, regardless of any errors of dosing or vaccine administration.

Intention-to-treat population
The intention-to-treat (ITT) population will consist of all patients. Analyses of this population will assign patients the vaccine they were scheduled to receive.

Per-protocol population
The per-protocol (PP) population will include all patients who received the boost vaccination and fulfilled requirements up to day 30 after the boost. Major protocol deviations will be determined and documented prior to database lock.

T cell determination population
The first 100 participants at Innsburck study site will be selected in each arm for T cell determination. T cell responses will be analyzed in all non-responders after the third vaccination and for 100 subjects in each group after the third vaccination following completion of the follow-up period of 180 days.

11.3.3 Interim Analysis

Two interim analyses are scheduled applying the Haybittle Peto rule for early stopping [1]. The first interim analysis will be performed on day 30 for safety and efficacy when n=200 patients have been randomized. The second interim analysis will take place on day 30 after n=400 patients have been randomized. The trial may be stopped early for efficacy regarding the primary endpoint (level of neutralizing antibodies) when the p-value between the two randomized arms in smaller than 0.001 at either the first or the second interim analysis. If one of the two randomized arms is inferior, this arm will be closed and the study will continue in an open, non-blinded and non-randomized design.

Additionally interim analysis can be requested by the Data Safety and Monitoring Board (DSMB).

The number of individuals eligible and willing to a third vaccination at the two potential time points (between day 90 and 180 or after day 180) cannot be foreseen at this point. Therefore, each participant may be analyzed after reaching day 30 individually and efficacy after second and third vaccine dose will be compared at an individual level (before and after design).

11.3.4 Handling of missing, unused or spurious Data, including Drop-outs and Withdrawals

Missing data will be documented and, as far is possible, reasons for missingness will be evaluated. In case of substantial amounts of missing data >5%, imputation methods will be considered such as Hot Deck imputation.

11.3.5 Data Analysis

Efficacy analysis
Efficacy analyses are performed for the comparison of neutralizing titers against the wild type and escape variants at 30 days (primary endpoints of the study) and on breakthrough

infections of wild type/ escape variants (secondary endpoint) at 180 days. The main efficacy analysis is performed between the two randomized arms (Arm C: ChAdOx1-S prime & boost; Arm A: ChAdOx1-S prime and BNT162b2 boost).

Neutralising titer and breakthrough infection are both defined as binary quantitative variables.
Hypothesis testing between the two treatment arms will be performed using Chi^2 testing and logistic regression including stratification variables.

Efficacy analyses will be performed on the ITT and PP populations. The analysis of the ITT population will be considered as the primary analysis. All statistical tests are two-sided with asignificance level of 0.05.

Comparability of treatment arms
The two randomized and the third non-randomized vaccination arms will be assessed descriptively for comparability of demographic and baseline characteristics. Administered study vaccine, medical history, disease duration, use of prior treatments and concomitant medications will be summarised by treatment arm using descriptive statistics.

T Cell analysis (pre-defined subgroup analysis)
Efficacy analyses are performed for the comparison of Quantiferon results by treatment arm.

Safety Analysis
Safety evaluations will be based on the incidence, type, severity and consequences (e.g. study discontinuation) of an adverse event (AE) as well as on clinically significant changes in the patient's physical examination, vital signs, and clinical laboratory results (including immunotoxicity). Statistical analysis includes tabulation per treatment group using descriptive measures which are absolute and relative frequencies for categorical data and means, standard deviations, medians and interquartile ranges for continuous data.
All safety analyses will be performed on the safety population.

All analyses will be performed in SPSS 26.0 or StataMP 16.0.

12. Quality Management

Training, monitoring and audits are performed for quality assurance within this clinical study. Monitoring and auditing procedures developed or endorsed by the sponsor will be conducted, in order to comply with ICH-GCP guidelines and local legal requirements to ensure acceptability of the study data.

12.1 Qualifications

The sponsor is responsible for selecting the investigator(s)/institution(s). Each investigator should be qualified by training and experience and should have adequate resources. Each

individual involved in conducting a trial should be qualified by education, training and experience to perform his or her respective task(s) (see ICH GCP E6).
To meet those objectives, describe types and methods of investigators and involved members of the study team (training on GCP-guidelines, trial protocol, completion of the e-CRF, etc.).

12.2 Monitoring

The exact extent of the monitoring procedures including the strategy for source data verification is described in a separate monitoring manual. A brief summary should be given, where basic monitoring activities and possible central measures taken to ensure quality across trial sites as well as information pathways are described.
The trial sites will be monitored to ensure the quality of the collected data. The objectives of the monitoring procedures are to ensure that the trial subject's safety and rights as a study participant are respected, that accurate, valid and complete data are collected, and that the trial is conducted in accordance to the trial protocol, the principles of GCP and local legislation.
All investigators agree that the monitor regularly visits the trial site and assure that the monitor will receive appropriate support for the tasks at the trial site, as agreed in separate contracts with each trial site. The informed consent form (ICF) includes a statement that the monitor has the right – while observing the provisions of data protection legislation – to compare CRFs with the trial subject's medical records (doctor's notes, ECGs, laboratory printouts etc.). The investigator will secure access for the monitor to all necessary documentation for trial-related monitoring.
A study specific monitoring plan will be established and the study will be monitored with the agreed plan.

12.3 Audits and Inspections

Regulatory authorities, ethics committees, and Sponsor's delegates may perform on-site inspections or audits, for which the Investigator must provide support at all times.
During an audit following issues among other things will be inspected:

- performance of the clinical trial according to the investigation plan
- data validity
- quality of the clinical trial according to the ICH E6 guidelines

After each external audit an audit-certificate by the auditor has to be sent to the investigator. This certificate has to be kept in the ISF to evidence the audit to the regulatory authorities in the case of an inspection by them. The audit-report is sent to the sponsor of the study. An audit-certificate will be attached to the final report at the end of the study. Additionally, according to the AMG audits and inspections by regulatory authorities may be performed.

13. Reporting

For the documentation of the progress and development of the study, protocols about the meetings of the various group committees are necessary.

13.1 Final Study Report

All information regarding this clinical study has to be kept in confidence. The statistical analysis and the integrated final study report will be prepared according to ICH E6 and finalized within 12 months after the last patient last visit (LPLV) took place. The final study report will be reviewed and signed by the sponsor, the coordinating investigator and all further responsible persons. All information in that report is strictly confidential.

13.2 Publication

The presentation of the results in a publication should have the extent of the defined framework of the CONSORT-Statement (www.consort-statement.org). All randomized and controlled clinical studies, which recruit patients after the 1st of July 2005, have to be registered in a publicly available data base (e.g. www.clinicaltrials.gov, www.controlled-trials.com). This is necessary to publish the study results in a renowned journal.

14. Amendments

After the protocol has been submitted to an ethics committee (EC), any substantial change will require a formal amendment. The amendment must be signed by all of the signatories to the original protocol. Once the study has started, amendments should be made only in exceptional cases. The ethics committees must be informed of all amendments. If necessary, approval must be sought for ethical aspects and must also be obtained from the competent authorities.

15. Ethical and Regulatory Aspects

15.1 Responsibilities of Sponsor and Investigator

The sponsor of this clinical trial will assume responsibility for inducement, organization and financing of the implementing trial according to the ICH E6. The procedures set out in this study protocol are designed to ensure that the sponsor and the Investigator comply with the principles of ICH-GCP the Declaration of Helsinki and the ICH E6 guideline concerning the conduct, evaluation and documentation of the study. The study will also be performed adhering the local legal conditions and requirements. Each Investigator has to confirm this by signing the study protocol.
Responsibilities of the sponsor:

- verification of the understanding of the investigator´s brochure or the described IMPD
- verification of the understanding of treatment schedule

- ensuring for enough time and capacity for the implementation of this study
- correct collection and documentation of data, reporting
- provision of all data to the sponsor, monitor or relevant authorities for audits or inspections
- assurance for the confidential handling of patients data and information

The principal investigator accepts the responsibility for the conduct of this clinical trial at this study site according to the ICH E6

15.2 Approval of Ethics Committee and Notification to the Authority

Prior to study start, the study protocol and/or other appropriate documents will be submitted to the relevant ECs and CAs for approval. Approval from all concerned ECs and CAs must be obtained before starting the study.

15.3 Patient Information and Consent Form

Every patient has to give his/her written consent BEFORE the participation in the clinical trial. Before the patient gives his/her written consent the patient has to be informed completely in oral and written form in an understandable manner about character, importance, relevance and consequences of the clinical trial.
The content of the consent information is documented on the patient information/ informed consent form. The patient will be notified, if essential findings about the MD appear during the study.
The informed consent of the patient about the participation in the clinical trial has to be dated and signed by the patient and the Investigator. The patient receives a copy of the signed and dated Patient information and informed consent Form. The Investigator stores the original signed and dated exemplar in the investigator site file.
It has to be explicitly pointed out, that before patient sign the informed consent form it is not allowed to perform any study specific actions with the patient.

15.4 Insurance

The sponsor will take out reasonable third-party liability insurance cover in accordance with local legal requirements. The civil liability of the investigator, all persons instructed and the hospital, practice or institute in which they are employed and the liability of the sponsor in respect of financial loss due to personal injury and other damage that may arise as a result of the carrying out of this study are governed by the applicable local law.
The sponsor will arrange for patients participating in this study to be insured against financial loss due to personal injury caused by the study medication or by medical steps taken in the course of the study. Such insurance is taken out by the sponsor in accordance with or by way of analogy to both the Austrian and the other participating countries pharmaceutical act.

15.5 Data Protection and Confidentiality

All local legal requirements regarding data protection will be adhered to. All study findings and documents will be regarded as confidential. The Investigator and members of the research team must not disclose any information without prior written approval from the sponsor.
The pseudonymity of patients participating must be maintained. Throughout documentation and evaluation, the patients will be identified on CRFs and other documents by name and date of birth. Documents that identify the patient personally (e.g., the signed informed consent, patient identification list) must be maintained in confidence by the Investigator. The patients will be informed in the ICF that all study findings will be stored on computer and handled in strictest confidence.

15.6 Financing

There is no particular funding for the present clinical trial.

15.7 Regulatory Aspects

The processes set out in this study protocol are designed to ensure that the sponsor and the Investigator abide the principles of the ICH E6 and the Declaration of Helsinki concerning the conduct, evaluation and documentation of the study. The study will also be performed in compliance with the local legal conditions and requirements. Each investigator has to confirm this by signing the study protocol.

16. DSMB

A DSMB will be established to monitor safety data on an ongoing basisvto ensure the sontinuing safety of the participants enrolled in this study. This committee will review interim unblinded data. The DSMB responsibilities, authorities and procedures will be documented in the DSMB Charter.
Members are:
Florian Krammer, New York
Marton Szell, Vienna
Michael Kundl, Vienna

17. Literature

1. Schulz KF, Grimes DA. Multiplicity in randomised trials II: subgroup and interim analyses. Lancet 2005;365:1657-61.
2. Kustin T, Harel N, Finkel U, et al. Evidence for increased breakthrough rates of SARS-CoV-2 variants of concern in BNT162b2 mRNA vaccinated individuals. medRxiv 2021:2021.04.06.21254882.
3. Borena W, Bánki Z, Bates K, et al. Follow-up study in the ski-resort Ischgl: Antibody and T cell responses to SARS-CoV-2 persisted for up to 8 months after

infection and transmission of virus was low even during the second infection wave in Austria. medRxiv 2021:2021.02.19.21252089.
4. Rophina M, Pandhare K, Shamnath A, Imran M, Jolly B, Scaria V. ESC - a comprehensive resource for SARS-CoV-2 immune escape variants. bioRxiv 2021:2021.02.18.431922.
5. Jackson LA, Anderson EJ, Rouphael NG, et al. An mRNA Vaccine against SARS-CoV-2 - Preliminary Report. N Engl J Med 2020;383:1920-31.
6. Anderson EJ, Rouphael NG, Widge AT, et al. Safety and Immunogenicity of SARS-CoV-2 mRNA-1273 Vaccine in Older Adults. N Engl J Med 2020;383:2427-38.
7. Widge AT, Rouphael NG, Jackson LA, et al. Durability of Responses after SARS-CoV-2 mRNA-1273 Vaccination. N Engl J Med 2021;384:80-2.
8. Mallapaty S. Israel is first to see COVID-infection drop from vaccines. Nature 2021;590:197.
9. Madhi SA, Baillie V, Cutland CL, et al. Safety and efficacy of the ChAdOx1 nCoV-19 (AZD1222) Covid-19 vaccine against the B.1.351 variant in South Africa. medRxiv 2021:2021.02.10.21251247.
10. Darby AC, Hiscox JA. Covid-19: variants and vaccination. BMJ 2021;372:n771.
11. Mizrahi B, Lotan R, Kalkstein N, et al. Correlation of SARS-CoV-2 Breakthrough Infections to Time-from-vaccine; Preliminary Study. medRxiv 2021:2021.07.29.21261317.
12. Utrilla-Trigo S, Jimenez-Cabello L, Alonso-Ravelo R, et al. Heterologous Combination of ChAdOx1 and MVA Vectors Expressing Protein NS1 as Vaccination Strategy to Induce Durable and Cross-Protective CD8+ T Cell Immunity to Bluetongue Virus. Vaccines (Basel) 2020;8.
13. Perdiguero B, Gomez CE, Garcia-Arriaza J, et al. Heterologous Combination of VSV-GP and NYVAC Vectors Expressing HIV-1 Trimeric gp145 Env as Vaccination Strategy to Induce Balanced B and T Cell Immune Responses. Front Immunol 2019;10:2941.
14. Hu Z, Jiang W, Gu L, et al. Heterologous prime-boost vaccination against tuberculosis with recombinant Sendai virus and DNA vaccines. J Mol Med (Berl) 2019;97:1685-94.
15. Yusuf Y, Yoshii T, Iyori M, et al. A Viral-Vectored Multi-Stage Malaria Vaccine Regimen With Protective and Transmission-Blocking Efficacies. Front Immunol 2019;10:2412.
16. Valdes I, Izquierdo A, Cobas K, et al. A heterologous prime-boost strategy for immunization against Dengue virus combining the Tetra DIIIC subunit vaccine candidate with the TV005 live-attenuated tetravalent vaccine. J Gen Virol 2019;100:975-84.
17. Yusuf Y, Yoshii T, Iyori M, et al. Adeno-Associated Virus as an Effective Malaria Booster Vaccine Following Adenovirus Priming. Front Immunol 2019;10:730.
18. Tiono AB, Nebie I, Anagnostou N, et al. First field efficacy trial of the ChAd63 MVA ME-TRAP vectored malaria vaccine candidate in 5-17 months old infants and children. PLoS One 2018;13:e0208328.
19. Madhi SA, Baillie V, Cutland CL, et al. Efficacy of the ChAdOx1 nCoV-19 Covid-19 Vaccine against the B.1.351 Variant. N Engl J Med 2021.
